# On the prebiotic selection of nucleotide anomers: A computational study

**DOI:** 10.1016/j.heliyon.2022.e09657

**Published:** 2022-06-09

**Authors:** Lázaro A.M. Castanedo, Chérif F. Matta

**Affiliations:** a Department of Chemistry, Saint Mary's University, Halifax, Nova Scotia, B3H 3C3, Canada; b Department of Chemistry and Physics, Mount Saint Vincent University, Halifax, Nova Scotia, B3M 2J6, Canada; c Department of Chemistry, Dalhousie University, Halifax, Nova Scotia, B3H 4J3, Canada; d Dép. de chimie, Université Laval, Québec, Québec, G1V 0A6, Canada

**Keywords:** Prebiotic chemistry, Nucleosides, Nucleotides, Uracil and thymine, Anomers of nucleosides and nucleotides, Density functional theory (DFT) calculations

## Abstract

Present-day known predominance of the *β*- over the *α*-anomers in nucleosides and nucleotides emerges from a thermodynamic analysis of their assembly from their components, *i.e.* bases, sugars, and a phosphate group. Furthermore, the incorporation of uracil into RNA and thymine into DNA rather than the other way around is also predicted from the calculations. An interplay of kinetics and thermodynamics must have driven evolutionary selection of life's building blocks. In this work, based on quantum chemical calculations, we focus on the latter control as a tool for “natural selection”.

## Graphical abstract

**Figure:**
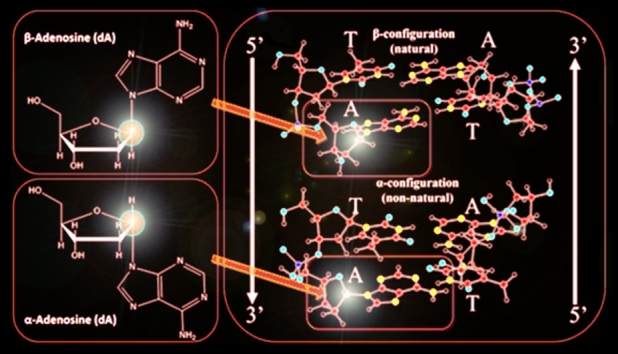

## 1 Introduction

**Figure 1:**
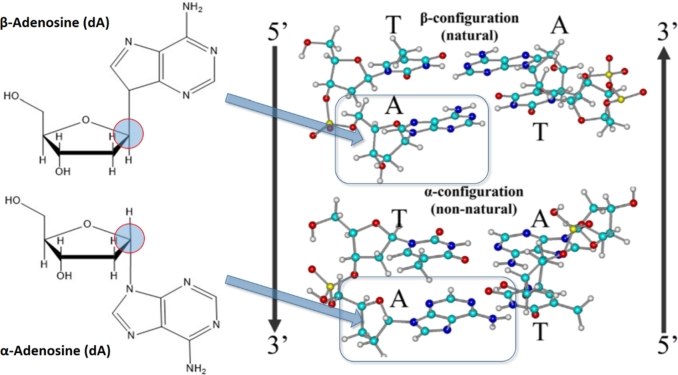
**Left: (*top*)** An example of a *β*-nucleoside (*β*-2′-deoxyadenosine), the form that predominates in present day nucleic acids, and **(*bottom*)** the corresponding *α*-isomer which is seldom observed. **Right: (*top*)** A representation of a present-day *β*-DNA Watson-Crick (WC) double-helix, and **(*bottom*)** a model constructed using molecular builders (*HyperChem*/*GaussView*) demonstrating the perfect *geometric* WC base pairing in the non-predominant form of *α*-DNA.

On what basis did early prebiotic conditions favor the selection and assembly of particular building blocks of nucleic acids? An aspect of this question is the subject of the present investigation. The broader aspects of this question are well-documented as, for instance, reviewed by Šponer et al. (Šponer et al., 2011, Šponer et al., 2016) and by Serrano Giraldo and Zarante (Serrano Giraldo and Zarante, 2021), but here we restrict ourselves to a narrower question. Specifically, the majority of contemporary natural nucleic acids, whether DNA or RNA, are polymers of nucleotides in the *β*-configuration at the C1′ carbon of the furanose sugar and seldom in the *α*-configuration (Fig. 1), but why? The question is amplified by the ease by which anomers can be synthesized and by their similar ability to form Watson-Crick double helices (Fig. 1).

Another explored question is the factors that might have driven the selection of thymine for incorporation into DNA and for uracil into RNA. After all, the switching of the bases and sugars seems to be equally likely. Nucleosides, as predominantly existing in today's genetic material, are 2′-deoxythymidine (dT) in DNA and uridine (U) in RNA rather than as T and dU. Why?

Thermodynamic and kinetic control drive chemical reactions. In this paper, the role of thermodynamics as a driver of evolutionary selection is being explored. The idea of thermodynamics-driven natural selection has been applied, for example, to explain the origin of the genetic code by Grosjean and Westhof (Grosjean and Westhof, 2016) and by Klump, Völker, and Breslauer (Klump et al., 2020). Here, this line of thought is extended to inquire whether the present-day forms of the building blocks of nucleic acids are, at least, aligned with such an energetic argument.

Clearly a natural thermodynamic selection of the *β*- over the *α*-configuration or of the “correct” choice of U/T for the proper nucleic acid category, represent restricted questions from a vast repertoire of possible ones. For example, one may wish to inquire into the evolutionary pressures that picked the present-day *particular arrangement* of nucleic acids in terms of a pentose sugar, a phosphate group, and a nitrogenous base – rather than – say – having a 2-aminoethyl glycine as a linker, as occurs in peptide nucleic acids (PNAs) (Pellestor and Paulasova, 2004), instead of the phosphodiester backbone (Banack et al., 2012)? No one knows. One can question Nature's Central Dogma (DNA↔DNA→RNA→protein) (Crick, 1970) and whether this is the only conceivable way to produce living systems, etc. Clearly these wider questions are of utmost importance to understand the origins of life but are vastly larger than the scope of this investigation.

In 1955, Kaplan et al. (Kaplan et al., 1955) reported the isolation of a compound that had the same composition as nicotinamide adenine dinucleotide (NAD^+^), the latter was termed diphosphopyridine nucleotide at the time. Subtle deviations in the properties of this compound from those anticipated for NAD^+^ led these authors to conclude that it is an isomer of NAD^+^. Indeed, the compound discovered in 1955 is the *α*-isomer of the NAD^+^ (which has a *β*-configuration at the glycosidic bond) (Kaplan et al., 1955). A decade later, Suzuki and co-workers (Suzuki et al., 1965) isolate bacterial *Azotobacter vinelandii α*-NAD, *α*-nicotinic acid adenine dinucleotide, *α*-NADP, and *α*-nicotinic acid mononucleotide. The latter work shows that, while much less frequent, the *α*-form does occur indeed in living systems (Suzuki et al., 1965).

Paoletti et al. report the experimental synthesis of an *α*-*β* complex between a *α*-d(CCTTCC) hexanucleotide and its complementary *β*-d(GGAAGG) (Paoletti et al., 1989). A comparison of the formation of this complex with its natural *β* analog (*β*-d(CCTTCC) + *β*-d(GGAAGG)) reveals that the formation of the non-natural form is only ≈1 kcal/mol more favored than its natural counterpart (Paoletti et al., 1989). There are other reports of synthesis of nucleic acids containing *α*-nucleic acids strands (Froeyen et al., 2001; Guesnet et al., 1990; Lancelot et al., 1987, Lancelot et al., 1989).

Kaur, Sharma, and Wetmore (KSW) have proposed barbituric acid and melamine (Kaur et al., 2017) and cyanuric acid (CA) and 2,4,6-triaminopyrimidine (TAP) (Kaur et al., 2019) as precursors of prebiotic RNA on the basis of quantum mechanical calculations and molecular dynamics (MD) simulations. In their more recent paper (Kaur et al., 2019), KSW use density functional theory (DFT) calculations to obtain potential energy surfaces describing the rotation around the glycosidic bonds of *β*- and *α*-ribonucleosides of the non-canonical nucleobases TAP and CA as well as their complementary base pairing TAP:CA and stacking energies. Additionally, these authors studied the base pairing of these nucleobases with the canonical nucleobases (A, G, C, T and U) and compared the canonical 10-mer A-form of RNA duplexes 5′-AAAAAAAAAA-3' paired with 5′-UUUUUUUUUU-3′ and 5′-AAAGCGCAAA-3′ paired with its complementary 5′-UUUCGCGUUU-3′ with the non-canonical duplexes 5′-AAAXXYYAAA-3′ paired with 3′-UUUYYXXUUU-5', where X = CA and Y = TAP. The results obtained suggest that the strength for the hydrogen bonds created in the TAP:CA pairing is comparable to the canonical base pairing. The stacking of these non-canonical bases is, on the other hand, weaker compared to the canonical stacking, suggesting that the enhanced stacking may have served as a driving force in the evolution of nucleic acids. Finally, the assembled structure of TAP-CA-containing helices suggests that this type of pre-RNA could have been shielded from water allowing its evolution and self-replication. The results in that paper, (Kaur et al., 2019), place TAP and CA as plausible candidates for a pre- or proto-RNA.

In their earlier study, KSW computational results on barbituric acid (BA) and melamine (MM) suggest their plausibility as non-canonical nucleic acid bases that may have been present in the precursor of present day nucleic acids (Kaur et al., 2017). The authors find that the strength of the hydrogen bonds between BA and MM makes them good candidates as building blocks of ancestral nucleic acids. On the other hand, they find that the stacking interactions were stronger when either BA or MM are combined with a canonical nucleobase than when the stacking was between each other. These results suggest the possibility of the existence of a pre- or proto-RNA that mixes canonical and non-canonical complementary nucleobases within one structure (Kaur et al., 2017).

In their first paper, KSW report that the potential energy hypersurface of breaking the glycosidic bond is consistent with a stronger bond in TAP nucleosides compared to canonical nucleosides. Interestingly, in the case of the CA the opposite result is obtained (Kaur et al., 2019). KSW found larger deglycosylation barriers for the C-C glycosidic bond of BA-ribonucleosides compared to canonical nucleobases while the reverse occurs in the case of MM (Kaur et al., 2017).

The biopolymers of life are believed to have emerged between 3.5 and 4 billion years ago (Kitadai and Maruyama, 2018; Sutherland and Whitfield, 1997), with details still to be worked-out. For instance, which was first: Proteins or nucleic acids? The consensus is that nucleic acids were probably the first biomolecules, specifically RNA in what is commonly known as the “RNA world hypothesis” (Cech, 2012; Gesteland et al., 1999), since RNA is both a catalyst and a repository of genetic information making it candidate for the first biopolymers (Ayukawa et al., 2019; Neveu et al., 2013; Orgel, 2004).

If we accept that RNA came first, then other questions arise. For example, how did this molecule originate in the first place? It has been proposed that nucleic acids were the product of prebiotic and geochemical reactions, namely, the “*drying pool*”, “*drying lagoon*”, also known as the “*classic model*”, whereby regular cycles of dehydration-re-hydration can promote the polymer formation. Given the important role of the so-called “water problem” in early evolution, whereby H_2_O impedes the synthesis of nucleic acids (do Nascimento Vieira et al., 2020; Hud et al., 2013; Joyce and Orgel, 1999; Kim et al., 2016), in this work both vacuum phase and aqueous phase calculations were conducted.

Within the classic model, Orgel and coworkers explored the formation of the glycosidic bond between purines (adenine, guanine, inosine, xanthine) and ribose sugar by drying and heating the reactants in the presence of catalysts (Fuller et al., 1972). Only adenine was found to couple with the ribose to produce *α*- and *β*-furanosil nucleosides with yields typically within ≈2–10%. The relatively low yield by Orgel and coworkers were attributed to the instability of the glycosidic bond in aqueous environment giving rise to what is known as the “nucleoside problem” (a special case of the more general “water problem”) (do Nascimento Vieira et al., 2020; Hud et al., 2013; Joyce and Orgel, 1999; Kim et al., 2016). Challenges including the nucleoside problem have led scientists to look for alternative synthetic routes that start, for example, from phosphorylated sugars and free bases (Bernhardt and Sandwick, 2014; Crowe and Sutherland, 2006; Gull et al., 2017; Ingar et al., 2003; Kim and Kim, 2019; Zubay and Mui, 2001).

The hypothesis being tested here is that Nature's stereo-selection of the present-day canonical nucleosides/nucleotides is consistent with an energy-driven natural selection. Thus, the two anomeric forms of the nucleosides/nucleotides were studied as they occur within both DNA and RNA and compared for their thermodynamic stabilities.

In final account, since the deamination of cytosine transforms it into uracil with a consequential change in the genetic message, selection pressures may have driven the transition from uracil to thymine in DNA (Poole et al., 2001). This question has long been raised (Lesk, 1969) but appears to have never been resolved on the grounds of relative total molecular energies to the best of the authors' knowledge. Since there appears to be no *a priori* reason for Nature to have selected thymine to be incorporated exclusively in DNA and uracil in RNA (with exceptions), mixed (“wrong”, or non-canonical) nucleotides have also been considered in our calculations as an available choice for natural selection.

To sum-up, we observe a preponderance of *β*- over the *α*-anomers in present-day nucleosides(tides). This work indicates that such preponderance is consistent with thermodynamic parameters calculated quantum chemically within the assumptions and approximations of the study. The known specificity of uracil to RNA and thymine to DNA is also consistent with these results. While both kinetics and thermodynamics, and the interactions with solvent molecules and ions, must have all driven together evolutionary selection of life's building blocks, in this work we restrict the question as to whether there exists an inherent preference in the building block themselves that is consistent with the observed “naturally selected” present day nucleic acids.

## 2 Computational details

The structure of each nucleoside (sugar + nitrogenous base) in two anomeric forms (*β* and *α*) where constructed with the graphic interfaces of Hyperchem 7.0 (1996) and GaussView 5.0 (Dennington et al., 2009). The initial 20 nucleosidic structures {[(2 sugars (ribose, and 2′-deoxyribose)) × (5 bases (adenine (A), thymine (T), guanine (G), cytosine (C), and uracil (U)))] × 2 (*β* and *α*)} were subjected individually to a soft potential energy hypersurface (PES) scan with respect to the angle that governs the N-glycosidic bond between the base and the sugar. “Soft scan” means that the only constrain is the angle being scanned stepwise while all other geometrical parameters are allowed to relax in response to that angle.

The above-mentioned PES scans were performed in the following way. *Z*-matrices for the ribofuranose and 2′-deoxyribofuranose sugar, each in both the *β* and *α* configuration, were read into the programme Granadarot (Montero, 2019; Montero et al., 1998). The Granadarot algorithm was used to create, for the ribofuranose, 1,000 different conformers by randomly varying the five dihedral angles that involve all the sugar's hydroxyl groups (4 angles of the H-O-C-C type, in addition to the O-C5′-C4′-C3′ angle – also known as *β* angle (not to be confused with the anomeric label)). For the 2′-deoxyribofuranose, a similar procedure was used to also generate 1,000 conformers with the difference that now there are only 3 angles of the H-O-C-C type. The number of initial random conformers (1,000) strikes a balance between a good sampling of the PES and computational costs.

For each of these initial randomized sugar structures (4,000 in total), the geometries were optimized at the semiempirical PM7 level of quantum chemical theory, a method that includes empirical corrections for dispersion and hydrogen bonding interactions (Stewart, 2007, Stewart, 2013). By including these corrections, PM7 has an important advantage over other semiempirical methods that generally do not account for dispersion and hydrogen bonding explicitly. These PM7 geometry optimizations were conducted through a gradient minimization of the total energy using the MOPAC2016 package (Stewart, 2019) until the forces on the nuclei were negligible. These geometry optimizations were performed twice: Once in vacuum phase and a second time with the COSMO continuum solvation model (Klamt and Schüümann, 1993).

Several of the sets of 1,000 optimizations described above converge to one and the same respective final geometry. The number *n* of final unique optimized geometries is: *In vacuum: β*-ribofuranose (n=28), *α*-ribofuranose (n=34), *β*-2′-deoxyribofuranose (n=42), *α*-2′-deoxyribofuranose (n=16); *in solvent: β*-ribofuranose (n=110), *α*-ribofuranose (n=84), *β*-2′-deoxyribofuranose (n=78), *α*-2′-deoxyribofuranose (n=65).

For each of one of these 8 systems, the $n^{\prime }$ conformers that collectively contribute at least 50% to the partition function (*Z*) were kept for refinement with more accurate calculations and the rest of the conformers with minor contributions were discarded. This reduced the number of investigated conformers to: *In vacuum: β*-ribofuranose ($n^{\prime }=4$, contributing 59.5% of *Z*), *α*-ribofuranose ($n^{\prime }=2$, contributing 60.4% of *Z*), *β*-2′-deoxyribofuranose ($n^{\prime }=3$, contributing 54.6% of *Z*), *α*-2′-deoxyribofuranose ($n^{\prime }=1$, contributing 51.0% of *Z*); *in solvent: β*-ribofuranose ($n^{\prime }=12$, contributing 51.4% of *Z*), *α*-ribofuranose ($n^{\prime }=12$, contributing 51.6% of *Z*), *β*-2′-deoxyribofuranose ($n^{\prime }=7$, contributing 53.0% of *Z*), *α*-2′-deoxyribofuranose ($n^{\prime }=7$, contributing 51.9% of *Z*).

The geometry of every sugar structure of the $n^{\prime }$ that survived the initial screening, was re-optimized without constraints at the density functional level of theory (DFT) (Koch and Holthausen, 2001; Parr and Yang, 1989; Scholl and Steckel, 2009) using the B3LYP/6-31G(d,p) functional (Becke, 1993; Lee et al., 1988) / basis set (Hehre et al., 1986) combination. Aqueous solvation was accounted for in the DFT calculations using an integral equation formalism variant of the “*polarizable continuum model*” (IEFPCM) (Miertus et al., 1981; Tomasi et al., 2010; Tomasi et al., 2005) implemented in Gaussian 16 (Frisch et al., 2019), the software package used in all DFT calculations in this work.

The so-called “water problem” (Joyce and Orgel, 1999) describes the consensus understanding that the primordial soup has been non-polar in nature or, at least, had a controlled exposure to water (see for example Ref. (do Nascimento Vieira et al., 2020) and literature cited therein). Hence, the primary results to be considered and discussed here are those in the vacuum phase as a surrogate for non-polar environment. Solvation modeling has been included, however, to also test the effects of this very “water problem” but also for completion since aqueous medium predominates in contemporary living systems.

Solvation remains an open problem for quantum chemical calculations. One has to pick from the dichotomy of *explicit solvation* or the modeling of its effects by placing the solvent in a cavity within a bulk dielectric continuum, that is, *implicit solvation* (Cramer, 2002; Cramer and Truhlar, 1995; Cramer and Truhlar, 1999; Foresman and Frisch, 1996; Marenich et al., 2009; Miertus et al., 1981; Tomasi et al., 2010; Tomasi et al., 2005; Tomasi and Persico, 1994). Explicit solvation is best to describe strong localized interactions between the solute molecule and immediate solvation shell molecules, while continuum solvation modeling is better tuned to capture the long-range averaged effects of solvation by the solvent bulk. Explicit solvation, ideally, entails a gradual addition of solvent molecules until the convergence of some relevant parameters, which is impractical here in view of the large number of studied systems. Hence, the second best option, that is, the continuum modeling, has been applied in this work.

Every geometry optimization in this work has been followed by a harmonic vibrational analysis and all were found to exhibit no imaginary frequency as required to confirm their status as local minima on the PES. For each one of the eight groups described above, the most stable structure of the sugar that resulted from the DFT optimization was saved for the next step and the rest of the structures were discarded.

The five nucleobases (A, G, C, T, U) were optimized at the DFT-B3LYP/6-31G(d,p) level of theory, in a similar procedure as the one outlined above for the sugars, in vacuum and in solvent phase. These optimized structures were then stitched to the sugars leading to 40 separate initial N-nucleoside guess geometries (5 bases × 2 sugars × 2 configurations × 2 phases/environments).

For consistency, the N-glycosidic bond, C1′-N1 in pyrimidines (Y) or C1′-N9 in purines (R) was initially set to 1.52 Å while the dihedral angle H-C1′-N1(Y)/N9(R)-C*x* was set initially at −161.9^∘^. Each of the 40 nucleoside structures was then subjected to a fully relaxed scan around this dihedral in 6 steps each of 60^∘^. The lowest structure from this scan was refined by subjecting it to a final fully-unconstrained optimization to obtain the final structure of the nucleosides in vacuum and in solvent. A harmonic frequency calculation was performed as usual to ensure that the final structures are indeed minima on the PES.

Finally, a mono-anionic dihydrogen phosphate group (H_2_PO${}_{4}^{-}$), the form dominant at pH 6.5 (Sponer et al., 2011), has been optimized unconstrained both in the vacuum phase and in continuum solvent at the DFT-B3LYP/6-31G(d,p) level of theory. The optimized phosphate was then attached to the nucleosides at C5′-OH setting the initial O-C5′-C4′-C3 torsion angle to 30.9^∘^ (the standard angle for the stored structures in GaussView). A soft scan was then performed in 6 steps of 60 degrees, and again the lowest energy conformer of the nucleotide was retained for further analysis. The procedure outlined above yields 80 final optimized structures: 40 for each class (nucleoside, and nucleotide), 20 in gas- and 20 in solution-phase.

The steps described above proceed in the following order: *sugar* + *base* → *nucleoside* followed by a geometry optimization of the nucleoside, and then by the reaction *nucleoside* + *phosphate* → *nucleotide* followed by a geometry optimization of the nucleotide. Since every step of these two concatenated “reactions” is followed by a geometry optimization, a change in the order of these reactions does not necessarily yield the same geometries (and energies). Hence, the procedure described so far has been repeated except by reversing the order of the reaction, that is: *sugar* + *phosphate* → *5'sugar-monophosphate* → *optimization* → *5'sugar-monophosphate* + *base* → *nucleotide* → *optimization*.

For a given pair of *α*/*β*-anomers, the difference in their energies (relative energy) is defined as (equation (1)):

(1) $$\Delta X_{\beta \alpha }\equiv X_{\beta }-X_{\alpha },$$

where Δ*X* denotes Δ*E* (the difference is of the total energies), or Δ*E*_(ZPE)_ (the difference in the total energies corrected for zero-point vibrational energies (ZPEs)), or $\Delta G^{\circ }$ (the difference in the Gibbs energies at standard conditions). The inclusion of solvation effects will be indicated when necessary using extra symbols.

The DFT-B3LYP/6-31G(d,p) level of theory has been chosen for this study as a reasonable compromise of accuracy and speed/feasibility. The error bars for a similar level of theory, namely, DFT-B3LYP/6-31+G(d,p) have been benchmarked by Zhao and Truhlar's to be around 3.6 kcal/mol (Zhao and Truhlar, 2008). Zhao and Truhlar obtained this estimate by comparing the calculated and experimental thermodynamic data for 177 main-group compounds (Zhao and Truhlar, 2008). On that basis, we may take the intrinsic uncertainty of the method used in this work to be around ≈3 – 4 kcal/mol.

## 3 Results and discussion

### 3.1 Two hypothetical synthetic pathways and their Gibbs energies

**Figure 2:**
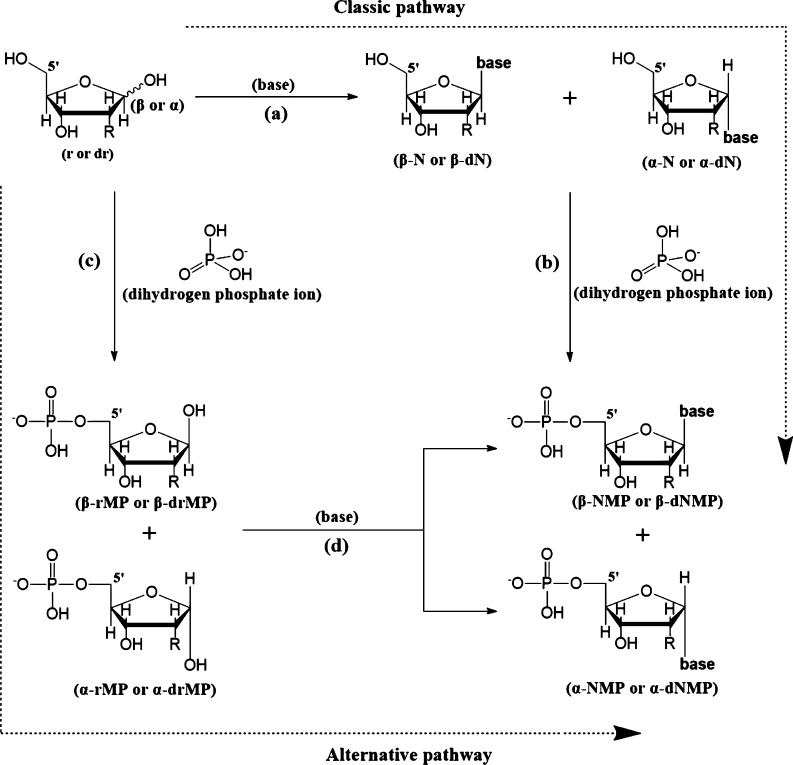
Two different pathways for constructing the *β*- and *α*-anomers of nucleosides and nucleotides. (Reaction pathways referred-to in the text and tables are labeled with lower-case letters: Classical pathway (a + b) and alternative pathway (c + d); R=H,OH for DNA and RNA, respectively).

The two pathways of the formation of a nucleotide are depicted in Fig. 2 and are *not* equivalent. That non-equivalency is due to the effect of the first condensation on geometry which leads to final products trapped at different wells on the potential energy surface of the nucleotide. In other words, (a + b) and (c + d) are *different* pathways with *different* $\Delta G_{\mathrm{reaction}}^{\circ }$. Recapping, the two condensation sequences considered are:

(1) The condensation of a sugar with a base to obtain the N-nucleoside *followed* by the condensation of the nucleoside with a dihydrogenphosphate anion (H_2_PO${}_{4}^{-}$) at the 5′ position to obtain the nucleotide. The Gibbs free energy of this reaction is defined as (equation (2)):
  (2) $$\Delta G_{\begin{array}{c} \mathrm{reaction}\\ \left(a+b\right) \end{array}}^{\circ }=\left[\left(G_{\mathrm{nucleoside}}^{\circ }+G_{H_{2}O}^{\circ }\right)-\left(G_{\mathrm{sugar}}^{\circ }+G_{\mathrm{base}}^{\circ }\right)\right]\;+\left[\left(G_{\mathrm{nucleotide}}^{\circ }+G_{H_{2}O}^{\circ }\right)-\left(G_{\mathrm{nucleoside}}^{\circ }+G_{H_{2}{\mathrm{PO}}_{4}^{-}}^{\circ }\right)\right].$$
(2) The condensation reaction of a H_2_PO${}_{4}^{-}$ group with a sugar at C5′ first, *followed* by a condensation of the sugar 5′-monophosphate with the base. In this case the Gibbs energy of reaction is defined as (equation (3)):
  (3) $$\Delta G_{\begin{array}{c} \mathrm{reaction}\\ \left(c+d\right) \end{array}}^{\circ }=\left[\left(G_{H_{2}O}^{\circ }+G_{\mathrm{sugar}5^{\prime }-\mathrm{monophosphate}}^{\circ }\right)-\left(G_{\mathrm{sugar}}^{\circ }+G_{H_{2}{\mathrm{PO}}_{4}^{-}}^{\circ }\right)\right]\;+\left[\left(G_{\mathrm{nucleotide}}^{\circ }+G_{H_{2}O}^{\circ }\right)-\left(G_{\mathrm{sugar}5^{\prime }-\mathrm{monophosphate}}^{\circ }+G_{\mathrm{base}}^{\circ }\right)\right].$$

### 3.2 Which furanose or furanose-phosphate anomers are more stable: *α* or *β*?

As a prelude to this study, we first revisit the relative stabilities of the furanose forms of the sugars themselves. A ^13^C NMR study complemented with a statistical mechanics analysis by Dass et al. demonstrates a strong temperature-(gradient)-dependence of the equilibrium ratios of the various open-chain and cyclic forms of ribose sugar (Dass et al., 2021). These authors are simulating the conditions of temperature and temperature-gradient near hydrothermal vents to answer the question of which form(s) of the ribose sugars were favored at early prebiotic times. From these authors' Fig. 2 (Dass et al., 2021), in pure aqueous solution at 25 °C, the *β*-*pyranose* (*β*P-form) is dominant with a mole fraction of ≈0.6, followed by the *α*P-form with a mole fraction of ≈0.2. These authors' figure indicates much lower populations for the two furanose forms under these conditions, both anomers having similar mole fractions of ≈0.1 each. This means that at 25 °C the pyranose form dominates largely and especially in its *β*-form. These figures do not change significantly when the medium simulates Hadean waters (Dass et al., 2021).

Interestingly, Fig. 2 of (Dass et al., 2021) features break-even points of the eight curves. Beyond a temperature of ≈130 ^∘^C, the population is inverted with an increasing dominance of the furanose form, starting with a small excess of ≈1.5 × favoring the *β* form at the beginning, with a gap between the *α* and the *β* populations that widens as the temperature increases (whether in pure or Hadean water) reaching a *β*/*α* ratio of ≈2 at ≈130 °C (Dass et al., 2021). It is inferred, in conclusion, that the early hot atmosphere may have been the driver for the selection of the *β*-furanose form that remained to this day after the temperatures have cooled down.

This proclivity to select the *β*-furanose form can be enhanced at lower temperature in the presence of large temperature gradients as it occurs near hydrothermal vents for example. At room temperature, however, the data of Dass et al. show a slight but not substantial propensity for the *β*- over *α*-furanose, whether in pure aqueous solution or one that simulate Hadean medium (Dass et al., 2021).

Azofra et al.'s (Azofra et al., 2014) DFT exploration of the potential energy landscape generated thousands of rotamers of (deoxy)ribopyranose, (deoxy)ribofuranose in their open chain and *β*- and *α*-anomeric forms. In their study, these authors report results based on DFT vacuum-phase calculations with both the B3LYP/6-311++G(d,p) and M06-2X/6-311++G(d,p) chemical models (Azofra et al., 2014). The authors also find a dominance of the pyranose form over the furanose from 0 K to room temperature (298 K), in agreement with the more recent experimental results of Dass et al. (Dass et al., 2021). Meanwhile, within the small proportion of furanose, in the case of ribofuranose, the B3LYP/M06-2X functional predicts a higher Boltzmann populations of *α*-ribofuranose (3.4/0.2% (298 K) and 1.5/0.1% (0K)) than the *β*-ribofuranose (0.6/0.0% (298 K) and 0.2/0.0 (0K)). A similar trend is also found for the *α*-2′-deoxyribofuranose forms, in which case the respective Boltzmann populations (B3LYP/M06-2X) are (6.2/0.8% (298 K), 2.4/0.3% (0K)) compared with *β*-2′-deoxyribofuranose (1.2/0.1% (298 K) and 0.4/0.0 (0K)). One of the main results of Azofra et al.'s (Azofra et al., 2014) study is that the pyranose form is more populated than the furanose form at room (and lower) temperatures, but the results using both DFT functionals indicate a slight advantage, within the furanose population, for the *α*-anomer. However, the differences in energies and their corresponding Boltzmann's populations are probably within the error bars of the DFT calculations. Hence, we may conclude that these studies do not indicate a clear advantage of one furanose anomer over the other at room temperature.

Cocinero et al. performed a combined experimental/computational study (rotational FT-MW spectroscopy and three levels of theory: MP2/6-311++G(d,p), B3LYP//6-311++G(d,p), and M06-2X//6-311++G(d,p)) that again shows the almost exclusive dominance of the pyranose form in the gas-phase at room temperature (Cocinero et al., 2011). However, these authors also demonstrate that aqueous solvation increases the propensity for the furanose form at room temperature compared to the gas-phase (Cocinero et al., 2011).

Our results listed in Table 1 are consistent with the findings of Azofra et al.'s (Azofra et al., 2014) suggesting a borderline advantage of the *α*- over the *β*-furanose anomer at room temperature, whether in solvent or in the vacuum phase, and for either ribose or deoxyribose (see first three entries of Table 1, especially the third, for differences in Gibbs energy which are less than *ca*. 3 kcal/mol). In fact these results are consistent with all those mentioned above by other workers since they indicate an inconclusive advantage of one form or another.

**Table 1:** Differences in energies between the most stable *β*- and *α*-anomers for the sugars 2′-deoxy (d) or (r)ibose in vacuum and in aqueous environment in kcal/mol (equation (1)). Included differences are between: The total energies without (Δ*E*_*βα*_) and with zero-point vibrational correction (ZPE) (Δ*E*_*βα*(ZPE)_), and Gibbs energies $\Delta G_{\beta \alpha }^{0}$ at STP conditions. The listed results are from DFT (B3LYP/6-31G(*d*,*p*)) calculations. The integral equation formalism of the polarizable continuum model (IEFPCM) of solvation has been used to generate the results incorporating aqueous solvation at the same level of DFT theory.

	DNA			RNA		
	S^(1)^	vac.^(2)^	solv.^(3)^	S^(1)^	vac.^(2)^	solv.^(3)^
Δ*E*_*βα*_	dr	1.1	4.1	r	2.8	3.8
Δ*E*_*βα*(ZPE)_		1.2	3.7		2.7	2.8
$\Delta G_{\beta \alpha }^{0}$		1.5	3.2		2.8	1.9
Δ*E*_*βα*_	drMP	−5.1	−2.4	rMP	−3.0	0.8
Δ*E*_*βα*(ZPE)_		−4.9	−2.5		−3.2	−0.3
$\Delta G_{\beta \alpha }^{0}$		−3.9	−1.1		−1.8	−0.8

^(1)^ S = unspecified sugar or 5′-monophosphate (MP) sugar. ^(2)^ Differences in Δ*E*_*βα*_, Δ*E*_*βα*(ZPE)_, and in $\Delta G_{\beta \alpha }^{0}$ in vacuum at the DFT level. ^(3)^ Differences in Δ*E*_*βα*_, Δ*E*_*βα*(ZPE)_, and in $\Delta G_{\beta \alpha }^{0}$ in solvent.

Table 1 also lists the effect of adding the phosphate group at position 5' of the sugar. *The phosphate group has a significant effect whereby the slight advantage of the α- over the β-forms of the free sugars is now reversed*. As can be seen from the entries in the Table, the sugar monophosphates are slightly more stable in the *β*-forms, whether in gas- or solution-phase and whether ribose or deoxyribose (differences in Gibbs energy are all above a kcal/mol, approximately 2 - 3 kcal/mol for RNA and 4 - 5 kcal/mol in DNA, in vacuum, and with smaller magnitudes (but still negative values) in solution) (Table 1). (See Figs. S.1 – S.4 in the Supporting Information (SI).)

### 3.3 Which nucleoside anomer is more stable: *α* or *β*?

Ball-and-stick representations of the optimized geometries of all studied structures and their Gibbs energies of inter-conversion can be found in the SI (**Figs. S.5 – S.14**).

The 20 differences in energies between the *β*- and *α*-anomers for each of the nucleosides obtained from equation (1) are listed in Table 2. For most cases, the difference in stabilities of the anomers falls within the probable error bars of the theoretical method (DFT-B3LYP/6-31G(d,p)), that is, approximately 3 - 4 kcal/mol. In the case of DNA, all differences in Gibbs energies are <2 kcal/mol, whether in vacuum or in solution and for all five nucleosides. One notices that, in all cases, solvation magnifies the relative stability of the *α*-anomer by ≈2 kcal/mol. As for RNA in vacuum, Gibbs energies suggest a slight relative stability of the *β*-anomer of G over the *α*-form (by ≈2 kcal/mol), while the reverse is true for the rest, with the *α*-form being more stable by ≈2 kcal/mol for A, T, and U, and by ≈4 kcal/mol for C.

**Table 2:** Differences between the energies of the most stable *β*- and *α*-anomers of the 2′ deoxy (d) or ribonucleosides in vacuum and in aqueous environment (equation 1). Included differences are between: The total energies without (Δ*E*_*βα*_) and with zero-point vibrational correction (ZPE) (Δ*E*_*βα*(ZPE)_), and Gibbs energies $\Delta G_{\beta \alpha }^{0}$. All results are obtained from DFT (B3LYP/6-31G(*d*,*p*)) calculations. The integral equation formalism of the polarizable continuum model (IEFPCM) of solvation has been used to generate the results incorporating aqueous solvation at the same level of DFT theory.

	DNA			RNA		
	N^(1)^	vac.^(2)^	solv.^(3)^	N^(1)^	vac.^(2)^	solv.^(3)^
	*purines*					
Δ*E*_*βα*_	dA	1.2	0.6	A	1.7	3.9
Δ*E*_*βα*(ZPE)_		0.8	0.7		1.3	3.2
$\Delta G_{\beta \alpha }^{0}$		1.0	1.4		1.5	2.1
Δ*E*_*βα*_	dG	−0.9	0.3	G	−1.8	3.6
Δ*E*_*βα*(ZPE)_		−1.1	0.7		−1.9	3.1
$\Delta G_{\beta \alpha }^{0}$		−0.5	2.0		−1.5	2.0

	*pyrimidines*					
Δ*E*_*βα*_	dC	−1.4	2.4	C	4.9	−0.1
Δ*E*_*βα*(ZPE)_		−1.1	2.2		4.6	−0.5
$\Delta G_{\beta \alpha }^{0}$		−0.2	1.8		4.3	−0.8
Δ*E*_*βα*_	dT	−0.4	2.8	T	2.4	1.6
Δ*E*_*βα*(ZPE)_		−0.3	2.5		1.9	1.0
$\Delta G_{\beta \alpha }^{0}$		0.3	1.9		1.9	0.1
Δ*E*_*βα*_	dU	0.0	2.9	U	2.3	1.5
Δ*E*_*βα*(ZPE)_		0.1	2.6		1.8	1.2
$\Delta G_{\beta \alpha }^{0}$		0.7	2.0		1.8	0.8

^(1)^ N = unspecified nucleoside. ^(2)^ Differences in Δ*E*_*βα*_, Δ*E*_*βα*(ZPE)_, and in $\Delta G_{\beta \alpha }^{0}$ in vacuum. ^(3)^ Differences in Δ*E*_*βα*_, Δ*E*_*βα*(ZPE)_, and in $\Delta G_{\beta \alpha }^{0}$ in solvent.

In solution phase, and judging from the relative Δ*G* values, the *α*-forms of the purines are more stable than their *β* counterparts by ≈2 kcal/mol while for pyrimidines the differences between the two forms are negligible (below chemical accuracy of 1 kcal/mol), Table 2. These energy differences between the anomers are within an order of magnitude of the thermal energy at room temperature $k_{B}T$ (298 K) ≈0.5 kcal/mol, and that at 70 °C ($k_{B}T$ (343 K) ≈0.7 kcal/mol), a temperature beleived to have prevaled during the Archean eon when the first forms of primordial life may have emerged (Garcia et al., 2017).

The calculated energy differences listed in Table 2, whether Gibbs or total energies, fall within the probable error bars of the method and hence, while indicative, cannot be considered definitive. The consistency of the trend in Table 2 may allow us to conclude that there appears to be a small thermodynamic advantage for the *α*-anomer over the *β*-anomer in solution in both nucleic acids. In vacuum phase, in the case of DNA, the Gibbs energy differences are below chemical accuracy and hence the two forms are iso-energetic. Meanwhile, for RNA in vacuum generally the *α*-anomer is more stable (slightly for A, T, and U, and more notably for C) except for G for which the *β*-anomer has a slight advantage.

### 3.4 Which nucleotide anomer is more stable and in what conditions?

**Tables**3 and 4 give the relative (Gibbs) energies of the *α* and *β* anomers obtained *via* the “classical” pathway ((a + b) – Table 3, **Figs. S.15 – S.24**) and the alternative pathway ((c + d) – Table 4, **Figs. S.25 – S.34**). The sequences of the two-step additions defining the two pathways are represented in Fig. 2. The differences in energies were obtained by equations of the form of equation (1). Since all individual energies are negative, entries in these tables that are negative indicate that the *β* anomer is more stable and *vice versa*.

**Table 3:** Differences between the energies of the most stable *β*- and *α*-anomers of the 2′ deoxy (d) or (rib)onucleotides in vacuum and in aqueous environment (equation 1) as given by the reaction pathway sequence (a) and (b) of Fig. 2. Included differences are between: The total energies without (Δ*E*_*βα*_) and with zero-point vibrational correction (ZPE) (Δ*E*_*βα*(ZPE)_), and Gibbs energies $\Delta G_{\beta \alpha }^{0}$. All results are obtained from DFT (B3LYP/6-31G(*d*,*p*)) calculations. The integral equation formalism of the polarizable continuum model (IEFPCM) of solvation has been used to generate the results incorporating aqueous solvation at the same level of DFT theory.

	DNA			RNA		
	NMP^(1)^	vac.^(2)^	solv.^(3)^	NMP^(1)^	vac.^(2)^	solv.^(3)^
	*purines*					
Δ*E*_*βα*_	dAMP	−7.9	2.0	AMP	−8.0	−0.6
Δ*E*_*βα*(ZPE)_		−7.9	2.2		−8.9	−0.2
$\Delta G_{\beta \alpha }^{0}$		−7.7	2.4		−9.9	1.5
Δ*E*_*βα*_	dGMP	−18.6	1.8	GMP	−5.7	−2.8
Δ*E*_*βα*(ZPE)_		−18.7	1.1		−6.6	−2.3
$\Delta G_{\beta \alpha }^{0}$		−17.1	1.8		−6.7	−0.8

	*pyrimidines*					
Δ*E*_*βα*_	dCMP	4.7	−0.7	CMP	−8.0	−4.6
Δ*E*_*βα*(ZPE)_		4.2	−0.9		−8.6	−4.5
$\Delta G_{\beta \alpha }^{0}$		3.3	−0.6		−9.0	−3.2
Δ*E*_*βα*_	dTMP	5.9	−1.9	TMP	−1.5	−4.7
Δ*E*_*βα*(ZPE)_		6.0	−1.1		−2.2	−4.5
$\Delta G_{\beta \alpha }^{0}$		6.4	0.3		−2.5	−2.8
Δ*E*_*βα*_	dUMP	6.1	−1.3	UMP	−10.7	−4.4
Δ*E*_*βα*(ZPE)_		6.1	−1.3		−11.6	−4.0
$\Delta G_{\beta \alpha }^{0}$		6.6	−0.7		−12.5	−2.2

^(1)^ NMP = unspecified nucleoside 5′-monophosphate (nucleotide). ^(2)^ Differences in Δ*E*_*βα*_, Δ*E*_*βα*(ZPE)_, and in $\Delta G_{\beta \alpha }^{0}$ in vacuum at the DFT level. ^(3)^ Differences in Δ*E*_*βα*_, Δ*E*_*βα*(ZPE)_, and in $\Delta G_{\beta \alpha }^{0}$ in solvent.

**Table 4:** Differences between the energies of the most stable *β*- and *α*-anomers of the 2′ deoxy (d) or (rib)onucleotides in vacuum and in aqueous environment (equation 1) as given by the reaction pathway sequence (c) and (d) of Fig. 2. Included differences are between: The total energies without (Δ*E*_*βα*_) and with zero-point vibrational correction (ZPE) (Δ*E*_*βα*(ZPE)_), and Gibbs energies $\Delta G_{\beta \alpha }^{0}$. All results are obtained from DFT (B3LYP/6-31G(*d*,*p*)) calculations. The integral equation formalism of the polarizable continuum model (IEFPCM) of solvation has been used to generate the results incorporating aqueous solvation at the same level of DFT theory.

	DNA			RNA		
	NMP^(1)^	vac.^(2)^	solv.^(3)^	NMP^(1)^	vac.^(2)^	solv.^(3)^
	*purines*					
Δ*E*_*βα*_	dAMP	4.3	1.4	AMP	1.6	−0.5
Δ*E*_*βα*(ZPE)_		4.2	1.5		1.8	−0.8
$\Delta G_{\beta \alpha }^{0}$		4.3	1.6		2.6	−0.8
Δ*E*_*βα*_	dGMP	−3.5	−2.1	GMP	0.2	2.3
Δ*E*_*βα*(ZPE)_		−4.5	−1.6		0.0	1.0
$\Delta G_{\beta \alpha }^{0}$		−4.3	1.2		0.9	−0.2

	*pyrimidines*					
Δ*E*_*βα*_	dCMP	−12.6	0.2	CMP	0.0	0.7
Δ*E*_*βα*(ZPE)_		−12.4	0.1		−0.7	0.0
$\Delta G_{\beta \alpha }^{0}$		−12.8	0.7		−1.6	−0.3
Δ*E*_*βα*_	dTMP	−12.2	0.0	TMP	6.7	1.3
Δ*E*_*βα*(ZPE)_		−11.5	0.3		6.8	0.7
$\Delta G_{\beta \alpha }^{0}$		−10.6	1.7		7.5	0.1
Δ*E*_*βα*_	dUMP	−10.0	1.1	UMP	9.3	−1.1
Δ*E*_*βα*(ZPE)_		−10.1	1.1		9.9	−1.8
$\Delta G_{\beta \alpha }^{0}$		−10.9	1.3		11.1	−1.9

^(1)^ NMP = unspecified nucleoside 5′-monophosphate (nucleotide). ^(2)^ Differences in Δ*E*_*βα*_, Δ*E*_*βα*(ZPE)_, and in $\Delta G_{\beta \alpha }^{0}$ in vacuum at the DFT level. ^(3)^ Differences in Δ*E*_*βα*_, Δ*E*_*βα*(ZPE)_, and in $\Delta G_{\beta \alpha }^{0}$ in solvent.

Assuming RNA preceded DNA chronologically, a glance at Table 3 (nucleotides formed *via* pathway (a + b)) suggests that *β*-anomers are favored across the board in vacuum/non-polar medium. In this case, the relative Gibbs energies, ordered in decreasing magnitudes, are $\Delta G_{\beta \alpha }^{0}(\mathrm{UMP})\approx -13$ kcal/mol, $\Delta G_{\beta \alpha }^{0}(\mathrm{AMP})\approx -10$ kcal/mol, $\Delta G_{\beta \alpha }^{0}(\mathrm{CMP})\approx -9$ kcal/mol, $\Delta G_{\beta \alpha }^{0}(\mathrm{GMP})\approx -7$ kcal/mol, $\Delta G_{\beta \alpha }^{0}(\mathrm{TMP})\approx -3$ kcal/mol. Interestingly, the least remarkable difference is that of the rarely seen nucleotide, that is, TMP (favoring the *β*-form by only 3 kcal/mol) as opposed to the one that actually occur in today's RNA, that is, UMP which exhibits, in fact, the highest differential stability favoring the *β*-anomer (by 13 kcal/mol). Coincidence? There is no way to tell for certain, but suggestive it is.

Continuum solvation reduces the clear advantage of the *β*-anomer over their *α* counterparts to the level of computational noise, yet with consistency (except for a small reversal ≈2 kcal/mol for AMP) (Table 3). As emphasized above, the results including solvation can be taken as a qualitative indicator only given the lack of localized solvent-solute interaction(s) that may stabilize or destabilize the system.

As for DNA, the vacuum calculations suggest a considerable advantage for the canonical forms of purines and the reverse for pyrimidines. The advantage of the *β*-form is particularly marked in the case of dGMP by ≈17 kcal/mol of more stability (lower energy) compared to the *α*-form. In aqueous solution, the results are inconclusive being, probably, within computational uncertainties (as discussed above).

Moving to the path (c + d) (Fig. 2), Table 4 suggests that, in vacuum (or in non-polar medium), the two forms of the RNA nucleotides have indistinguishable stabilities within the probable error bars of the method except for TMP and UMP. In these latter two cases, the *α* form is considerably more stable with ≈8 and 11 kcal/mol, respectively. These observations are inconsistent with today's state of affairs on three grounds: (*i*) AMP and GMP are predicted to have energies marginally favoring their *α*-forms, and, more importantly (*ii*) TMP and UMP are much more stable in their *α*-form especially UMP. Since these contradict what is being observed – that pathway is less likely to have been Nature's choice leaving the other pathway (a + b) as a more probable scenario.

The addition of a phosphate group on the sugar first *then* the base last, in the (c + d) pathway, creates ample opportunity for the highly anionic oxygens of the phosphate to hydrogen bond with the sugar's hydroxyl groups (see **Figs. S.25 – S.34**). In the classical pathway, (a + b), when the base is added first on the sugar, the acidic hydrogens of the base in the *β* form – being in closer proximity to the phosphate group (both are on the same face of the sugar mean plane) – can form hydrogen bonds with the latter (see **Figs. S.15 – S.24**). This hydrogen bonding locks the phosphate group on that side of the mean plane of the sugar and competes with its capacity to form more hydrogen bonds with the sugar hydroxyl groups.

### 3.5 The order of addition of the three components of nucleotides matters

**Figure 3:**
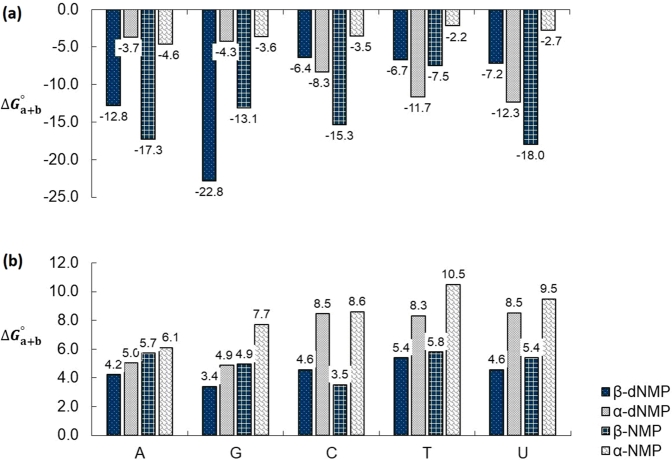
Comparison of Gibbs energies of reaction (Δ*G*^∘^) at 298^∘^K for the classic pathway (pathway (a + b), Fig. 2) leading to the 5 canonical *β*-nucleotides and their *α*-counterparts. The Gibbs energies of these reactions are defined by Eq. (2). **(a)** Δ*G*^∘^, B3LYP/6-31G (*d*,*p*) in vacuum, **(b)** Δ*G*^∘^, B3LYP/6-31G(*d*,*p*) in aqueous medium using the IEFPCM solvation model.

**Figure 4:**
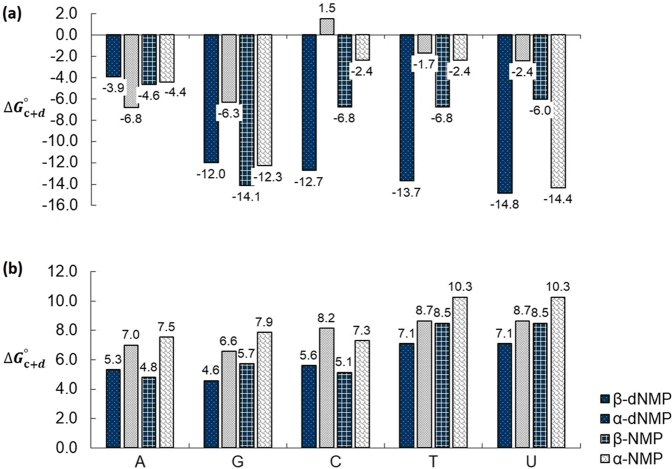
Comparison of Gibbs energies of reaction (Δ*G*^∘^) at 298^∘^K for the alternative pathway (pathway (c + d), Fig. 2) leading to the 5 canonical *β*-nucleotides and their *α*-counterparts. The Gibbs energies of these reactions are defined by Eq. (3). **(a)** Δ*G*^∘^, B3LYP/6-31G (*d*,*p*) in vacuum, **(b)** Δ*G*^∘^, B3LYP/6-31G(*d*,*p*) in aqueous medium using the IEFPCM solvation model.

The hypothesis being tested here is captured by the following question. Suppose a series of net reactions were available in prebiotic times that lead to the formation of the first nucleotides, whether those of DNA or of RNA. *Is there a particular order of addition that is more energetically favorable?* In other words, which one of the following net reactions, fleshed-out in Fig. 2, is energetically more favorable, *i.e.* leads to a more negative Δ*G*:

• *Reaction* (a+b):
  $$\begin{array}{c} \underline{\begin{array}{c} \mathrm{sugar}+\mathrm{base}\overset{\left(a\right)}{⟶}{\mathrm{Ns}}_{\left(a\right)}+H_{2}O\\ {\mathrm{Ns}}_{\left(a\right)}+H{}_{2}{\mathrm{PO}}_{4}^{-}\overset{\left(b\right)}{⟶}{\mathrm{Nt}}_{\left(a+b\right)}+H_{2}O \end{array}}\\ \mathrm{sugar}+\mathrm{base}+H_{2}{\mathrm{PO}}_{4}^{-}\overset{\left(a+b\right)}{⟶}{\mathrm{Nt}}_{\left(a+b\right)}+2H_{2}O,\;\Delta G_{\left(a+b\right)} \end{array}$$
• *Reaction* (c+d):
  $$\begin{array}{c} \underline{\begin{array}{c} \mathrm{sugar}+H_{2}{\mathrm{PO}}_{4}^{-}\overset{\left(c\right)}{⟶}5^{\prime }\text{-}{\mathrm{SMP}}_{\left(c\right)}+H_{2}O\\ 5^{\prime }\text{-}{\mathrm{SMP}}_{\left(c\right)}+\mathrm{base}\overset{\left(d\right)}{⟶}{\mathrm{Nt}}_{\left(c+d\right)}+H_{2}O \end{array}}\\ \mathrm{sugar}+\mathrm{base}+H_{2}{\mathrm{PO}}_{4}^{-}\overset{\left(c+d\right)}{⟶}{\mathrm{Nt}}_{\left(c+d\right)}+2H_{2}O,\;\Delta G_{\left(c+d\right)} \end{array}$$

where Ns = nucleoside and Nt = nucleotide in either the *α*- or the *β*-anomeric form, the sugar could be either ribose or deoxyribose, and 5′-SMP = 5′-sugar monophosphate. The subscripts in bracket indicate the addition sequence. There are a total of 2 (pathways) × 2 (sugars) × 5 (bases) × 2 (anomers) × 2 (solvation conditions) = 80 “reactions” in total the Gibbs energies of which are summarized in Table 5 (see also Figs. 3 and 4). The Gibbs energies of the two reaction pathways are defined by Eqs. (2) and (3). It is important to remind the reader that since the optimized geometry, and hence the energy, of the final product depends on the sequence, we have *different* “products” (local minima) (${\mathrm{Nt}}_{\left(a+b\right)}\neq {\mathrm{Nt}}_{\left(c+d\right)}$).

**Table 5:** Gibbs (Δ*G*^0^) energies at standard pressure and temperature in kcal/mol for a hypothetical condensation leading to the 5 canonical *β* ribonucleosides 5′-monophosphate (NMPs) (nucleotides) and their *α* counterparts in vacuum and in aqueous environment. The Gibbs energies of the two reaction pathways are defined by Eqs. (2) and (3). “Reaction” pathways are labeled according to Fig. 2. (From DFT calculations at the B3LYP/6-31G(*d*,*p*) level of theory, with aqueous solvation modeled with the IEFPCM model).

	***vacuum***							***solvent***						
	***classic path***													
	$\Delta G_{a}^{0}$		$\Delta G_{b}^{0}$		$\Delta G_{a+b}^{0}$		$\Delta \Delta G_{a+b}^{0}$^(2)^	$\Delta G_{a}^{0}$		$\Delta G_{b}^{0}$		$\Delta G_{a+b}^{0}$		$\Delta \Delta G_{a+b}^{0}$^(2)^
NMP^(1)^	*α*	*β*	*α*	*β*	*α*	*β*		*α*	*β*	*α*	*β*	*α*	*β*	
	**DNA**							**DNA**						
	*purines*							*purines*						
dAMP	2.5	2.1	−6.2	−14.9	−3.7	−12.8	−9.1	4.5	2.6	0.6	1.6	5.0	4.2	−0.8
dGMP	4.6	2.6	−8.8	−25.4	−4.3	−22.8	−18.6	4.3	3.0	0.6	0.4	4.9	3.4	−1.5
	*pyrimidines*							*pyrimidines*						
dCMP	6.5	4.8	−14.8	−11.3	−8.3	−6.4	1.9	5.9	4.5	2.6	0.1	8.5	4.6	−3.9
dTMP	8.1	6.9	−19.7	−13.7	−11.7	−6.7	4.9	6.3	4.9	2.1	0.5	8.3	5.4	−2.9
dUMP	7.8	7.0	−20.2	−14.2	−12.3	−7.2	5.2	6.3	5.0	2.2	−0.5	8.5	4.6	−4.0
	**RNA**							**RNA**						
	*purines*							*purines*						
AMP	2.3	1.0	−6.9	−18.3	−4.6	−17.3	−12.6	3.7	3.9	2.4	1.8	6.1	5.7	−0.4
GMP	6.2	1.9	−9.8	−15.1	−3.6	−13.1	−9.5	4.3	4.4	3.4	0.6	7.7	4.9	−2.8
	*pyrimidines*							*pyrimidines*						
CMP	1.9	3.4	−5.4	−18.8	−3.5	−15.3	−11.8	3.5	2.7	3.1	0.8	8.6	3.5	−5.1
TMP	6.7	5.7	−8.9	−13.2	−2.2	−7.5	−5.3	7.7	5.9	2.8	−0.1	10.5	5.8	−4.7
UMP	6.8	5.8	−9.5	−23.8	−2.7	−18.0	−15.2	6.9	5.8	2.6	−0.4	9.5	5.4	−4.1
	***alternative path***													
	$\Delta G_{c}^{0}$		$\Delta G_{d}^{0}$		$\Delta G_{c+d}^{0}$		$\Delta \Delta G_{c+d}^{0}$^(2)^	$\Delta G_{c}^{0}$		$\Delta G_{d}^{0}$		$\Delta G_{c+d}^{0}$		$\Delta \Delta G_{c+d}^{0}$^(2)^
NMP^(1)^	*α*	*β*	*α*	*β*	*α*	*β*		*α*	*β*	*α*	*β*	*α*	*β*	
	**DNA**							**DNA**						
	*purines*							*purines*						
dAMP	−3.1	−8.5	−3.7	4.6	−6.8	−3.9	2.9	2.0	−2.3	5.0	7.6	7.0	5.3	−1.7
dGMP	−3.1	−8.5	−3.2	−3.5	−6.3	−12.0	−5.7	2.0	−2.3	4.6	6.9	6.6	4.6	−2.0
	*pyrimidines*							*pyrimidines*						
dCMP	−3.1	−8.5	4.7	−4.2	1.5	−12.7	−14.3	2.0	−2.3	6.2	7.9	8.2	5.6	−2.5
dTMP	−3.1	−8.5	1.4	−5.2	−1.7	−13.7	−12.0	2.0	−2.3	6.7	9.4	8.7	7.1	−1.6
dUMP	−3.1	−8.5	0.7	−6.3	−2.4	−14.8	−12.4	2.0	−2.3	6.4	8.7	8.4	6.4	−1.9
	**RNA**							**RNA**						
	*purines*							*purines*						
AMP	−5.2	−9.8	0.8	5.1	−4.4	−4.6	−0.2	1.8	−0.9	5.7	5.7	7.5	4.8	−2.8
GMP	−5.2	−9.8	−7.1	−4.4	−12.3	−14.1	−1.8	1.8	−0.9	6.0	6.6	7.9	5.7	−2.1
	*pyrimidines*							*pyrimidines*						
CMP	−5.2	−9.8	2.8	3.0	−2.4	−6.8	−4.4	1.8	−0.9	5.5	6.0	7.3	5.1	−2.2
TMP	−5.2	−9.8	−8.4	1.0	−13.6	−8.8	4.8	1.8	−0.9	8.4	9.4	10.3	8.5	−1.8
UMP	−5.2	−9.8	−9.2	3.7	−14.4	−6.0	8.4	1.8	−0.9	8.1	7.0	9.9	6.1	−3.8

^(1)^ NMP = unspecified nucleoside 5′-monophosphate (nucleotide). ^(2)^ The ΔΔ values are the Gibbs energies of reaction along a given two-step pathway for the *β* anomer minus the same pathway but for the *α* anomer. For example, from the pathways labeled in Fig. 2, $\Delta \Delta G_{\mathrm{Rx}(a+b)}^{0}$ is the difference of ${\left(\Delta G_{a+b}^{0}\right)}_{\beta }-{\left(\Delta G_{a+b}^{0}\right)}_{\alpha }$. In turn, ${\left(\Delta G_{a+b}^{0}\right)}_{\beta }$, for instance, is the sum of the Gibbs energies for the condensation reaction along the pathway (a) then (b) yielding the nucleotide. Expressed symbolically, ${\left(\Delta G_{a+b}^{0}\right)}_{\beta }={\left(\Delta G_{a}^{0}\right)}_{\beta }+{\left(\Delta G_{b}^{0}\right)}_{\beta }$. Hence, in general: $\Delta \Delta G_{i+j}^{0}={\left(\Delta G_{i+j}^{0}\right)}_{\beta }-{\left(\Delta G_{i+j}^{0}\right)}_{\alpha }$, where *i* = a, c, and *j* = b, d.

A glance at Table 5 suggests that the pathway (a + b) for the *β*-anomer is the most favored (more exergonic) in vacuum yet both pathways are endergonic in the continuum solvation model used. Hence the following discussion will focus on pathway (a + b) with the vacuum-phase results examined first. As can be seen from Table 5 and Fig. 3, the condensation reaction between a base and a sugar (reaction (a)) is not favored thermodynamically either in vacuum or aqueous solution. However, the next coupled step in this pathway, step (b), is sufficiently exergonic to drive the entire reaction to competition with negative free energy falling in magnitude within the range 11 kcal/mol <|ΔG|<24 kcal/mol in the case of the *β*-anomers, and to a lesser extent in the case of the *α*-anomers in which case the magnitudes of the energies of reaction are 6 kcal/mol <|ΔG|<20 kcal/mol.

It is further noticed from Table 5 that step (b) reactions are generally more exergonic for the *β*-anomers of RNA than for their DNA counterparts. This second step, (b), is also exergonic for the *α*-anomers. This step, for most *α*-nucleotides, is *less* exergonic (and significantly so) than the corresponding *β*-nucleotides except for the deoxyribonucleotides of the pyrimidine bases.

The overall reaction energies strongly favor the (a + b) pathways for *all* ribonucleotides in vacuum and for both anomers. The overall *β*-pathways are typically doubly or triply more exergonic than the *α*-pathways (as can be seen from Table 5 and Fig. 3) except in the case of the pyrimidines-deoxy-ribonucleotides. The differential Gibbs energies between the *α*- and *β*-pathways, ΔΔ*G*_(a + b)_, that captures the effect of α/β isomerization on the overall reaction Gibbs energies are more exergonic for the *β*-reactions except for the pyrimidines in DNA.

In aqueous medium, an examination at the overall energies of the (a + b) pathways shows that solvation flips all the vacuum-phase spontaneous reactions to non-spontaneous ones (Table 5 and Fig. 3). This is in line with the “water problem” (Joyce and Orgel, 1999) which seems to support a non-polar primordial soup. Interestingly, the reactions of the *α*-anomers are *all* more endergonic than those with the *β*-reactions, again suggesting that – in this case – the *β*-reaction is “less forbidden”, so to speak, than the *α*-counterpart.

In vacuum, for the alternative (c + d) pathway, the first step, namely (c), the condensation of the phosphate and the sugar is exergonic across the board, especially for the *β*-form (Table 5, and Fig. 4). Meanwhile when the sugar is substituted at its 5′ position by the phosphate group, the energies of step (d) do not suggest particularly strong trends. The overall reaction energy, though, still indicates that all are spontaneous but to lesser extents than the classical pathway (as mentioned above). Continuum solvation generally flattens the magnitudes of all reactions in the alternative pathways. In this case, step (c) is converted to a non-spontaneous reaction for the *α*-anomers and marginally exergonic for all the *β*-anomers. The next step (d) in water is unfavorable in all cases leading to an overall endergonic (c + d) pathway in all cases (as in the classical pathway), with – on average - a marginally less favorable reaction in aqueous medium for both anomers.

It is concluded that *the classical pathway for the β*-*anomers* (*the anomer which prevails in today's nucleic acids*) *emerges, again, as the generally favored thermodynamic choice*.

### 3.6 Sugar exchange reactions between U and T nucleosides and nucleotides

Lesk posed the question of “*Why does DNA contain Thymine and RNA Uracil?*” in the title an important paper that appeared as early as 1969 (Lesk, 1969). Lesk suggested several reasons for the choice including the suggestion of a slight thermodynamic advantage of the dominant forms over the minor forms. This point completes the present study which is addressed in a similar fashion as the manner as above.

**Figure 5:**
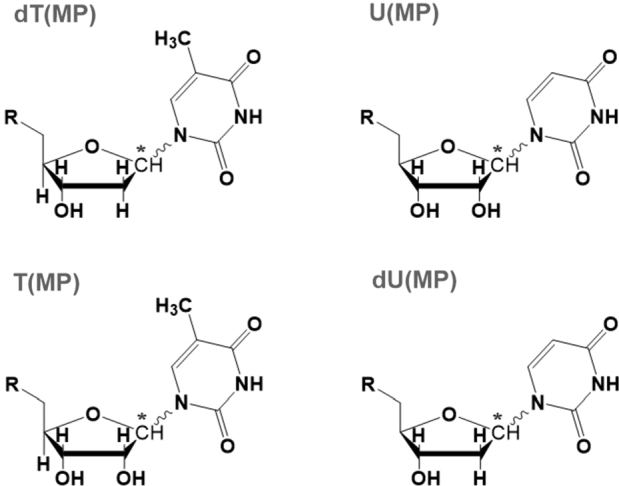
Natural and un-natural nucleosides. **(*Top*)** the predominant form occurring in nature, that is, 2′-deoxythymidine (dT) and uridine (U) occurring in DNA and RNA respectively. **(*Bottom*)** The minor forms, *i.e.*, thymidine (T) and 2′-deoxyuridine (dU) which are not normally incorporated in RNA and DNA, respectively. The sugar exchange (or swapping) is written as “chemical reactions” in Eqs. (4) and (5). Also see text and Table 6. (The star (⁎) denotes the anomeric center (C1′) of the sugar.)

From two bases (U, T), two sugars (ribose and 2′-deoxyribose), and two configurations (*α*, *β*) there are 8 possibilities (see Fig. 5). The top panel of this figure represents the (canonical) nucleosides that predominate in the genetic material of all contemporary living organisms, that is to say, thymine on deoxyribose (dT) and uracil on ribose (U). The bottom panel of Fig. 5 presents those that occur infrequently in the genetic material, *i.e.*, T and dU.

Table 6 compares the calculated energies of the thiamine and uracil nucleosides in hypothetical sugar exchange reactions, defined by “chemical reactions” (reaction 4) and (reaction 5) below, (where, for clarity, “r” is added to denote ribose sugar):

(4) β-rT+β-dU⟶β-dT+β-rU,

and

(5) α-rT+α-dU⟶α-dT+α-rU,

in both the vacuum phase and solvent phase.

**Table 6:** Differences between the energies of the canonical (predominant) nucleosides and their minor counterparts (Fig. 5) in vacuum and in aqueous environment (energies of the canonical form minus that of the minor form). Included differences are between: The total energies without (Δ*E*) and with zero-point vibrational correction (ZPE) (Δ*E*_(ZPE)_), and Gibbs energies Δ*G*^∘^. All energies are in kcal/mol and are obtained from DFT (B3LYP/6-31G(*d*,*p*)) calculations. The sugar exchange (or swapping) is written as “chemical reactions” in Eqs. (4) and (5). The integral equation formalism of the polarizable continuum model (IEFPCM) of solvation has been used to generate the results incorporating aqueous solvation at the same level of DFT theory.

Compared systems^(1)^	vac.			solv.		
	Δ*E*	Δ*E*_(ZPE)_	Δ*G*^∘^	Δ*E*	Δ*E*_(ZPE)_	Δ*G*^∘^
Δ*X*(*β*dT + *β*U-*β*T-*β*dU)	−0.0	−0.0	−0.0	0.3	0.1	−0.3
Δ*X*(*α*dT + *α*U-*α*T-*α*dU)	0.4	0.4	0.4	0.5	0.0	−0.8
Δ*X*_a+b_(*β*dTMP + *β*UMP-*β*TMP-*β*dUMP)	−9.3	−9.5	−10.0	0.01	0.5	0.4
Δ*X*_a+b_(*α*dTMP + *α*UMP-*α*TMP-*α*dUMP)	0.1	0.1	0.1	0.3	−0.2	−1.2
Δ*X*_c+d_(*β*dTMP + *β*UMP-*β*TMP-*β*dUMP)	0.3	1.7	3.9	−3.9	−3.5	−1.7
Δ*X*_c+d_(*α*dTMP + *α*UMP-*α*TMP-*α*dUMP)	0.0	0.0	−0.1	−0.5	−0.2	−0.1

^(1)^ΔX=ΔE, Δ*E*_(ZPE)_, $\Delta G^{0}$.

These reactions switch the pair of bases from their sugars in their canonical nucleosides to the sugars in their non-canonical ones delivering the energies of sugar double exchange “reactions”. The energies listed in Table 6 indicate a consistent lack of any significant energy difference upon affecting this transformation in all types of calculations and energies in the cases of the nucleosides (the first two rows in Table 6). Thus, for the nucleosides, all differences in the Table (whether of uncorrected total energy differences, ZPE-corrected energy differences, or Gibbs energy differences are below chemical accuracy) suggest essentially equal stability of the “correct” and “wrong” nucleosides. *Thus, there is no clear thermodynamic advantage of attaching one base on one particular sugar, whether in the α- or in the β-configurations, in the vacuum or solution phase*.

Next, the effect of attaching a phosphate group at position 5′ on the relative stabilities of the canonical and non-canonical nucleotides is explored. As mentioned above, we have two distinct step-wise additions of the three components of the nucleotides as illustrated in Fig. 2. The energies of the “sugar exchange reactions” for both pathways can be found in Table 6. From the listed entries in this Table, the most remarkable one is that of the exchange of the sugar in the *β*-forms in vacuum (or non-polar medium), clearly favoring the canonical form by up to 10 kcal/mol along the (a + b) pathway (adding the base first then the phosphate). This again reinforces our suggestion that the classical pathway is favored and the thermodynamic advantage of the *β*-anomeric form existing in contemporary nucleic acids. This advantage of the 10 kcal/mol of the “correct” over the “wrong” pairing of base with sugar is reduced to noise below chemical accuracy in aqueous solution.

The alternative pathway (c + d) (phosphate first then base) leads to a reversal of the energetic advantage of the *β*-forms in vacuum in favor of minor form by ≈4 kcal/mol in terms of Δ*G*. In aqueous solution, the (c + d) pathway slightly favors the canonical forms by ΔG≈2 kcal/mol. See Table 6.

From these considerations it may be inferred that only when the phosphate is among the “reactants” there exists an advantage of the canonical forms: (*i*) by ≈14 kcal/mol given the order of addition is base first then phosphate in a non-polar medium, or (*ii*) by ≈2 kcal/mol in aqueous environment for the reverse order of addition, that is, phosphate first.

## 4 Conclusions

The calculations suggest a slight thermodynamic advantage that favors the selection of the *β*- over the *α*-anomers. This is aligned with the concept of an evolutionary “energetic” selection of the fittest. Calculations accounting for implicit solvation in aqueous medium renders either pathways (a + b) or (c + d) thermodynamically unfavorable (Figs. 3 and 4). This last observation is consistent with the well-known “water problem”.

The present work suggests an order of combination of the three nucleotide components: The condensation of the base with the sugar is *first* followed by the condensation of the phosphate at the 5′, second. That is, the “classical pathway” emerges as the natural choice for the sequential addition of these components of nucleotides. As mentioned already, the addition of the two first reactants changes the geometries sufficiently to result in different geometries (and energies) when the third reactant is then added as the last condensation.

The final question addressed in this work is whether Nature's choice of incorporating U in RNA and T in DNA is consistent with a thermodynamic explanation. The results suggest an affirmative answer. Indeed, a comparison of the canonical *vs*. the non-canonical nucleotides of these two bases, (*i*) reinforces the conclusion that the classical pathway is favored, and (*ii*) indicates an advantage of the canonical pairs compared to the no-canonical pairs when gauged by the “sugar exchange reactions”. Furthermore, this thermodynamic advantage exists only for the *β*-anomers (10 kcal/mol) and vanishes in the case of the *α*-anomers.

Thermodynamics have been invoked as a driver behind the syntax of the genetic code as seen today (Grosjean and Westhof, 2016; Klump et al., 2020). Three decades ago, an editorial in *Nature* had the intriguing title “[*i*]*s Darwinism a thermodynamic necessity*?” (Maddox, 1991). That editorial highlights a paper by Torres in which Darwinian “*fitness*” has been formulated in thermodynamic terms (Torres, 1991). Torres addresses the logical fallacy of *circulus in probando* (circle in proving, commonly known as circular reasoning) of the concept of *survival of the fittest* (Torres, 1991). The fallacy is centered on that fittest is, *by definition*, the ability to be a survivor (Torres, 1991). These earlier works provide the context of the present one underscoring thermodynamics' role in driving the natural selection of today's canonical nucleotides.

This research addresses the question of why Nature selected the building blocks of nucleic acids as we know them today? The answer is sought in thermodynamic terms, with the underlying hypothesis that free energies are a factor that may have driven particular evolutionary choices. Other factors may have contributed at a particular selection of a molecular form. Such factors may include, for example, kinetics, catalysis including self-catalysis, interaction with light, *etc*. Our purpose here is much more modest and restricted as the questions addressed suggest.

Other factors that were not considered in this work is the effect of the inclusion of ions as well as of explicit solvation on the energy ordering. For now, the question this paper is addressing is, again, more modest and, that is, in the absence of these additional and relevant factors, *what is the energy ordering of the nucleotides in their isolated forms with and without continuum solvation*?

## Declarations

### Author contribution statement

Lázaro A. M. Castanedo, Chérif F. Matta: Conceived and designed the experiments; Performed the experiments; Analyzed and interpreted the data; Contributed reagents, materials, analysis tools or data; Wrote the paper.

### Funding statement

Professor Chérif Farid Matta was supported by Funding 10.13039/501100002790Canadian Network for Research and Innovation in Machining Technology, Natural Sciences and Engineering Research Council of Canada (NSERC DG-2015), and by 10.13039/501100000196CFI (CFI-Leaders Opportunity Fund 2020).

### Data availability statement

Data associated with this study has been deposited at Mendeley Data under Castanedo, Lázaro A. M.; Matta, Chérif F. (2022), “On the Prebiotic Selection of Nucleotide Anomers: Computational Data”, Mendeley Data, V2, https://data.mendeley.com/datasets/khxvtshbs2/2.

### Declaration of interests statement

The authors declare no conflict of interest.

### Additional information

Supplementary content related to this article has been published online at https://doi.org/10.1016/j.heliyon.2022.e09657.

No additional information is available for this paper.

## Supplementary material

The following Supplementary material is associated with this article:

On the Prebiotic Selection of Nucleotide Anomers - SI.doc Ball-and-stick representations of the optimized geometries (B3LYP/6-31G(*d*,*p*)), in vacuum and in solvent (IEFPCM continuum solvation model), along with the Gibbs energies of the elementary transformations considered in this work. On the Prebiotic Selection of Nucleotide Anomers - SI.doc
