# Supplementary material for: On the prebiotic selection of nucleotide anomers: A computational study
Source: Heliyon. 2022 Jun 9;8(6):e09657. doi: 10.1016/j.heliyon.2022.e09657 (PMC9243047; doi:10.1016/j.heliyon.2022.e09657)
Supplement: On the Prebiotic Selection of Nucleotide Anomers - SI.doc — Ball-and-stick representations of the optimized geometries (B3LYP/6-31G(d,p)), in vacuum and in solvent (IEFPCM continuum solvation model), along with the Gibbs energies of the elementary transformations considered in this work. [file mmc1.doc]

**On the Prebiotic Selection of Nucleotide Anomers: A Computational Study**

Lázaro A. M. Castanedo,^(1,2)^ Chérif F. Matta^(1-4)*^

*^(1)^ Department of Chemistry, Saint Mary's University, Halifax, Nova Scotia, Canada B3H 3C3. ^(2)^ Department of Chemistry and Physics, Mount Saint Vincent University, Halifax, Nova Scotia, Canada B3M 2J6. ^(3)^ Department of Chemistry, Dalhousie University, Halifax, Nova Scotia, Canada B3H 4J3. ^(4)^ Dép. de chimie, Université Laval, Québec, Québec, Canada G1V 0A6*

*.*

*** Corresponding Author: [cherif.matta@msvu.ca](mailto:cherif.matta@msvu.ca)

**SUPPORTING INFORMATION**

Ball-and-stick representations of the optimized geometries (B3LYP/6-31G(*d*, *p*)), in vacuum and in solvent (IEFPCM continuum solvation model), along with the Gibbs energies of the elementary transformations considered in this work.


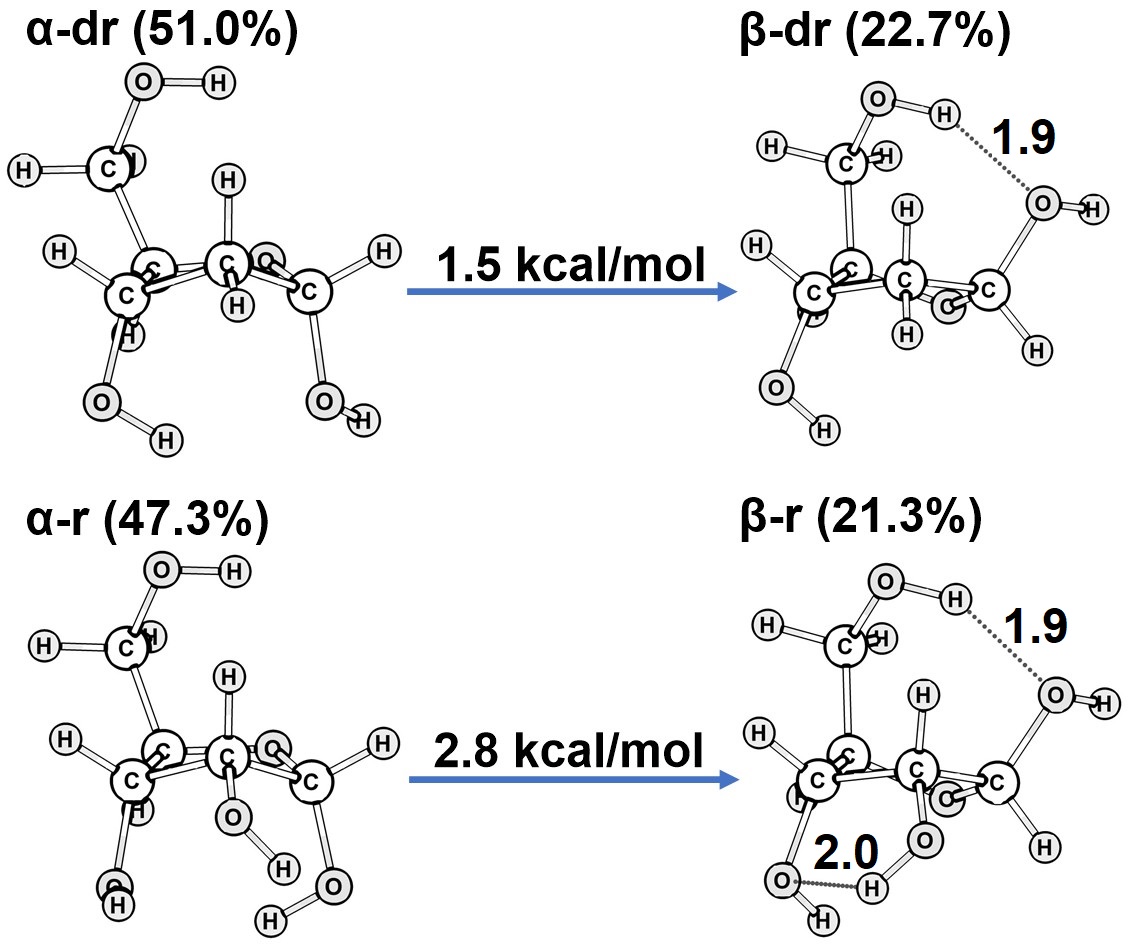


**S. 1** Display of the optimized geometries with bond lengths (in ångströms (Å)) for the studied β- and α-2'-deoxy (d) and (r)ibose in vacuum. (***Top****)* D-2'-deoxyribose (dr). **(*Bottom)*** D-ribose (r). The energy quoted in kcal/mol is the Gibbs energy of the β-form minus the Gibbs energy of the α-form (**Eqn (1)**) obtained at the DFT-B3LYP/6-31G(*d*,*p*). See text and **Table 1**. (X%) represents the relative population in % for each conformer obtained at the PM7 level.


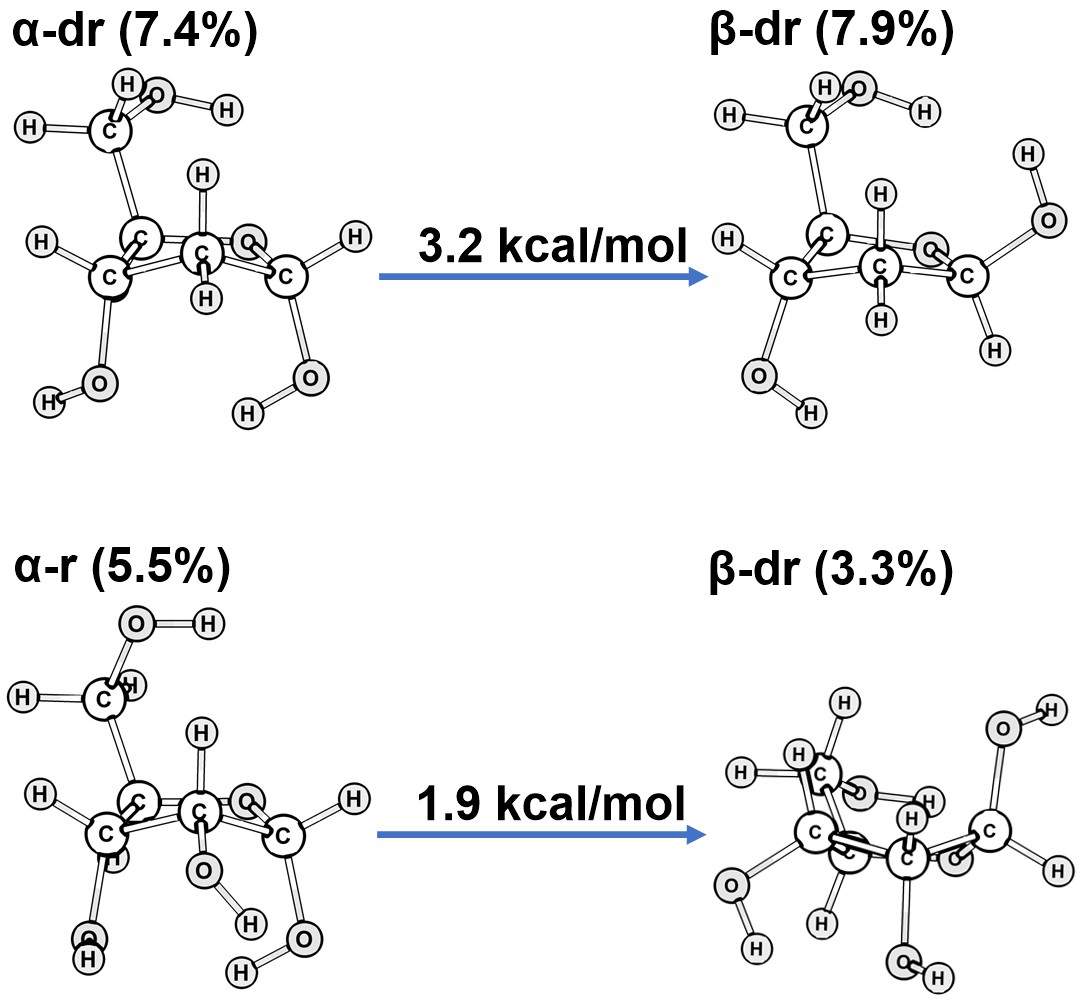


**S. 2** Display of the optimized geometries with bond lengths (in ångströms (Å)) for the studied β- and α-2'-deoxy (d) and (r)ibose obtained using the IEFPCM model for the aqueous solvation. (***Top****)* D-2'-deoxyribose (dr). **(*Bottom)*** D-ribose (r). The energy quoted in kcal/mol is the Gibbs energy of the β-form minus the Gibbs energy of the α-form (**Eqn (1)**) obtained at the DFT-B3LYP/6-31G(*d*,*p*). See text and **Table 1**. (X%) represents the relative population in % for each conformer obtained at the PM7 level.


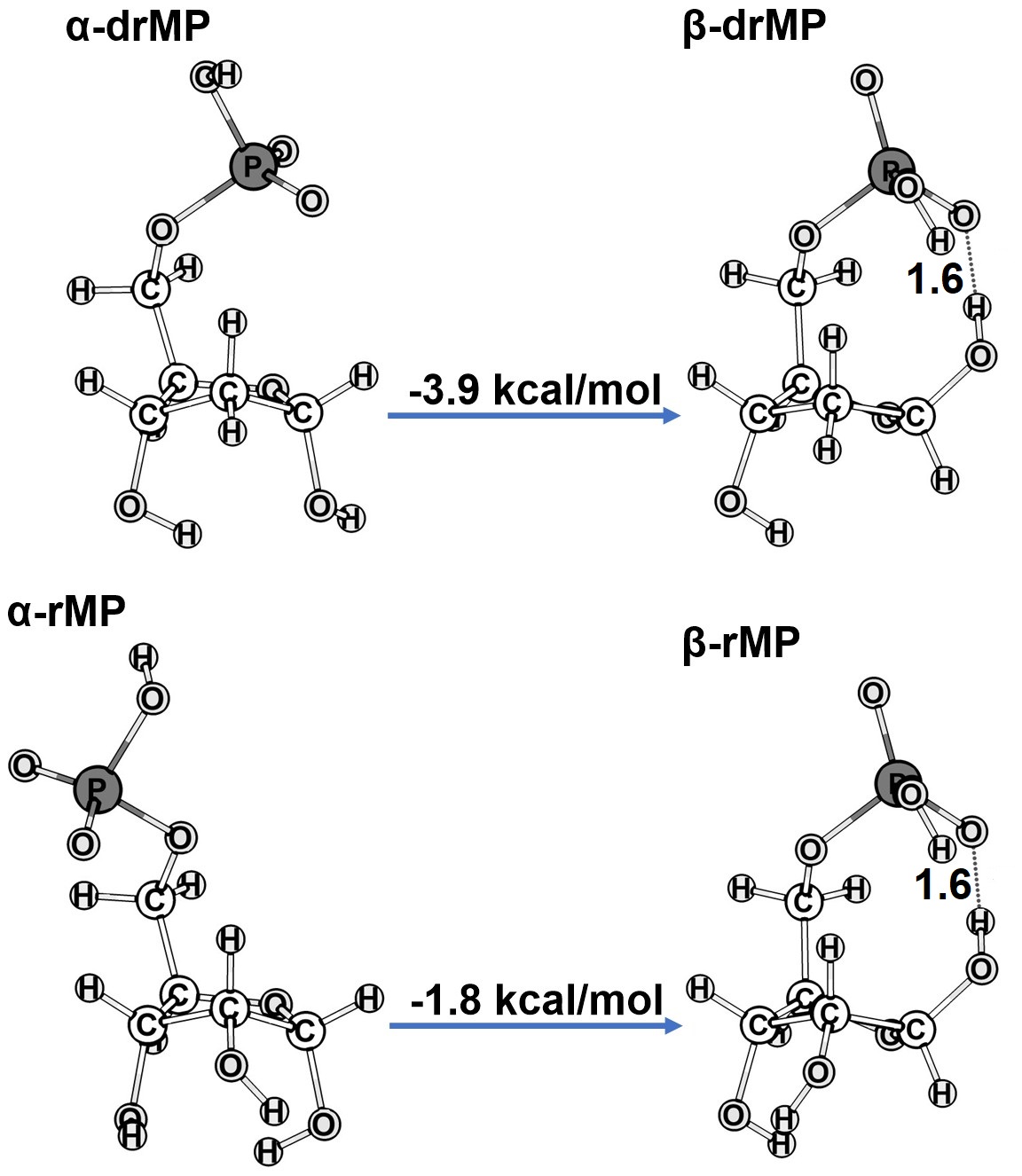


**S. 3** Display of the optimized geometries with bond lengths (in ångströms (Å)) for the studied β- and α- 5'-monophosphate(MP) sugar in vacuum. (***Top****)* D-2'-deoxyribose-5'-monophosphate (drMP). **(*Bottom)*** D-ribose-5'-monophosphate (rMP). The energy quoted in kcal/mol is the Gibbs energy of the β-form minus the Gibbs energy of the α-form (**Eqn (1)**) obtained at the DFT-B3LYP/6-31G(*d*,*p*). See text and **Table 1**.


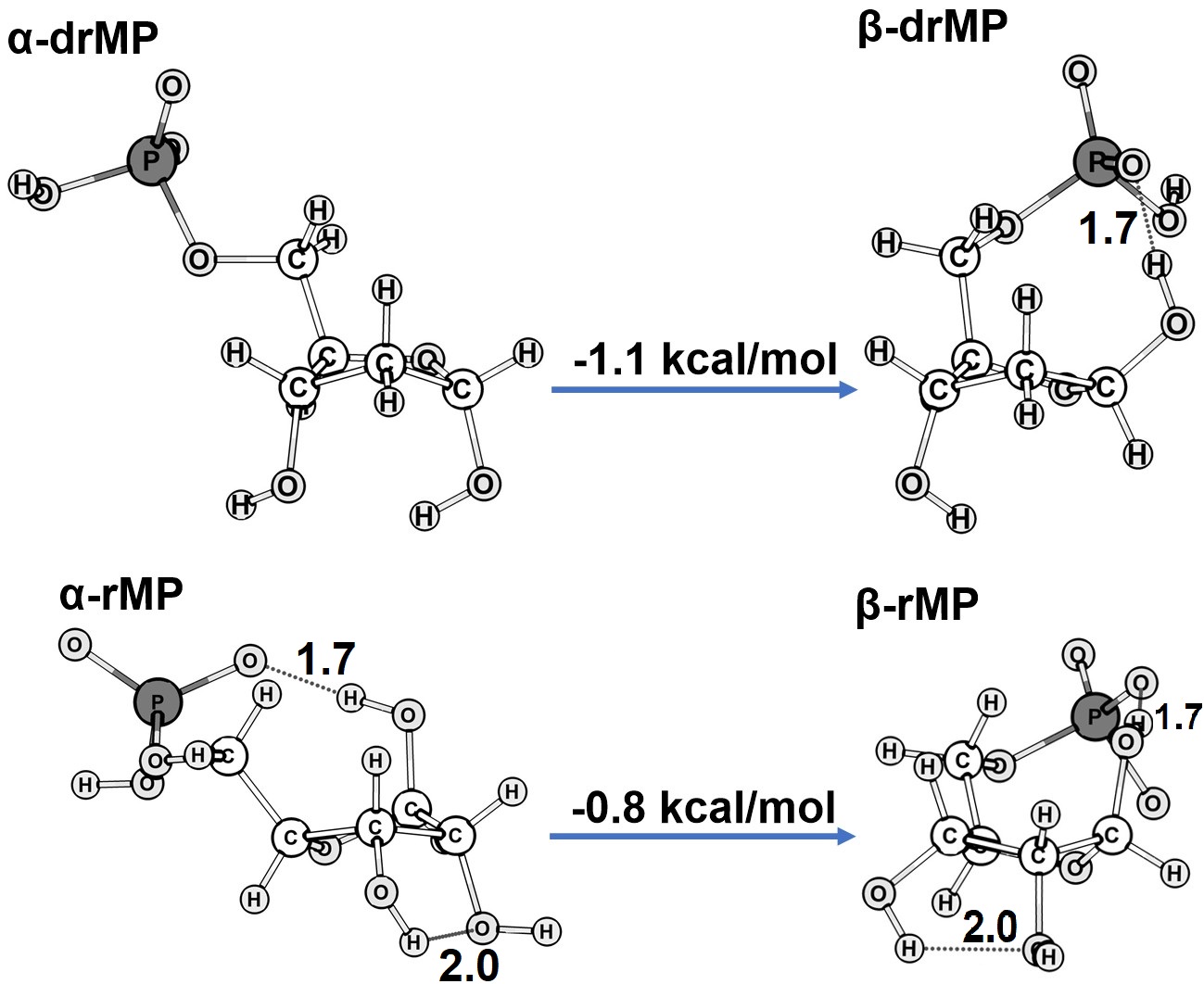


**S. 4** Display of the optimized geometries with bond lengths (in ångströms (Å)) for the studied β- and α- 5'-monophosphate(MP) sugar obtained using the IEFPCM model for the aqueous solvation. (***Top****)* D-2'-deoxyribose-5'-monophosphate (drMP). **(*Bottom)*** D-ribose-5'-monophosphate (rMP). The energy quoted in kcal/mol is the Gibbs energy of the β-form minus the Gibbs energy of the α-form (**Eqn (1)**) obtained at the DFT-B3LYP/6-31G(*d*,*p*). See text and **Table 1**.


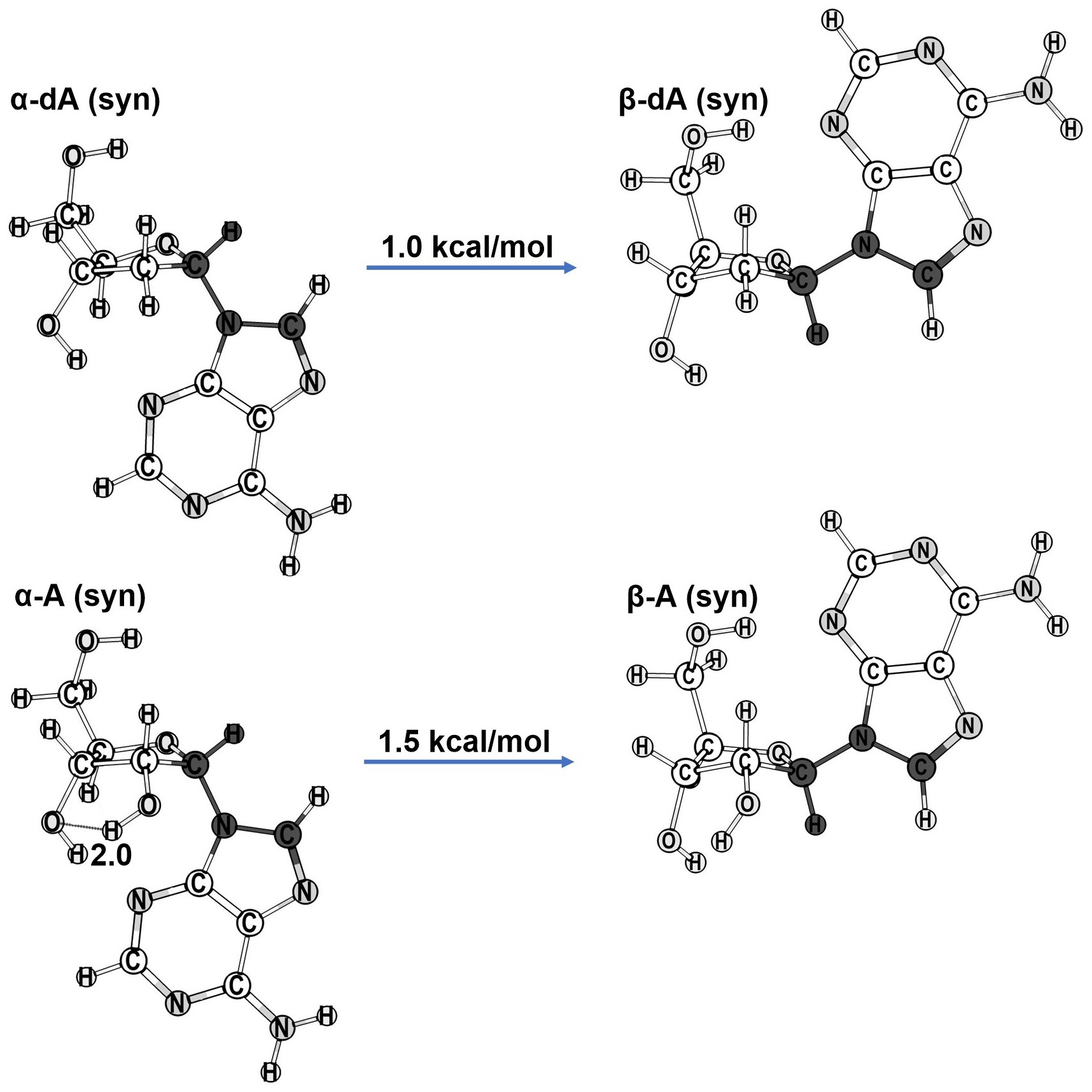


**S. 5** Display of the optimized geometries with bond lengths (in ångströms (Å)) for the studied β- and α-nucleosides of adenine (A) in vacuum. (***Top****)* 2'-deoxyadenosine (dA). **(*Bottom)*** Adenosine (A). The energy quoted in kcal/mol is the Gibbs energy of the β-form minus the Gibbs energy of the α-form (**Eqn (1)**) obtained at the DFT-B3LYP/6-31G(*d*,*p*). See text and **Table 2.**  The atoms involved in the torsion angle rotated in the PES are in bold.


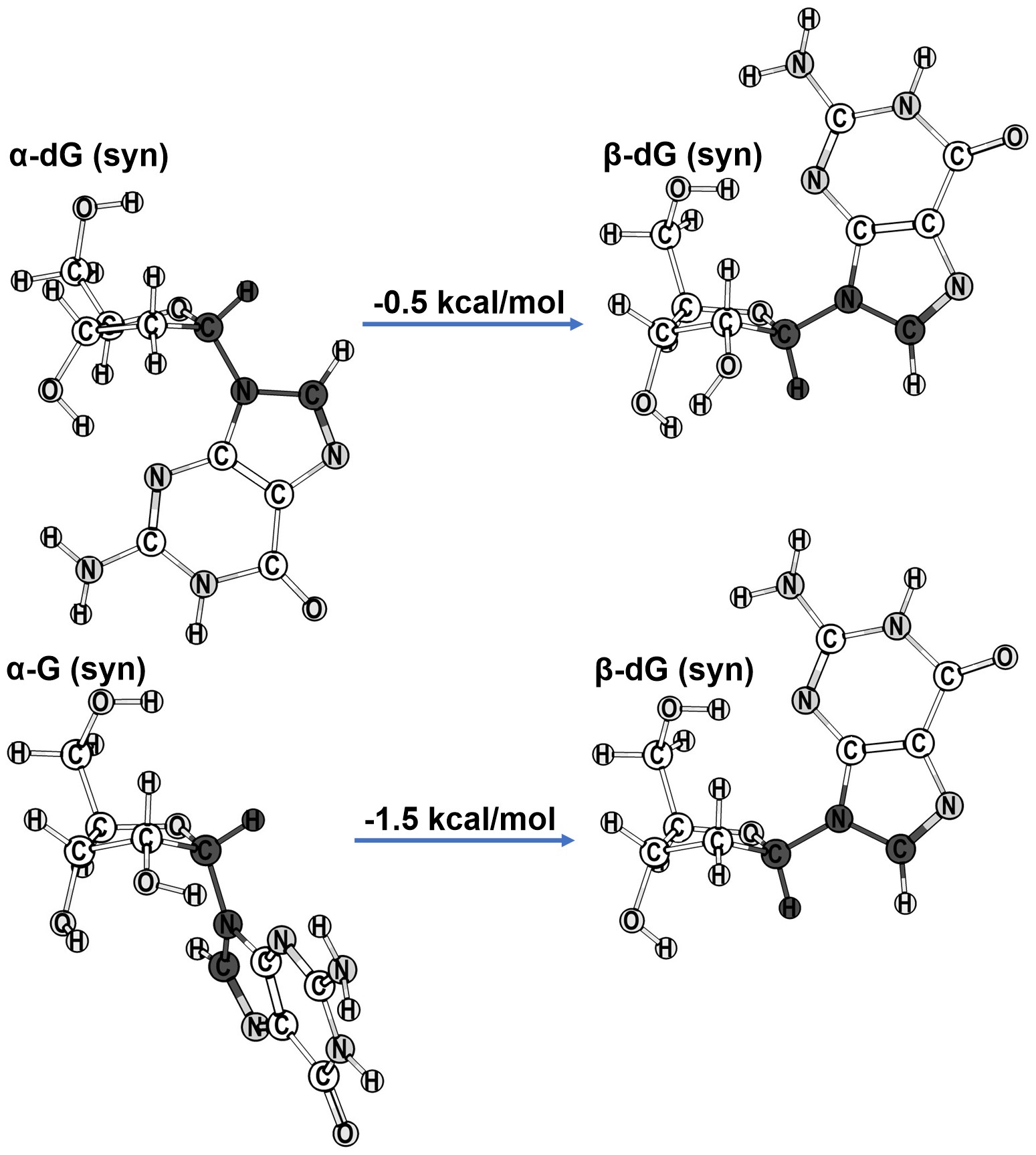


**S. 6** Display of the optimized geometries with bond lengths (in ångströms (Å)) for the studied β- and α-nucleosides of guanine (G) in vacuum. (***Top****)* 2'-deoxyguanosine (dG). **(*Bottom)*** Guanosine (G). The energy quoted in kcal/mol is the Gibbs energy of the β-form minus the Gibbs energy of the α-form (**Eqn (1)**) obtained at the DFT-B3LYP/6-31G(*d*,*p*). See text and **Table 2.**  The atoms involved in the torsion angle rotated in the PES are in bold.


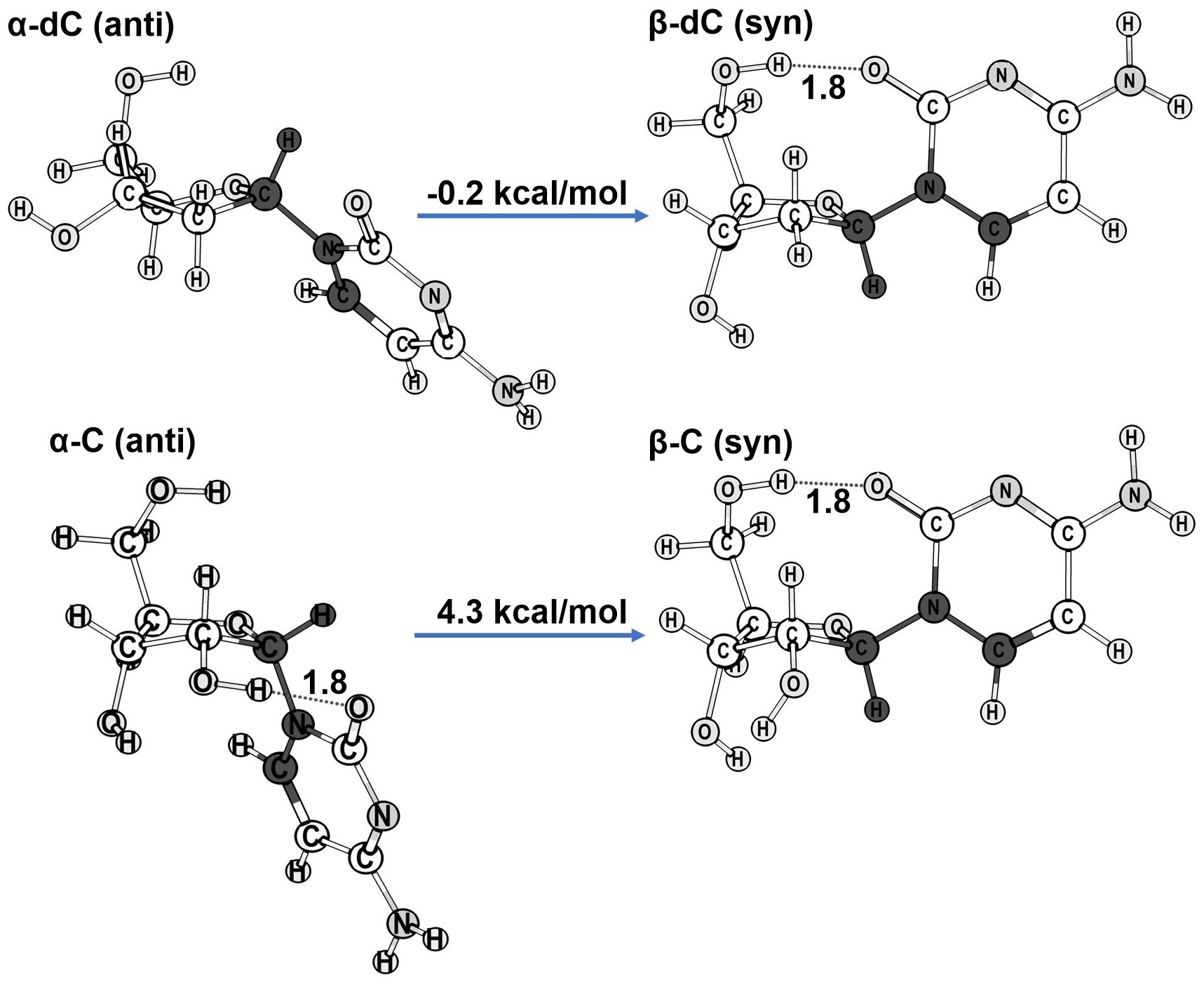


**S. 7** Display of the optimized geometries with bond lengths (in ångströms (Å)) for the studied β- and α-nucleosides of citosine (C) in vacuum. (***Top****)* 2'-deoxycytidine (dC). **(*Bottom)*** Cytidine (C). The energy quoted in kcal/mol is the Gibbs energy of the β-form minus the Gibbs energy of the α-form (**Eqn (1)**) obtained at the DFT-B3LYP/6-31G(*d*,*p*). See text and **Table 2.**  The atoms involved in the torsion angle rotated in the PES are in bold.


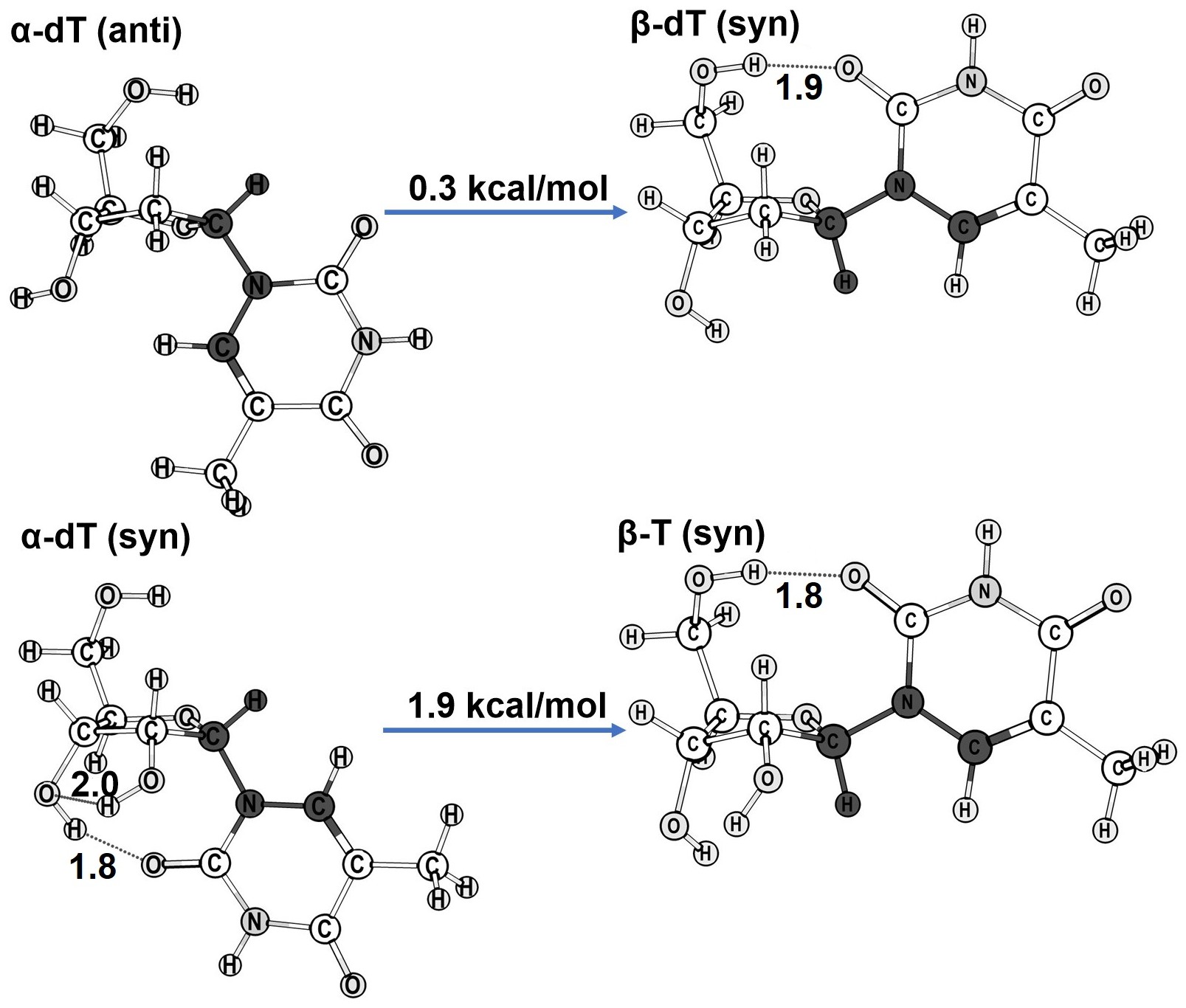


**S. 8** Display of the optimized geometries with bond lengths (in ångströms (Å)) for the studied β- and α-nucleosides of thymine (T) in vacuum. (***Top****)* 2'-deoxythymidine (dT). **(*Bottom)*** Thymidine (T). The energy quoted in kcal/mol is the Gibbs energy of the β-form minus the Gibbs energy of the α-form (**Eqn (1)**) obtained at the DFT-B3LYP/6-31G(*d*,*p*). See text and **Table 2.**  The atoms involved in the torsion angle rotated in the PES are in bold.


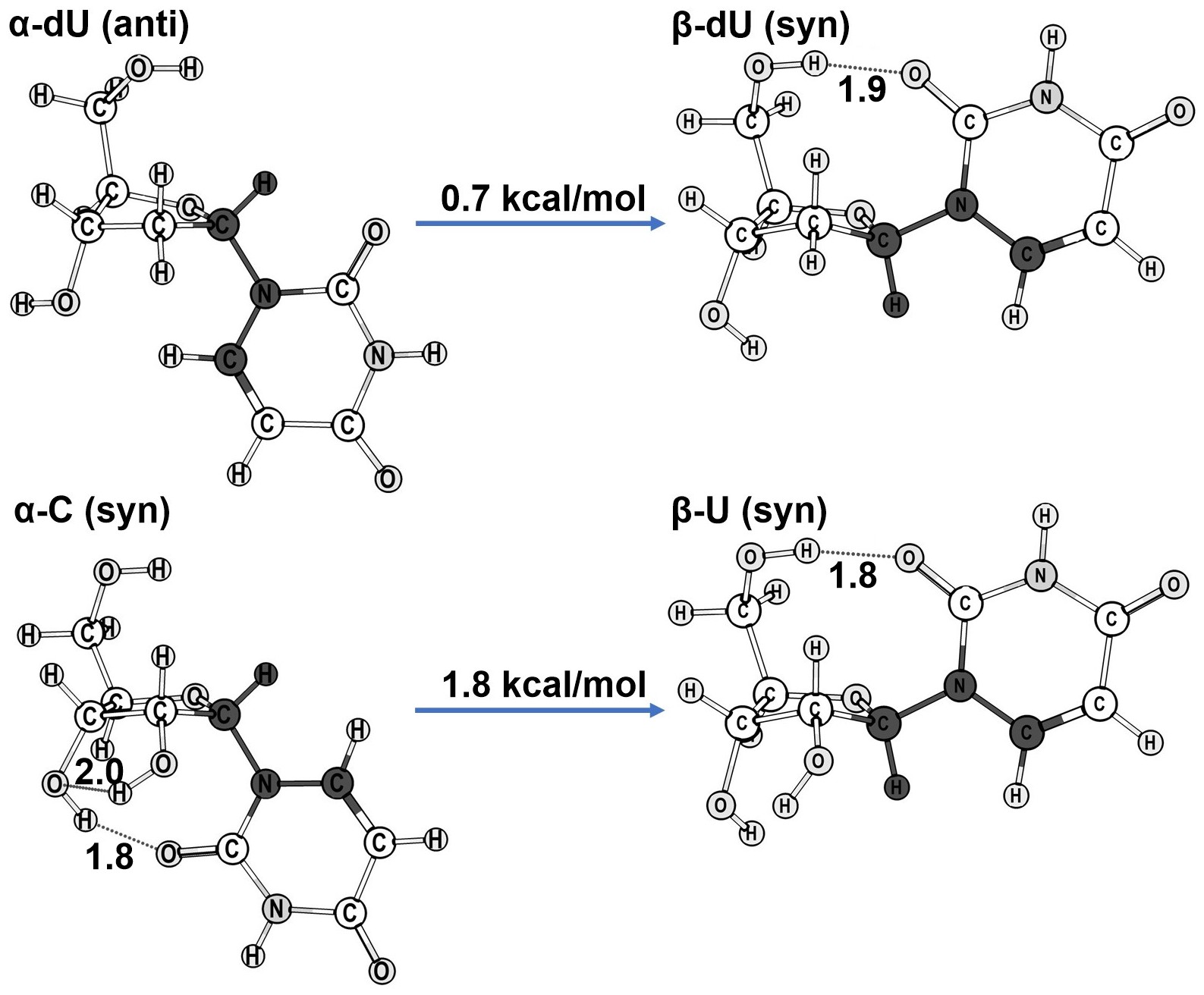


**S. 9** Display of the optimized geometries with bond lengths (in ångströms (Å)) for the studied β- and α-nucleosides of uracil (U) in vacuum. (***Top****)* 2'-deoxyuridine (dG). **(*Bottom)*** Uridine (U). The energy quoted in kcal/mol is the Gibbs energy of the β-form minus the Gibbs energy of the α-form (**Eqn (1)**) obtained at the DFT-B3LYP/6-31G(*d*,*p*). See text and **Table 2.**  The atoms involved in the torsion angle rotated in the PES are in bold.


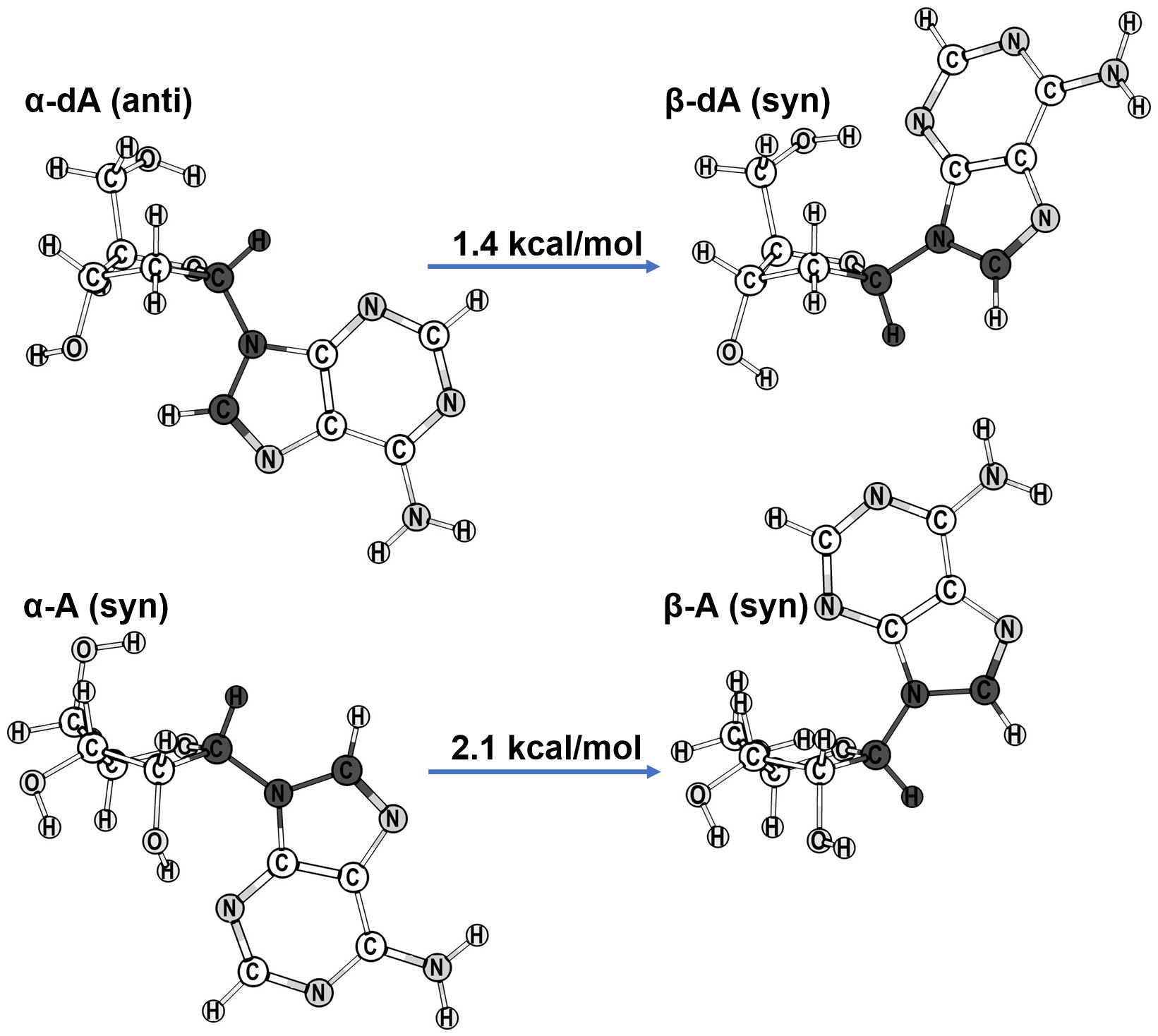


**S. 10** Display of the optimized geometries with bond lengths (in ångströms (Å)) for the studied β- and α-nucleosides of adenine (A) obtained using the IEFPCM model for the aqueous solvation. (***Top****)* 2'-deoxyadenosine (dA). **(*Bottom)*** Adenosine (A). The energy quoted in kcal/mol is the Gibbs energy of the β-form minus the Gibbs energy of the α-form (**Eqn (1)**) obtained at the DFT-B3LYP/6-31G(*d*,*p*). See text and **Table 2.**  The atoms involved in the torsion angle rotated in the PES are in bold.


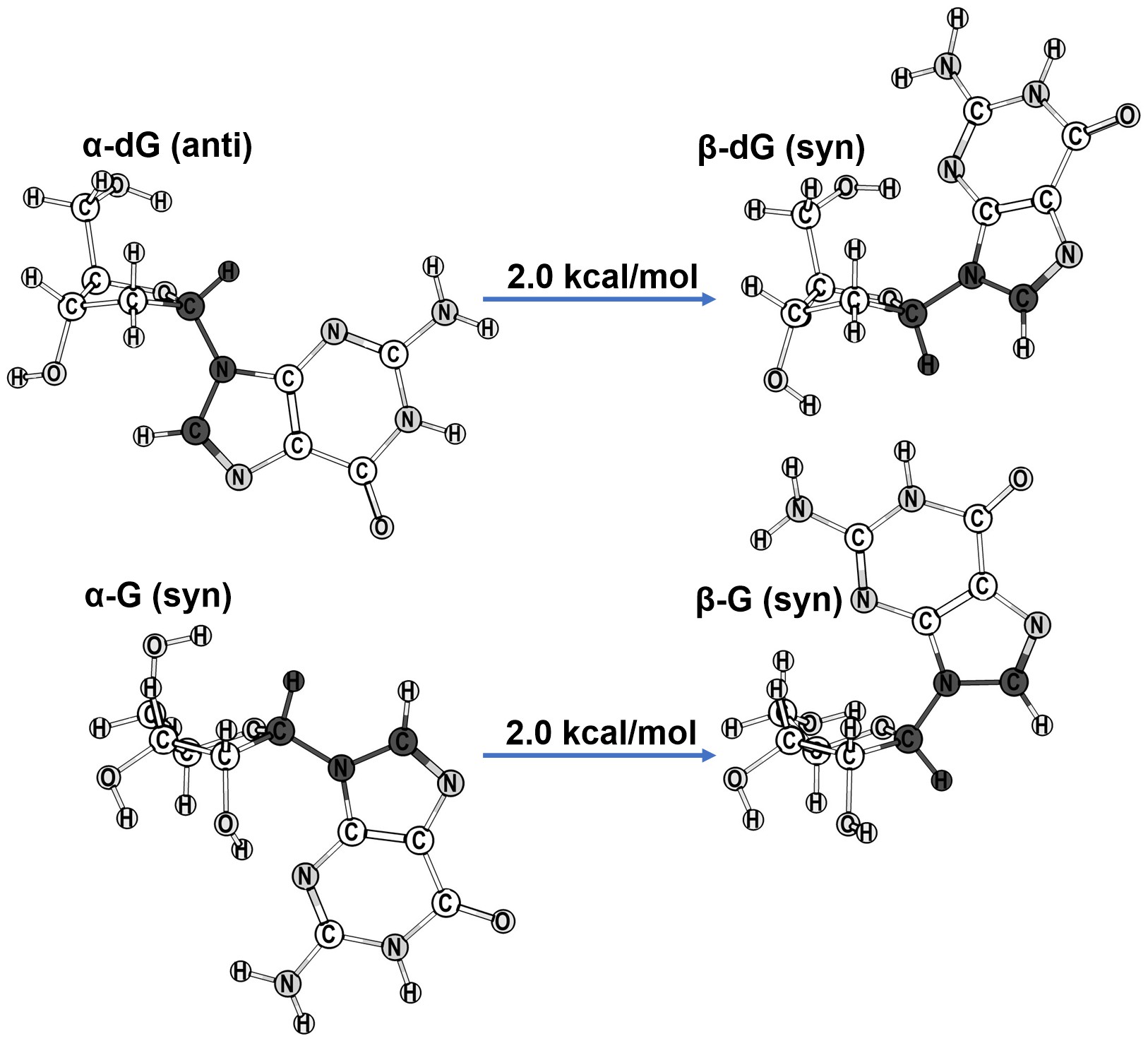


**S. 11** Display of the optimized geometries with bond lengths (in ångströms (Å)) for the studied β- and α-nucleosides of guanine (G) obtained using the IEFPCM model for the aqueous solvation. (***Top****)* 2'-deoxyguanosine (dG). **(*Bottom)*** Guanosine (G). The energy quoted in kcal/mol is the Gibbs energy of the β-form minus the Gibbs energy of the α-form (**Eqn (1)**) obtained at the DFT-B3LYP/6-31G(*d*,*p*). See text and **Table 2.**  The atoms involved in the torsion angle rotated in the PES are in bold.


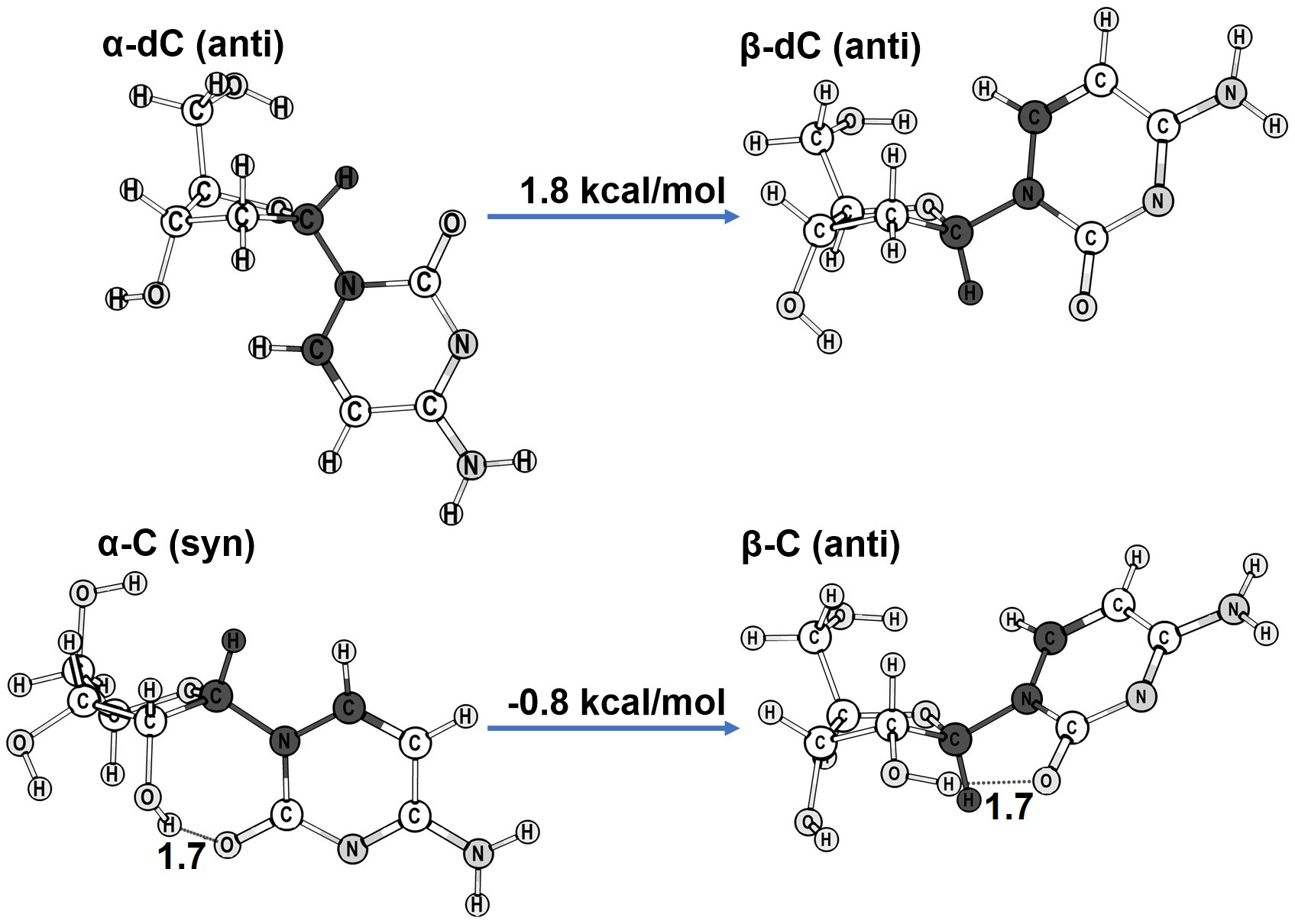


**S. 12** Display of the optimized geometries with bond lengths (in ångströms (Å)) for the studied β- and α-nucleosides of cytosine (C) obtained using the IEFPCM model for the aqueous solvation. (***Top****)* 2'-deoxycytidine (dC). **(*Bottom)*** Cytidine (C). The energy quoted in kcal/mol is the Gibbs energy of the β-form minus the Gibbs energy of the α-form (**Eqn (1)**) obtained at the DFT-B3LYP/6-31G(*d*,*p*). See text and **Table 2.**  The atoms involved in the torsion angle rotated in the PES are in bold.


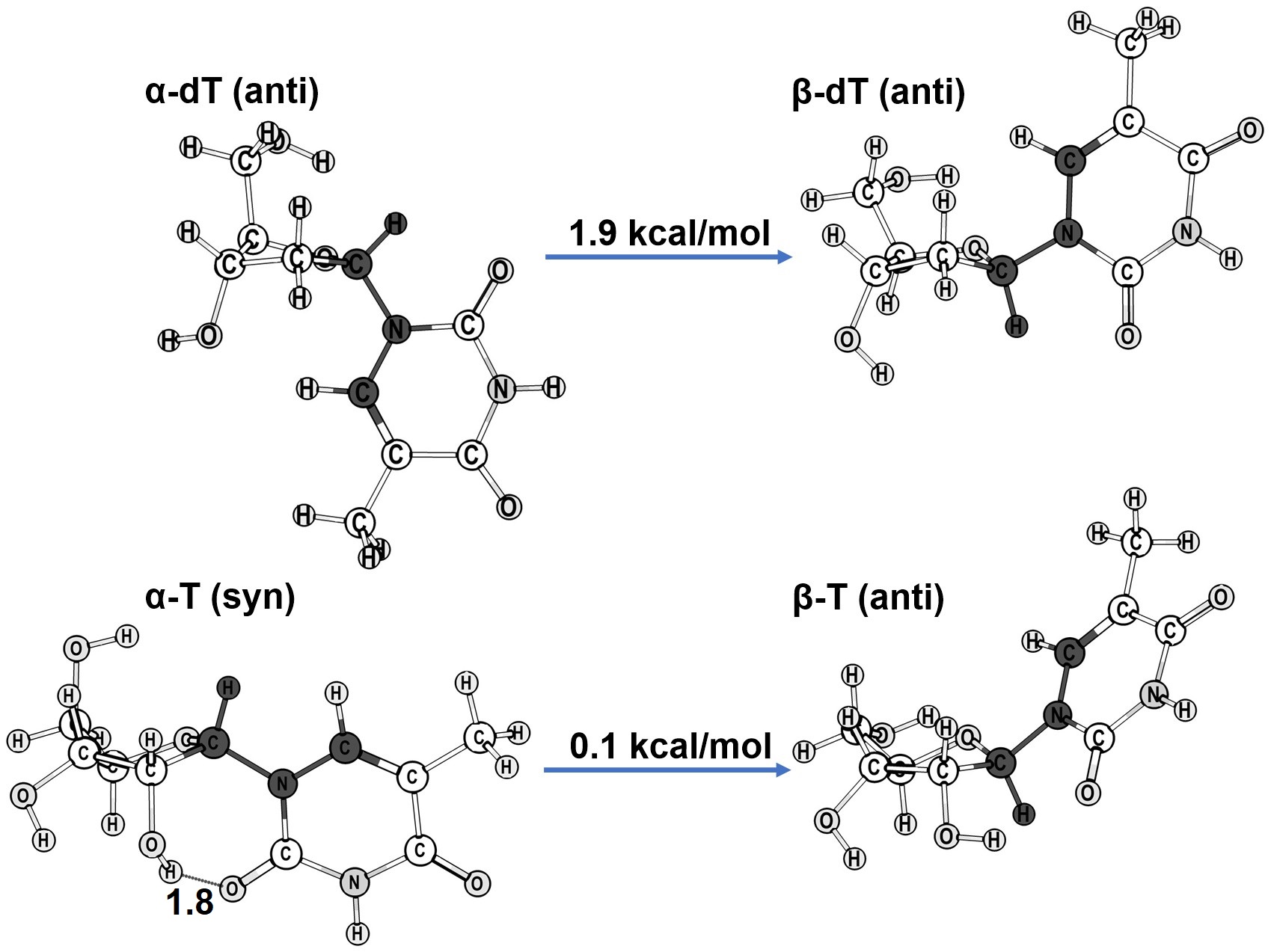


**S. 13** Display of the optimized geometries with bond lengths (in ångströms (Å)) for the studied β- and α-nucleosides of thymine (T) obtained using the IEFPCM model for the aqueous solvation. (***Top****)* 2'-deoxythymidine (dT). **(*Bottom)*** Thymidine (T). The energy quoted in kcal/mol is the Gibbs energy of the β-form minus the Gibbs energy of the α-form (**Eqn (1)**) obtained at the DFT-B3LYP/6-31G(*d*,*p*). See text and **Table 2.**  The atoms involved in the torsion angle rotated in the PES are in bold.


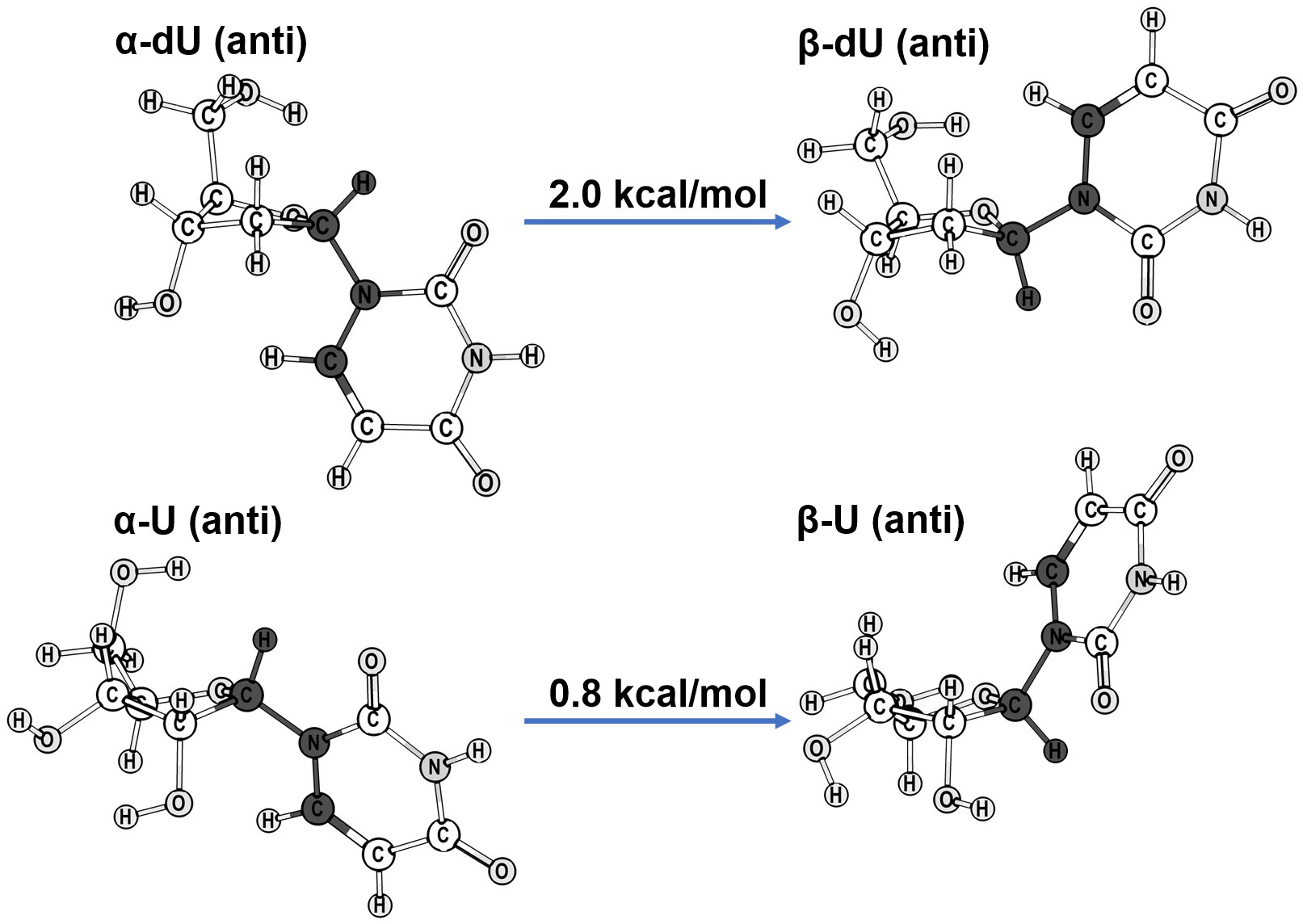


**S. 14** Display of the optimized geometries with bond lengths (in ångströms (Å)) for the studied β- and α-nucleosides of uracil (U) using the IEFPCM model for the water effect. (***Top****)* 2'-deoxyuridine (dU). **(*Bottom)*** Uridine (U). The energy quoted in kcal/mol is the Gibbs energy of the β-form minus the Gibbs energy of the α-form (**Eqn (1)**) obtained at the DFT-B3LYP/6-31G(*d*,*p*). See text and **Table 2.**  The atoms involved in the torsion angle rotated in the PES are in bold.


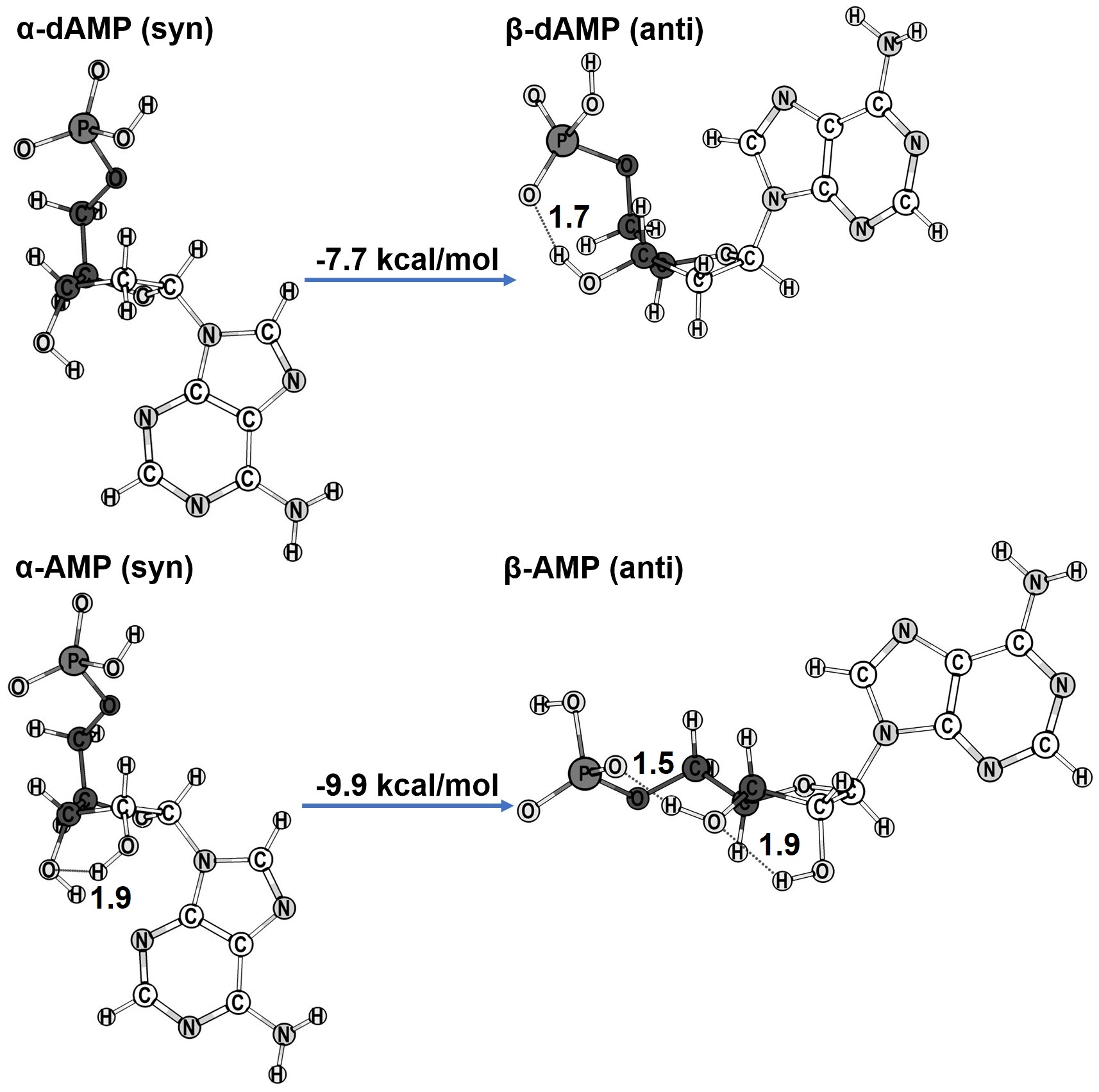


**S. 15** Display of the optimized geometries with bond lengths (in ångströms (Å)) for the studied β- and α-nucleotides of adenine (A) in vacuum obtained for the classic pathway (pathway (a+b), **Fig. 2**). (***Top****)* 2'-deoxyadenosine-5'-monophosphate (dAMP). **(*Bottom)*** Adenosine-5'-monophosphate (AMP). The energy quoted in kcal/mol is the total energy of the β-form minus the total energy of α-form (Eqn (1)) obtained at the DFT-B3LYP/6-31G(*d*,*p*). See text and **Table 3.** The atoms involved in the torsion angle rotated in the PES are in bold.


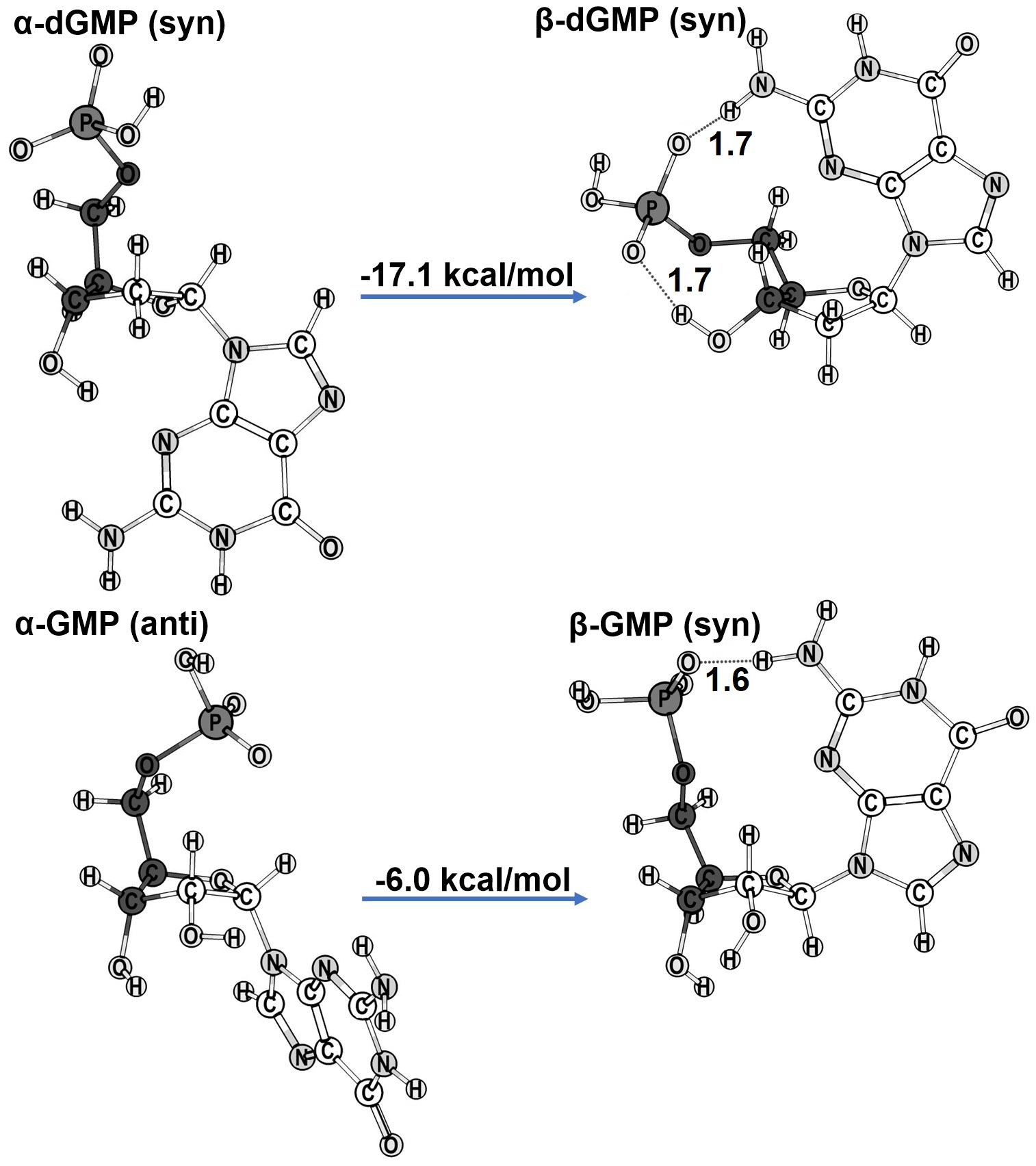


**S. 16** Display of the optimized geometries with bond lengths (in ångströms (Å)) for the studied β- and α-nucleotides of guanine (G) in vacuum obtained for the classic pathway (pathway (a+b), **Fig. 2**). (***Top****)* 2'-deoxyguanosine-5'-monophosphate (dGMP). **(*Bottom)*** Guanosine-5'-monophosphate (GMP). The energy quoted in kcal/mol is the total energy of the β-form minus the total energy of α-form (Eqn (1)) obtained at the DFT-B3LYP/6-31G(*d*,*p*). See text and **Table 3.** The atoms involved in the torsion angle rotated in the PES are in bold.


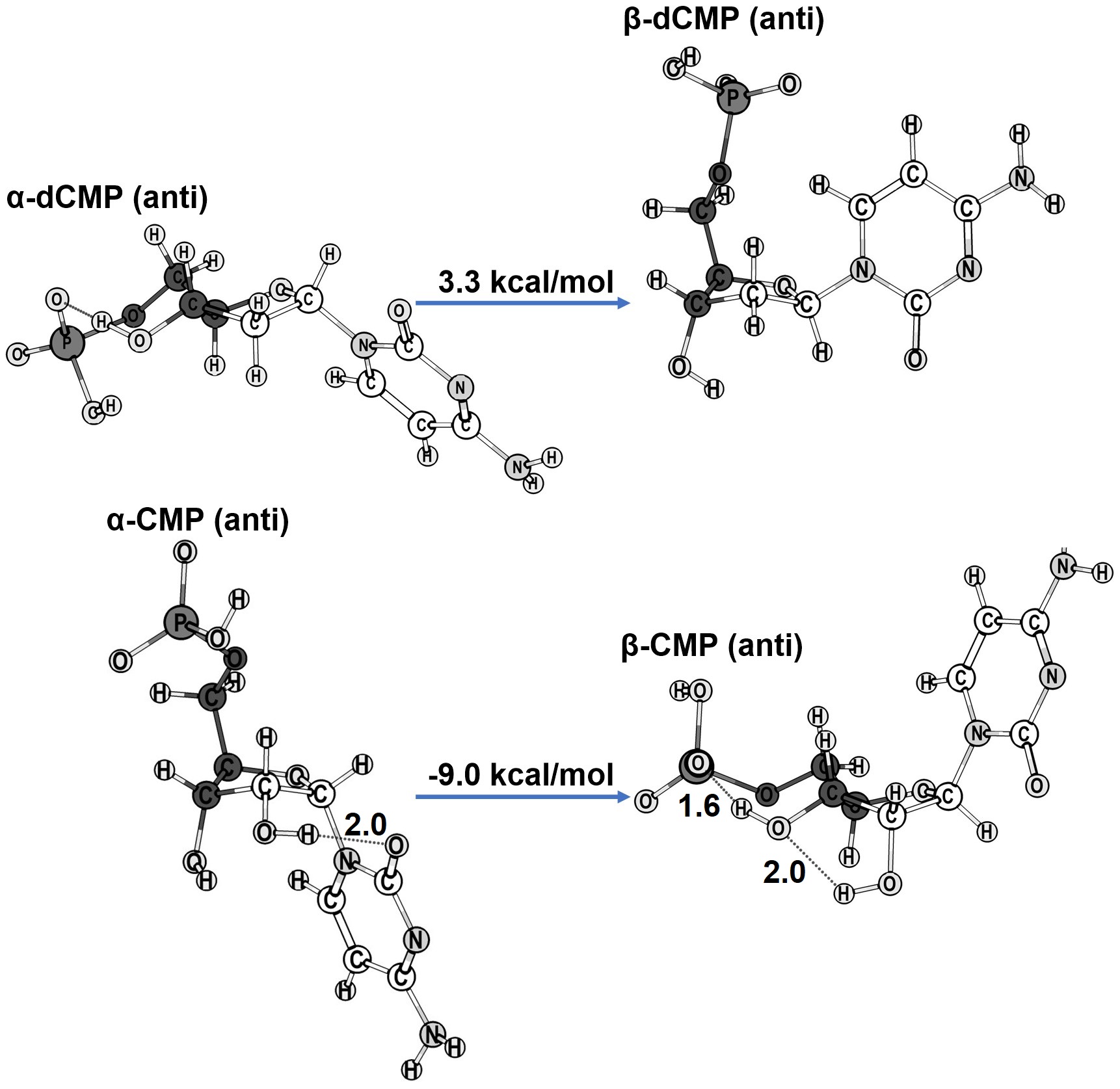


**S. 17** Display of the optimized geometries with bond lengths (in ångströms (Å)) for the studied β- and α-nucleotides of cytosine (C) in vacuum obtained for the classic pathway (pathway (a+b), **Fig. 2**). (***Top****)* 2'-deoxycytidine-5'-monophosphate (dCMP). **(*Bottom)*** Cytidine-5'-monophosphate (CMP). The energy quoted in kcal/mol is the total energy of the β-form minus the total energy of α-form (Eqn (1)) obtained at the DFT-B3LYP/6-31G(*d*,*p*). See text and **Table 3.** The atoms involved in the torsion angle rotated in the PES are in bold.


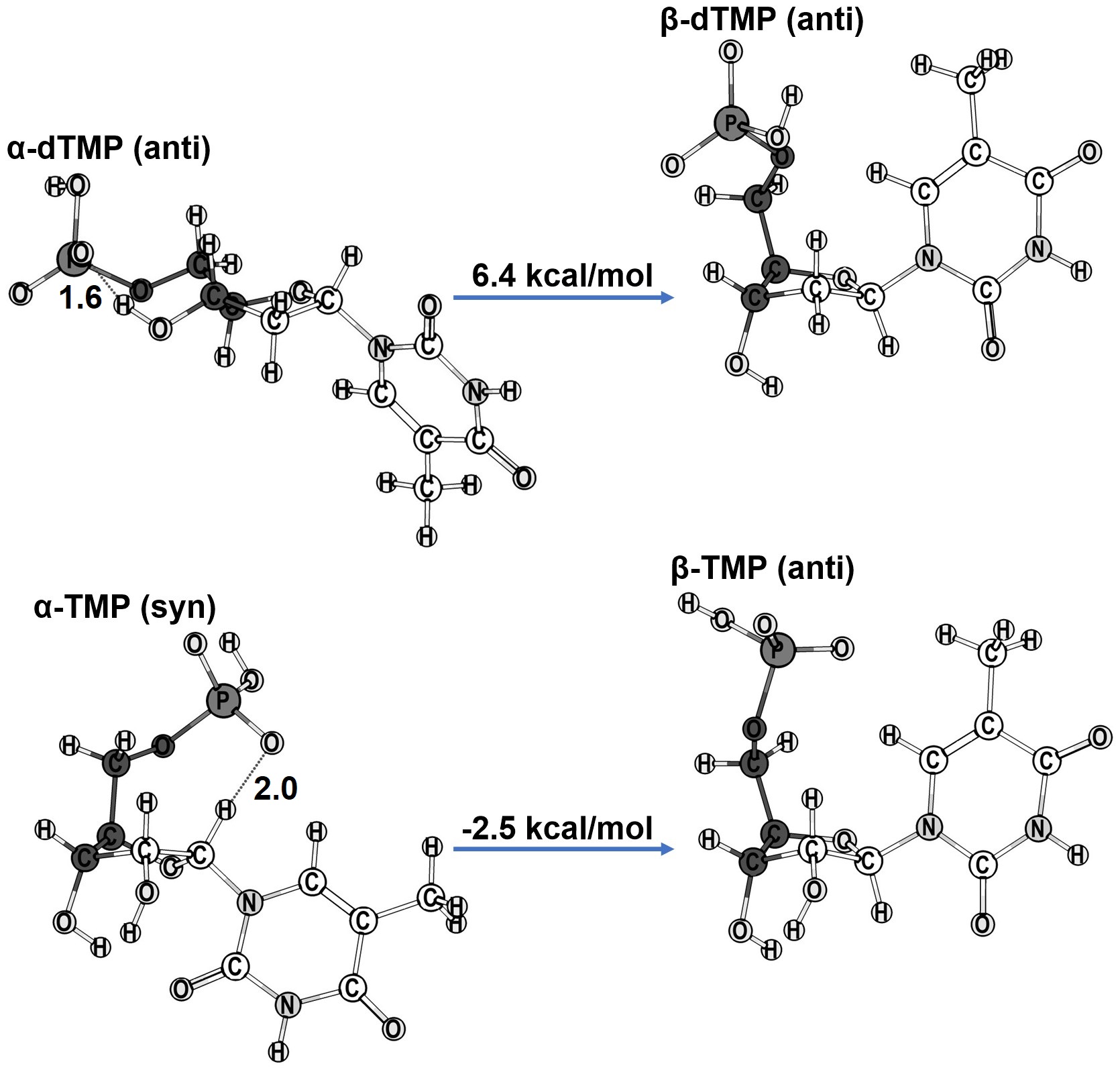


**S. 18** Display of the optimized geometries with bond lengths (in ångströms (Å)) for the studied β- and α-nucleotides of thymine (T) in vacuum obtained for the classic pathway (pathway (a+b), **Fig. 2**). (***Top****)* 2'-deoxythymidine-5'-monophosphate (dTMP). **(*Bottom)*** Thymidine-5'-monophosphate (TMP). The energy quoted in kcal/mol is the total energy of the β-form minus the total energy of α-form (Eqn (1)) obtained at the DFT-B3LYP/6-31G(*d*,*p*). See text and **Table 3.** The atoms involved in the torsion angle rotated in the PES are in bold.


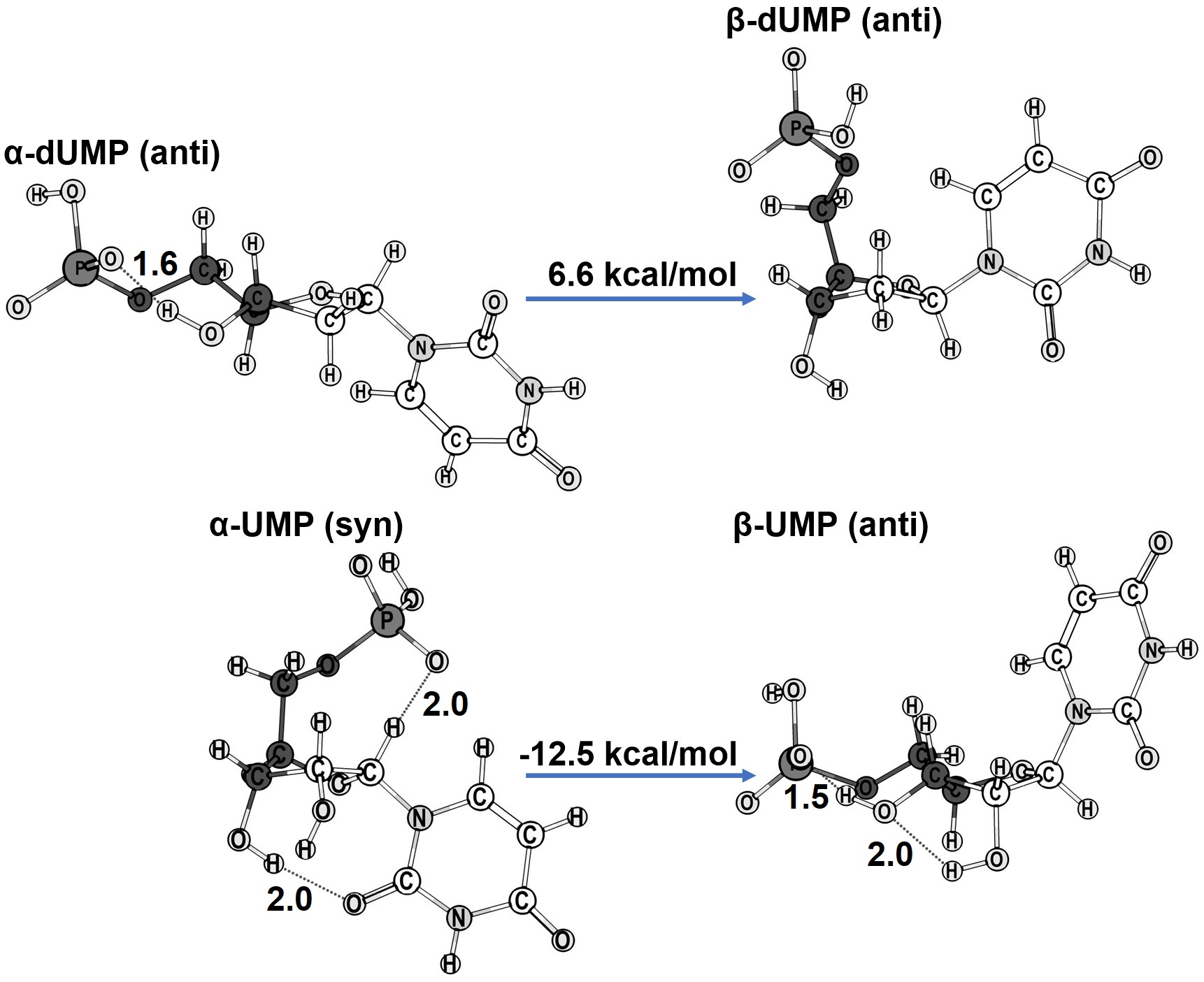


**S. 19** Display of the optimized geometries with bond lengths (in ångströms (Å)) for the studied β- and α-nucleotides of uracil (U) in vacuum obtained for the classic pathway (pathway (a+b), **Fig. 2**). (***Top****)* 2'-deoxyuridine-5'-monophosphate (dUMP). **(*Bottom)*** Uridine-5'-monophosphate (UMP). The energy quoted in kcal/mol is the total energy of the β-form minus the total energy of α-form (Eqn (1)) obtained at the DFT-B3LYP/6-31G(*d*,*p*). See text and **Table 3.** The atoms involved in the torsion angle rotated in the PES are in bold.


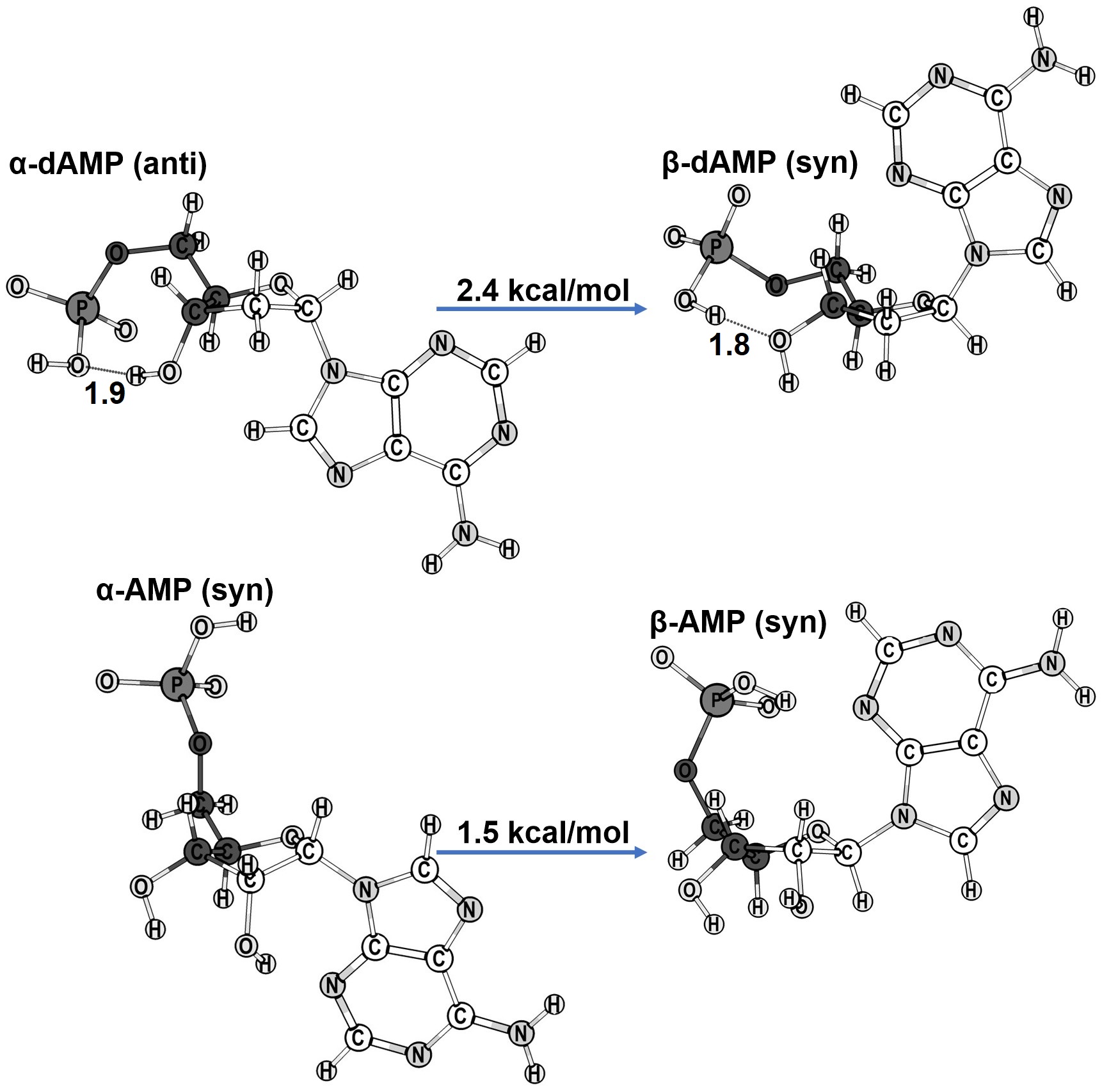


**S. 20** Display of the optimized geometries with bond lengths (in ångströms (Å)) for the studied β- and α-nucleotides of adenine (A) for the classic pathway (pathway (a+b), **Fig. 2**) obtained using the IEFPCM model for the aqueous solvation. (***Top****)* 2'-deoxyadenosine-5'-monophosphate (dAMP). **(*Bottom)*** Adenosine-5'-monophosphate (AMP). The energy quoted in kcal/mol is the total energy of the β-form minus the total energy of α-form (Eqn (1)) obtained at the DFT-B3LYP/6-31G(*d*,*p*). See text and **Table 3.** The atoms involved in the torsion angle rotated in the PES are in bold.


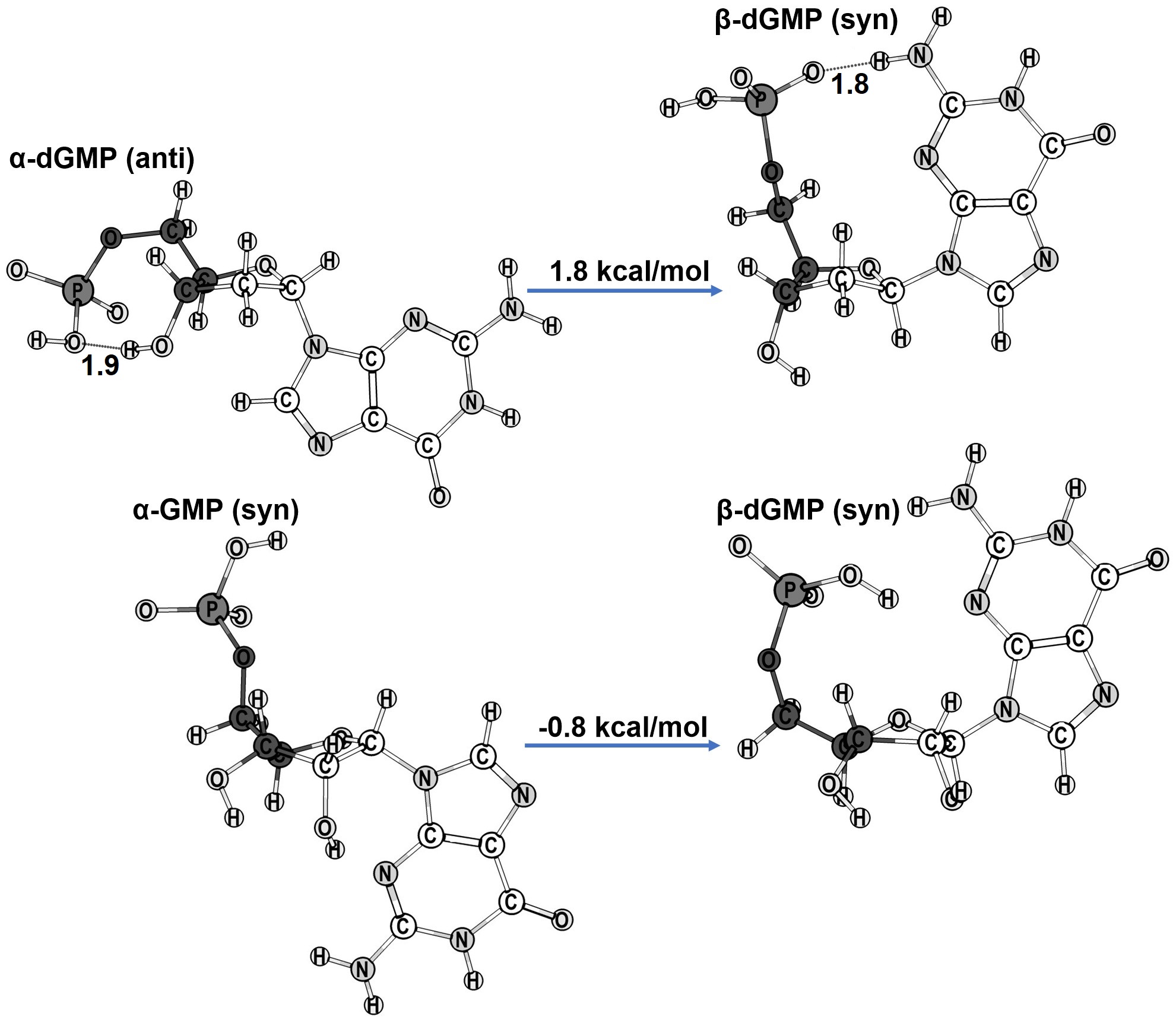


**S. 21** Display of the optimized geometries with bond lengths (in ångströms (Å)) for the studied β- and α-nucleotides of guanine (G) for the classic pathway (pathway (a+b), **Fig. 2**) obtained using the IEFPCM model for the aqueous solvation. (***Top****)* 2'-deoxyguanosine-5'-monophosphate (dGMP). **(*Bottom)*** Guanosine-5'-monophosphate (GMP). The energy quoted in kcal/mol is the total energy of the β-form minus the total energy of α-form (Eqn (1)) obtained at the DFT-B3LYP/6-31G(*d*,*p*). See text and **Table 3.** The atoms involved in the torsion angle rotated in the PES are in bold.


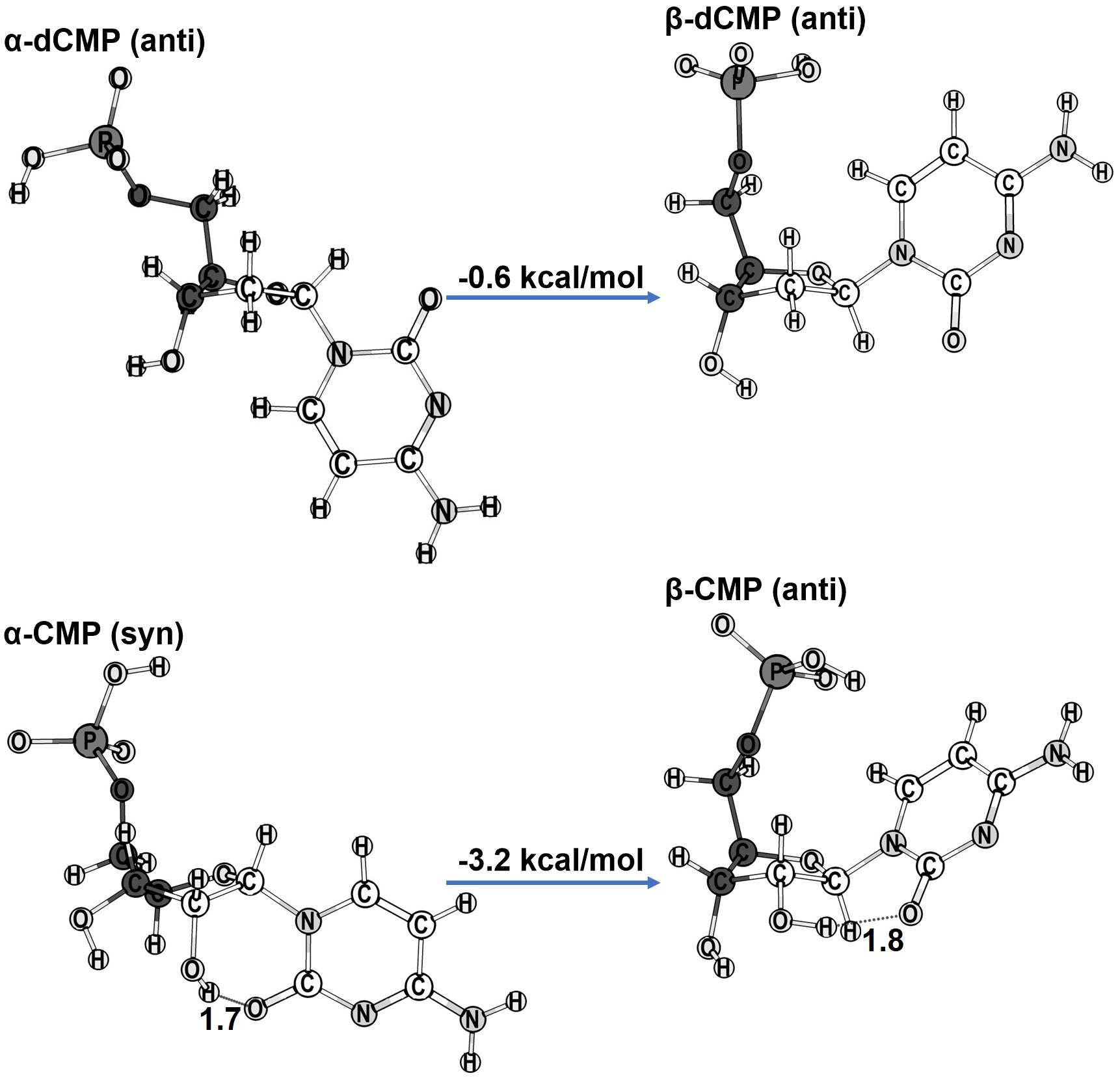


**S. 22** Display of the optimized geometries with bond lengths (in ångströms (Å)) for the studied β- and α-nucleotides of cytosine (C) for the classic pathway (pathway (a+b), **Fig. 2**) obtained using the IEFPCM model for the aqueous solvation. (***Top****)* 2'-deoxycytidine-5'-monophosphate (dCMP). **(*Bottom)*** Cytidine-5'-monophosphate (CMP). The energy quoted in kcal/mol is the total energy of the β-form minus the total energy of α-form (Eqn (1)) obtained at the DFT-B3LYP/6-31G(*d*,*p*). See text and **Table 3.** The atoms involved in the torsion angle rotated in the PES are in bold.


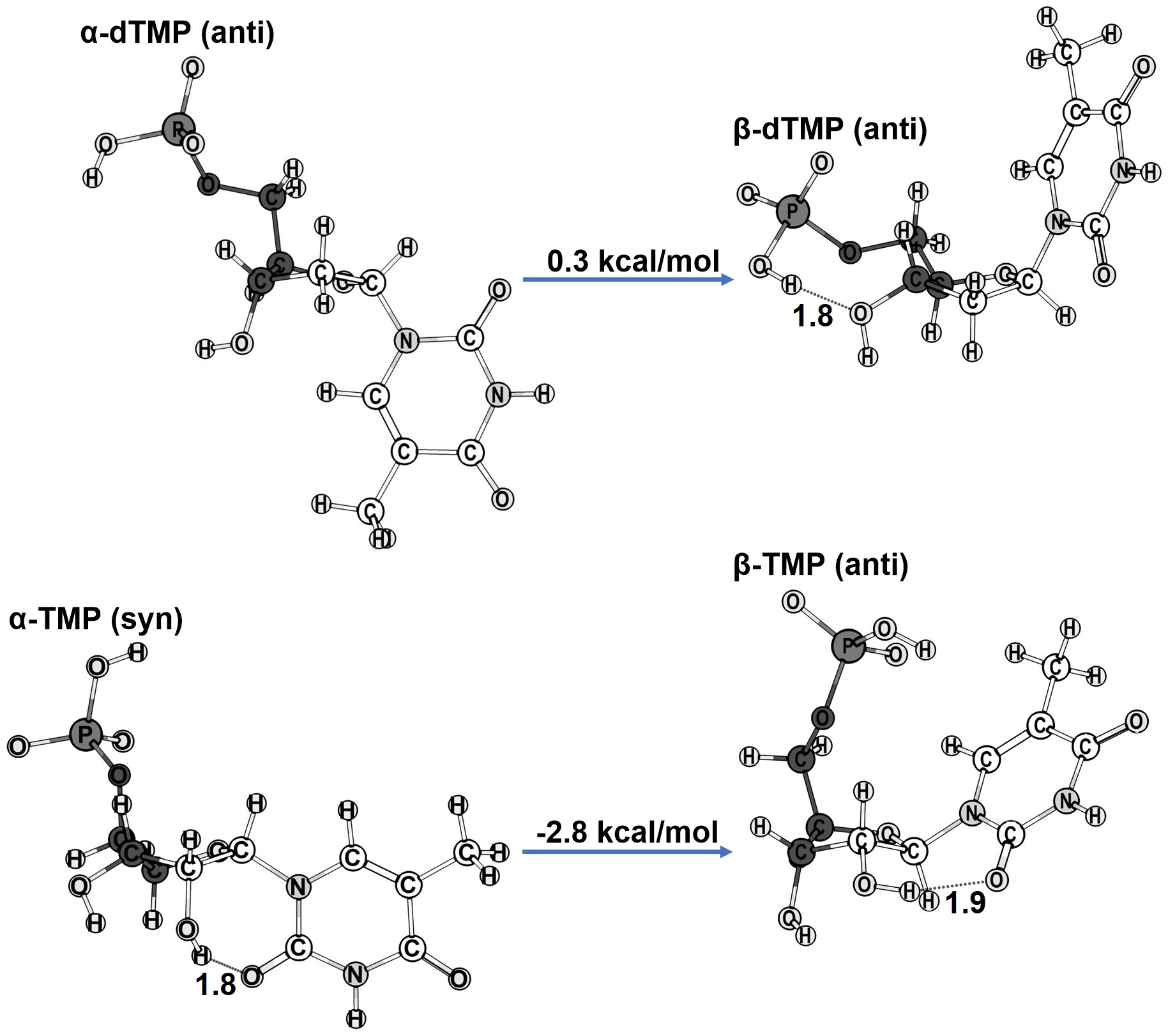


**S. 23** Display of the optimized geometries with bond lengths (in ångströms (Å)) for the studied β- and α-nucleotides of thymine (T) for the classic pathway (pathway (a+b), **Fig. 2**) obtained using the IEFPCM model for the aqueous solvation. (***Top****)* 2'-deoxythymidine-5'-monophosphate (dTMP). **(*Bottom)*** Thymidine-5'-monophosphate (TMP). The energy quoted in kcal/mol is the total energy of the β-form minus the total energy of α-form (Eqn (1)) obtained at the DFT-B3LYP/6-31G(*d*,*p*). See text and **Table 3.** The atoms involved in the torsion angle rotated in the PES are in bold.


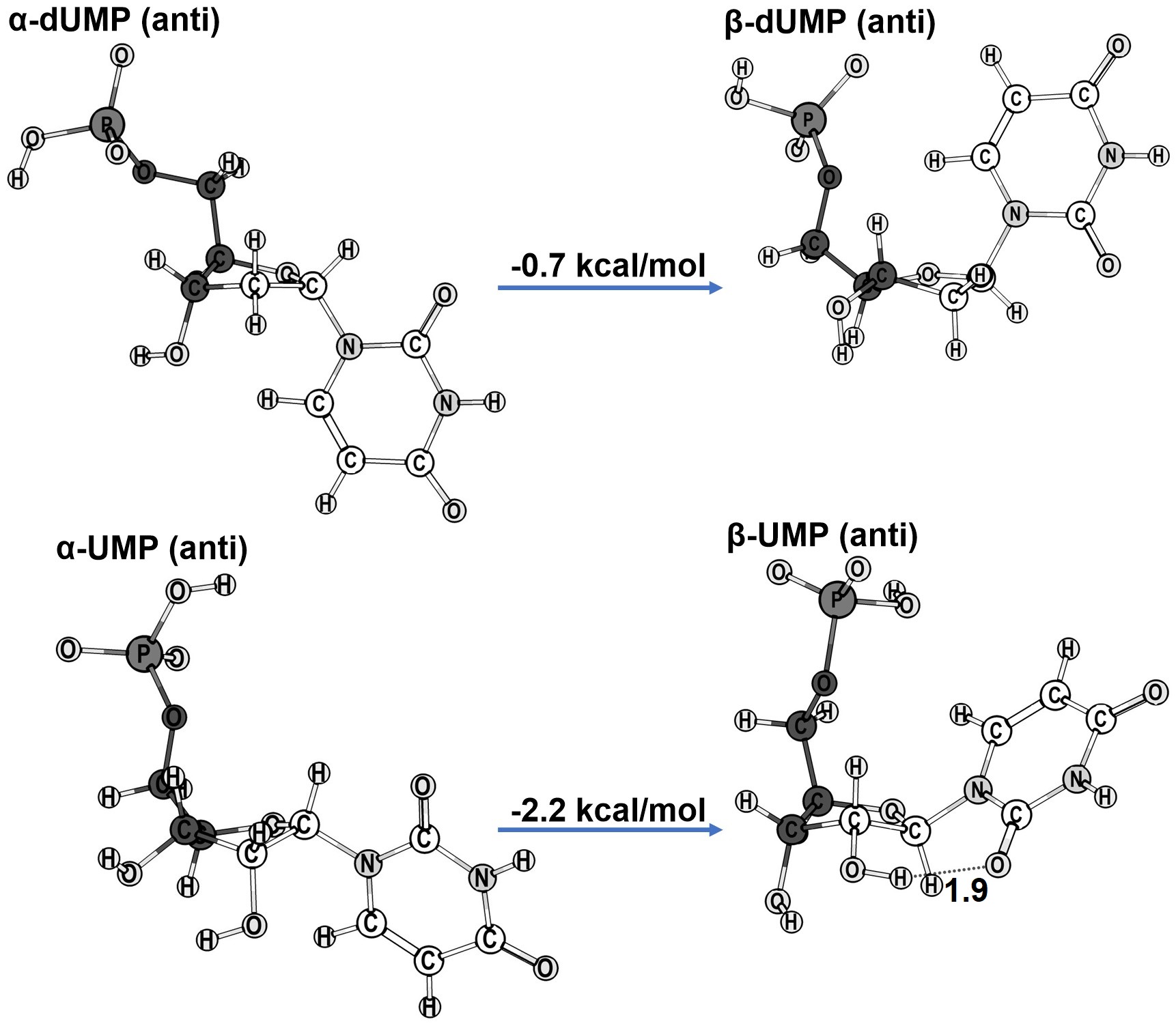


**S. 24** Display of the optimized geometries with bond lengths (in ångströms (Å)) for the studied β- and α-nucleotides of uracil (U) for the classic pathway (pathway (a+b), **Fig. 2**) obtained using the IEFPCM model for the aqueous solvation. (***Top****)* 2'-deoxyuridine-5'-monophosphate (dUMP). **(*Bottom)*** Uridine-5'-monophosphate (UMP). The energy quoted in kcal/mol is the total energy of the β-form minus the total energy of α-form (Eqn (1)) obtained at the DFT-B3LYP/6-31G(*d*,*p*). See text and **Table 3.** The atoms involved in the torsion angle rotated in the PES are in bold.


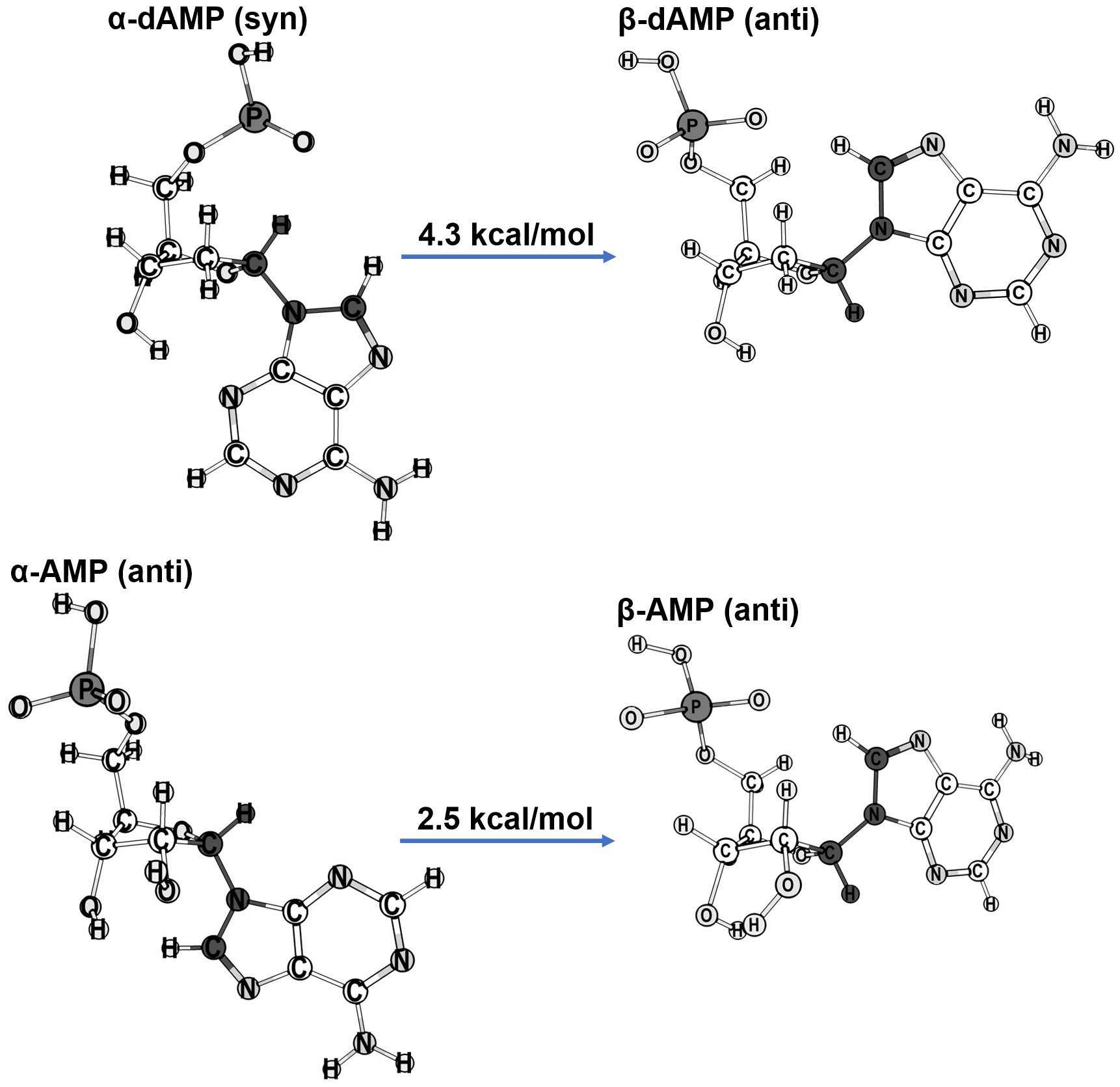


**S. 25** Display of the optimized geometries with bond lengths (in ångströms (Å)) for the studied β- and α-nucleotides of adenine (A) in vacuum for the alternative pathway (pathway (c+d), **Fig. 2**). (***Top****)* 2'-deoxyadenosine-5'-monophosphate (dAMP). **(*Bottom)*** Adenosine-5'-monophosphate (AMP). The energy quoted in kcal/mol is the total energy of the β-form minus the total energy of α-form (Eqn (1)) obtained at the DFT-B3LYP/6-31G(*d*,*p*). See text and **Table 4.** The atoms involved in the torsion angle rotated in the PES are in bold.


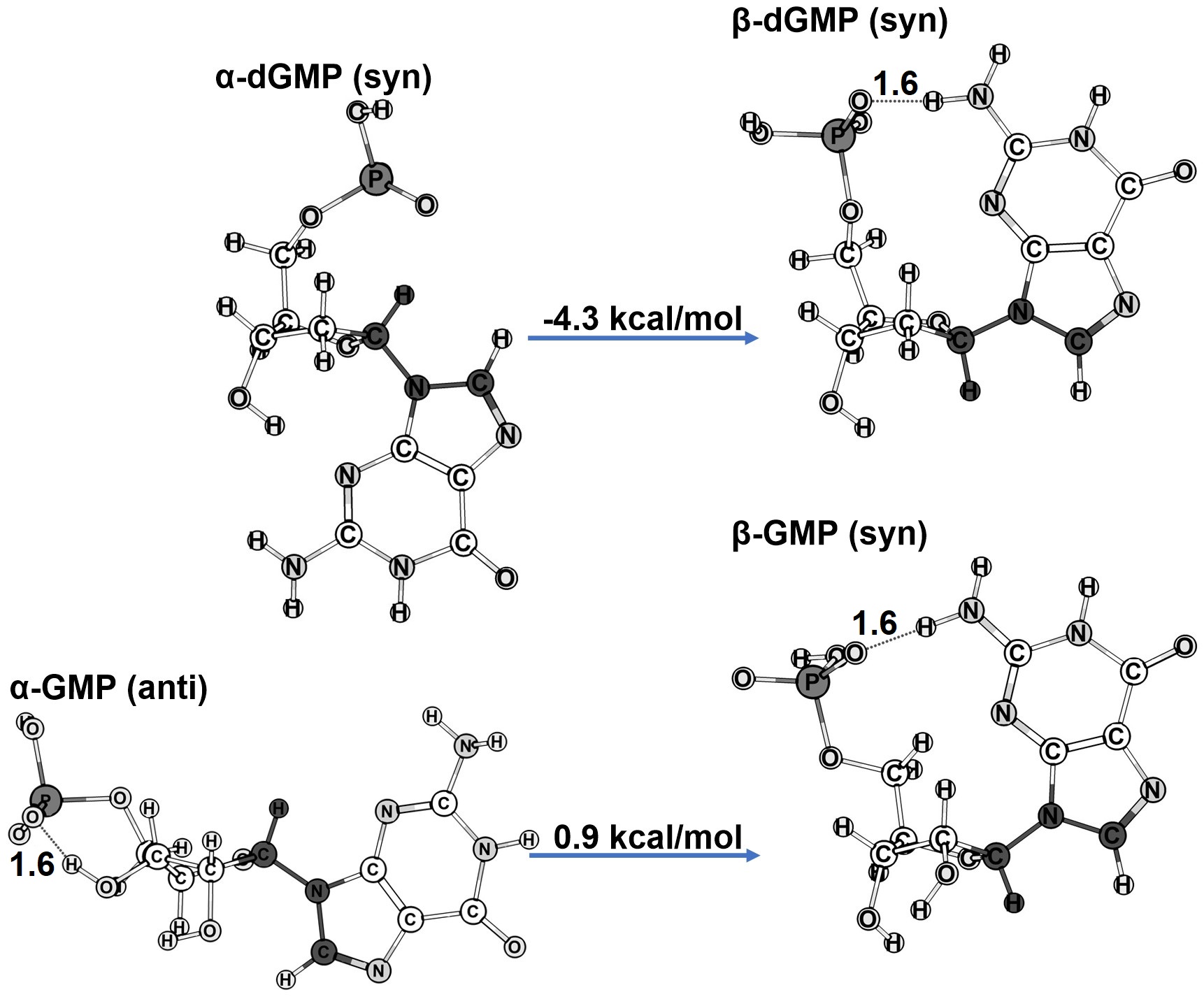


**S. 26** Display of the optimized geometries with bond lengths (in ångströms (Å)) for the studied β- and α-nucleotides of guanine (G) in vacuum for the alternative pathway (pathway (c+d), **Fig. 2**). (***Top****)* 2'-deoxyguanosine-5'-monophosphate (dGMP). **(*Bottom)*** Guanosine-5'-monophosphate (GMP). The energy quoted in kcal/mol is the total energy of the β-form minus the total energy of α-form (Eqn (1)) obtained at the DFT-B3LYP/6-31G(*d*,*p*). See text and **Table 4.** The atoms involved in the torsion angle rotated in the PES are in bold.


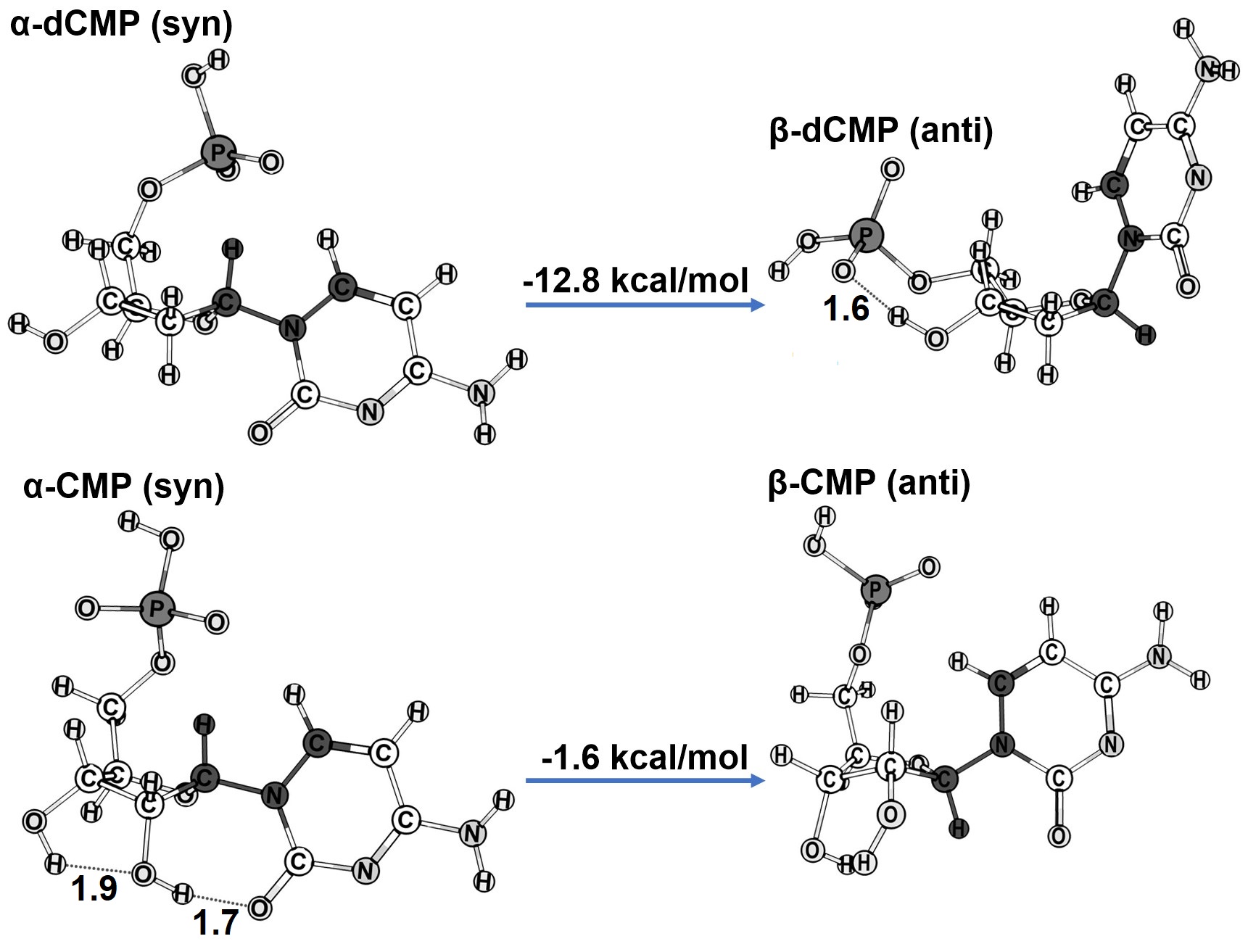


**S. 27** Display of the optimized geometries with bond lengths (in ångströms (Å)) for the studied β- and α-nucleotides of cytosine (C) in vacuum for the alternative pathway (pathway (c+d), **Fig. 2**). (***Top****)* 2'-deoxycytidine-5'-monophosphate (dCMP). **(*Bottom)*** Cytidine-5'-monophosphate (CMP). The energy quoted in kcal/mol is the total energy of the β-form minus the total energy of α-form (Eqn (1)) obtained at the DFT-B3LYP/6-31G(*d*,*p*). See text and **Table 4.** The atoms involved in the torsion angle rotated in the PES are in bold.


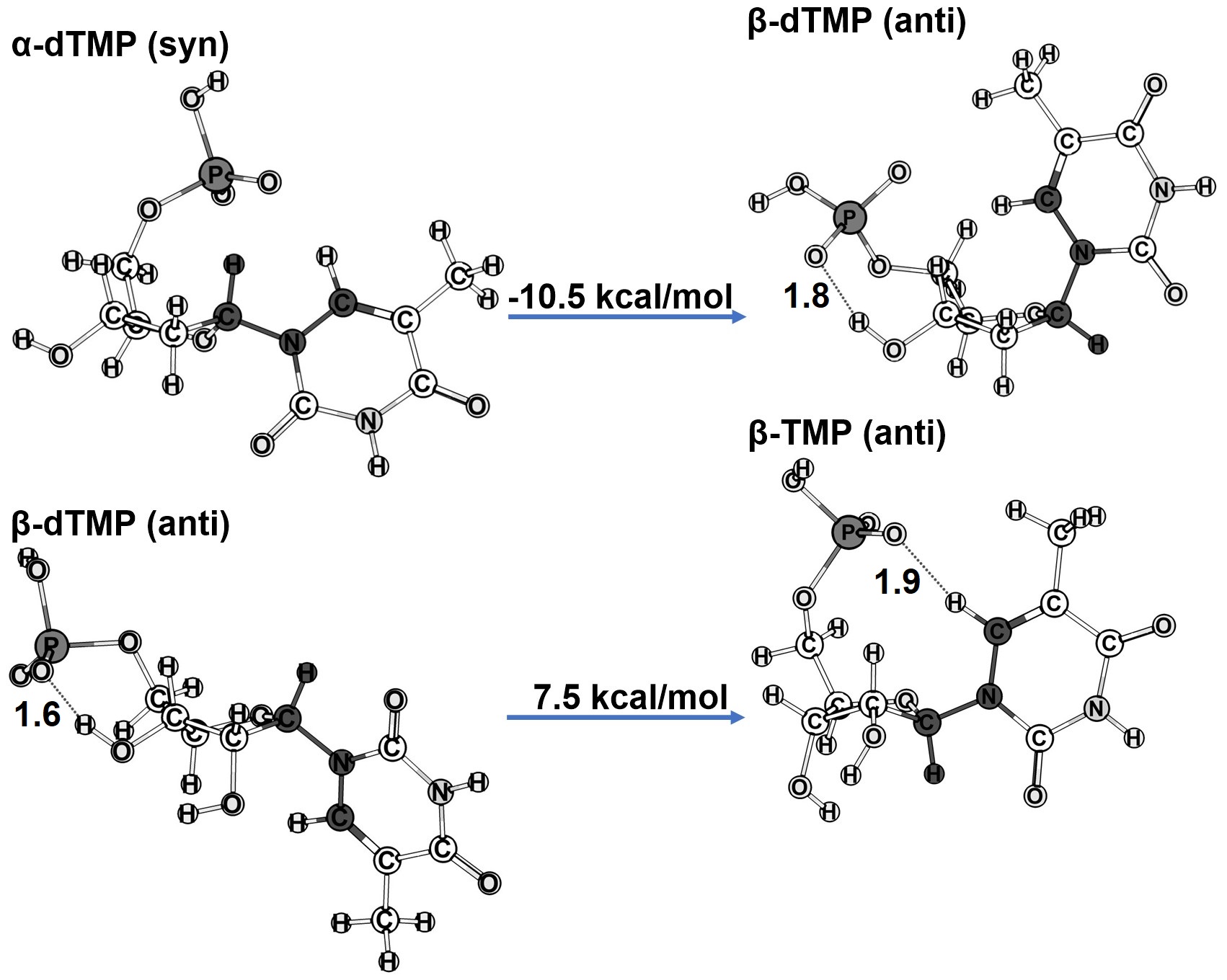


**S. 28** Display of the optimized geometries with bond lengths (in ångströms (Å)) for the studied β- and α-nucleotides of thymine (T) in vacuum for the alternative pathway (pathway (c+d), **Fig. 2**). (***Top****)* 2'-deoxythymidine-5'-monophosphate (dTMP). **(*Bottom)*** Thymidine-5'-monophosphate (TMP). The energy quoted in kcal/mol is the total energy of the β-form minus the total energy of α-form (Eqn (1)) obtained at the DFT-B3LYP/6-31G(*d*,*p*). See text and **Table 4.** The atoms involved in the torsion angle rotated in the PES are in bold.


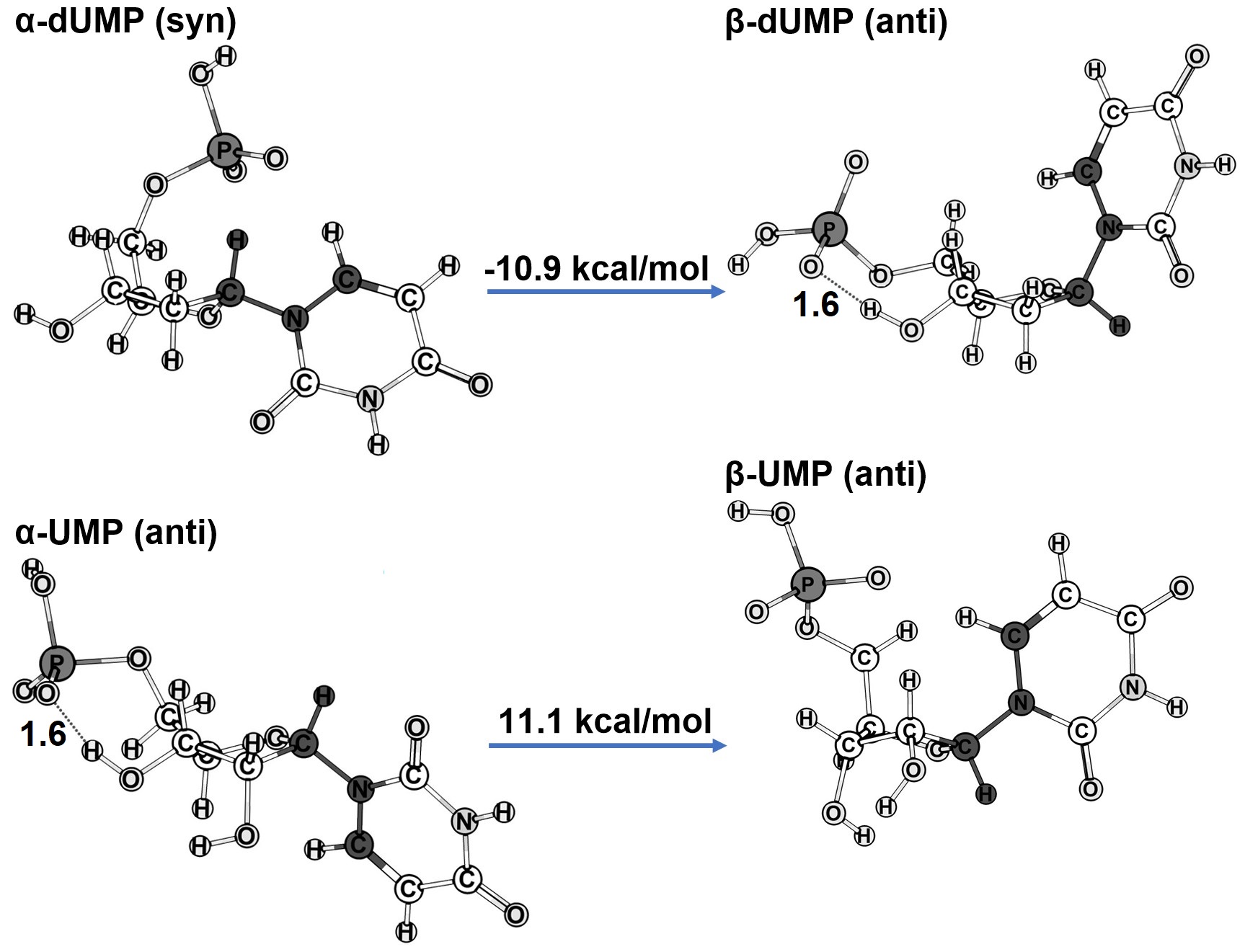


**S. 29** Display of the optimized geometries with bond lengths (in ångströms (Å)) for the studied β- and α-nucleotides of uracil (U) in vacuum for the alternative pathway (pathway (c+d), **Fig. 2**). (***Top****)* 2'-deoxyuridine-5'-monophosphate (dUMP). **(*Bottom)*** Uridine-5'-monophosphate (UMP). The energy quoted in kcal/mol is the total energy of the β-form minus the total energy of α-form (Eqn (1)) obtained at the DFT-B3LYP/6-31G(*d*,*p*). See text and **Table 4.** The atoms involved in the torsion angle rotated in the PES are in bold.


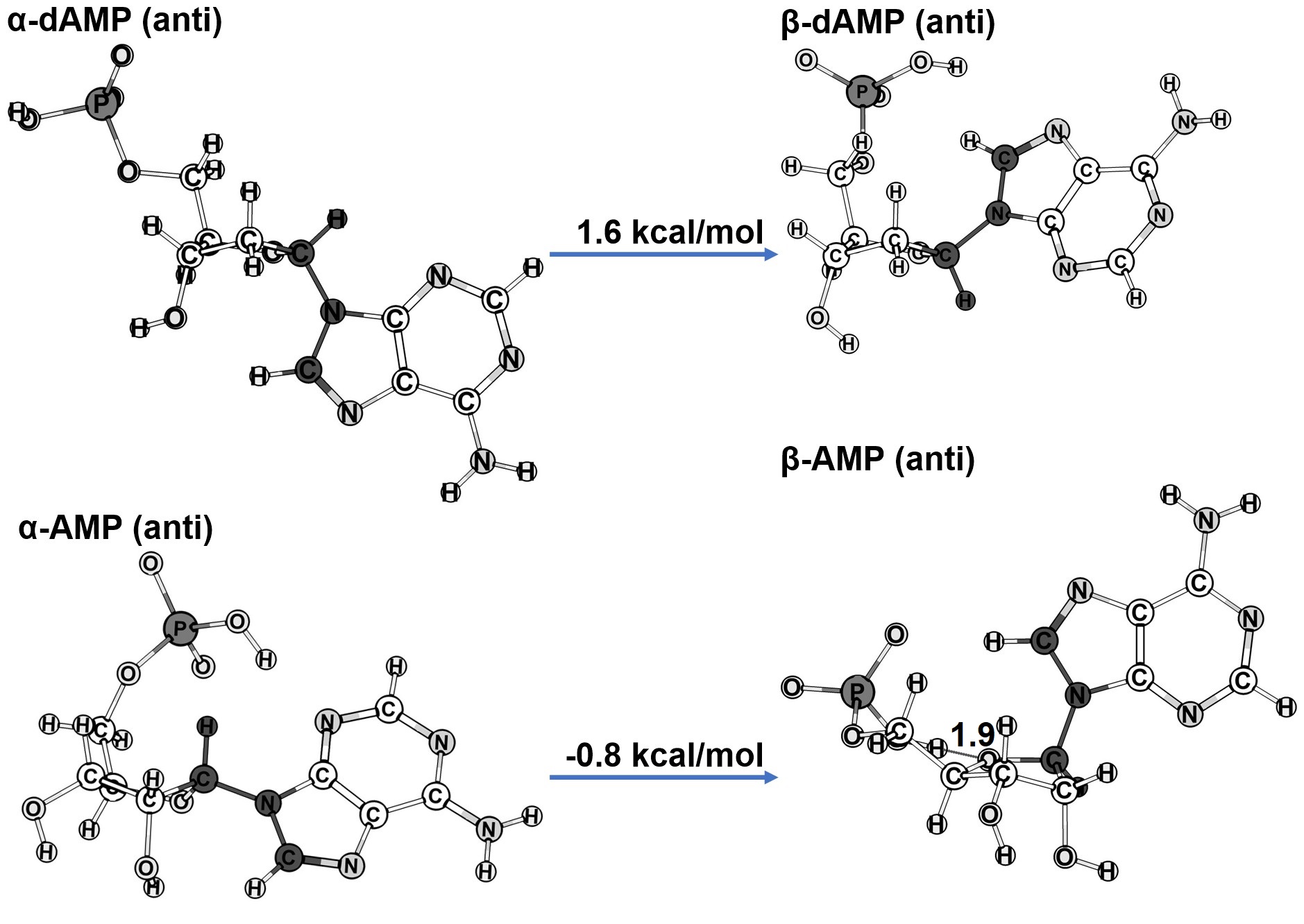


**S. 30** Display of the optimized geometries with bond lengths (in ångströms (Å)) for the studied β- and α-nucleotides of adenine (A) for the alternative pathway (pathway (c+d), **Fig. 2**) obtained using the IEFPCM model for the aqueous solvation. (***Top****)* 2'-deoxyadenosine-5'-monophosphate (dAMP). **(*Bottom)*** Adenosine-5'-monophosphate (AMP). The energy quoted in kcal/mol is the total energy of the β-form minus the total energy of α-form (Eqn (1)) obtained at the DFT-B3LYP/6-31G(*d*,*p*). See text and **Table 4.** The atoms involved in the torsion angle rotated in the PES are in bold.


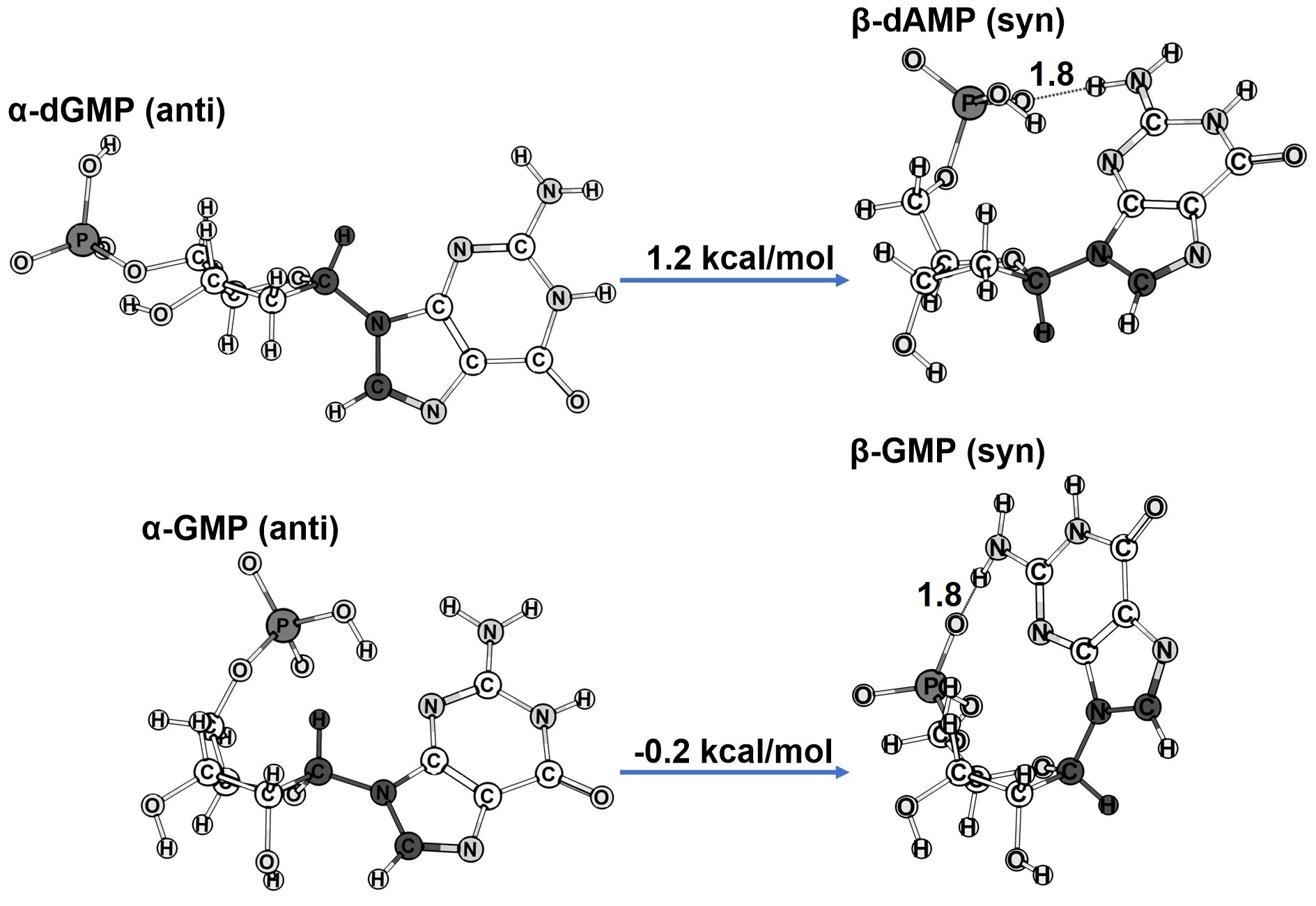


**S. 31** Display of the optimized geometries with bond lengths (in ångströms (Å)) for the studied β- and α-nucleotides of guanine (G) for the alternative pathway (pathway (c+d), **Fig. 2**) obtained using the IEFPCM model for the aqueous solvation. (***Top****)* 2'-deoxyguanosine-5'-monophosphate (dGMP). **(*Bottom)*** Guanosine-5'-monophosphate (GMP). The energy quoted in kcal/mol is the total energy of the β-form minus the total energy of α-form (Eqn (1)) obtained at the DFT-B3LYP/6-31G(*d*,*p*). See text and **Table 4.** The atoms involved in the torsion angle rotated in the PES are in bold.


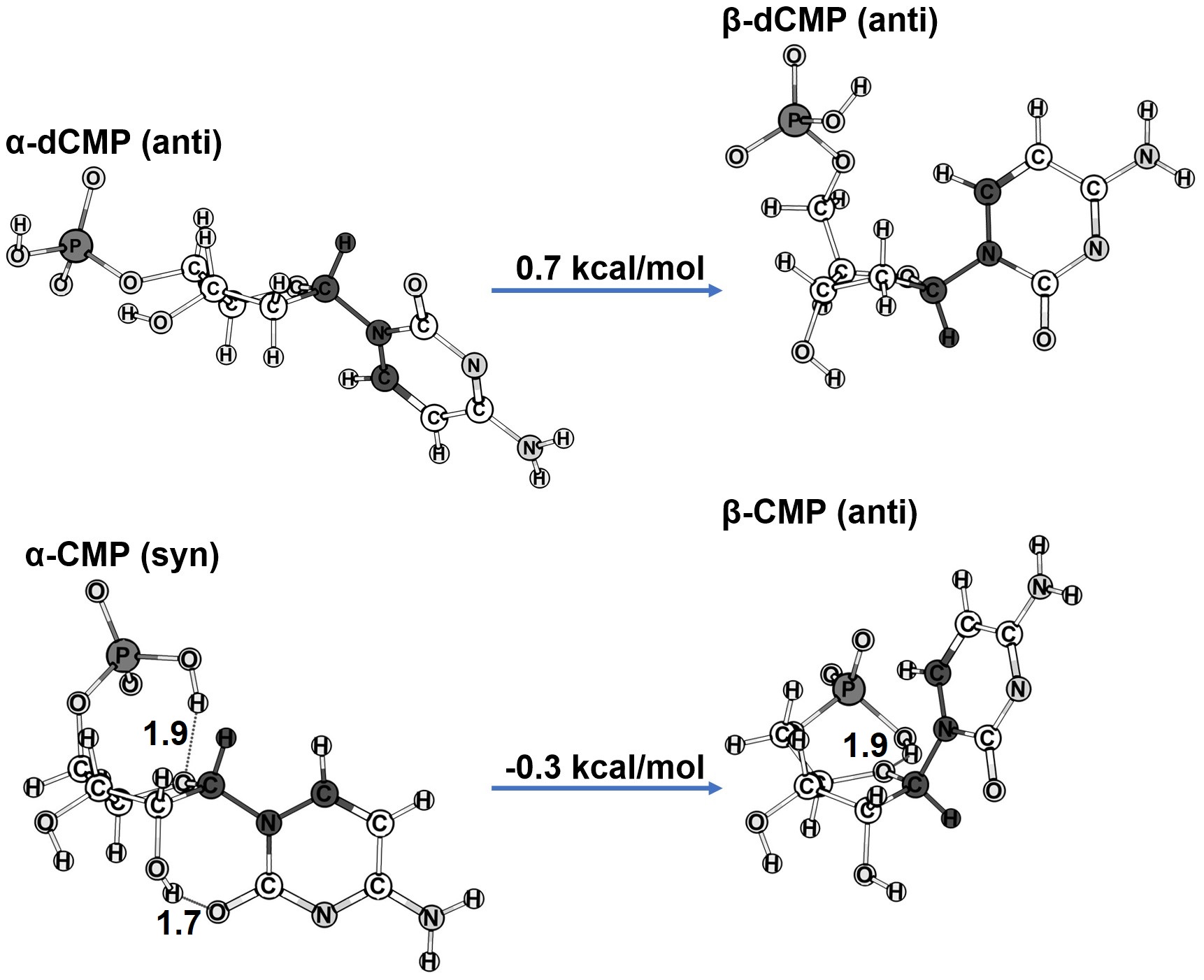


**S. 32** Display of the optimized geometries with bond lengths (in ångströms (Å)) for the studied β- and α-nucleotides of cytosine (C) for the alternative pathway (pathway (c+d), **Fig. 2**) obtained using the IEFPCM model for the aqueous solvation. (***Top****)* 2'-deoxycytidine-5'-monophosphate (dCMP). **(*Bottom)*** Cytidine-5'-monophosphate (CMP). The energy quoted in kcal/mol is the total energy of the β-form minus the total energy of α-form (Eqn (1)) obtained at the DFT-B3LYP/6-31G(*d*,*p*). See text and **Table 4.** The atoms involved in the torsion angle rotated in the PES are in bold.


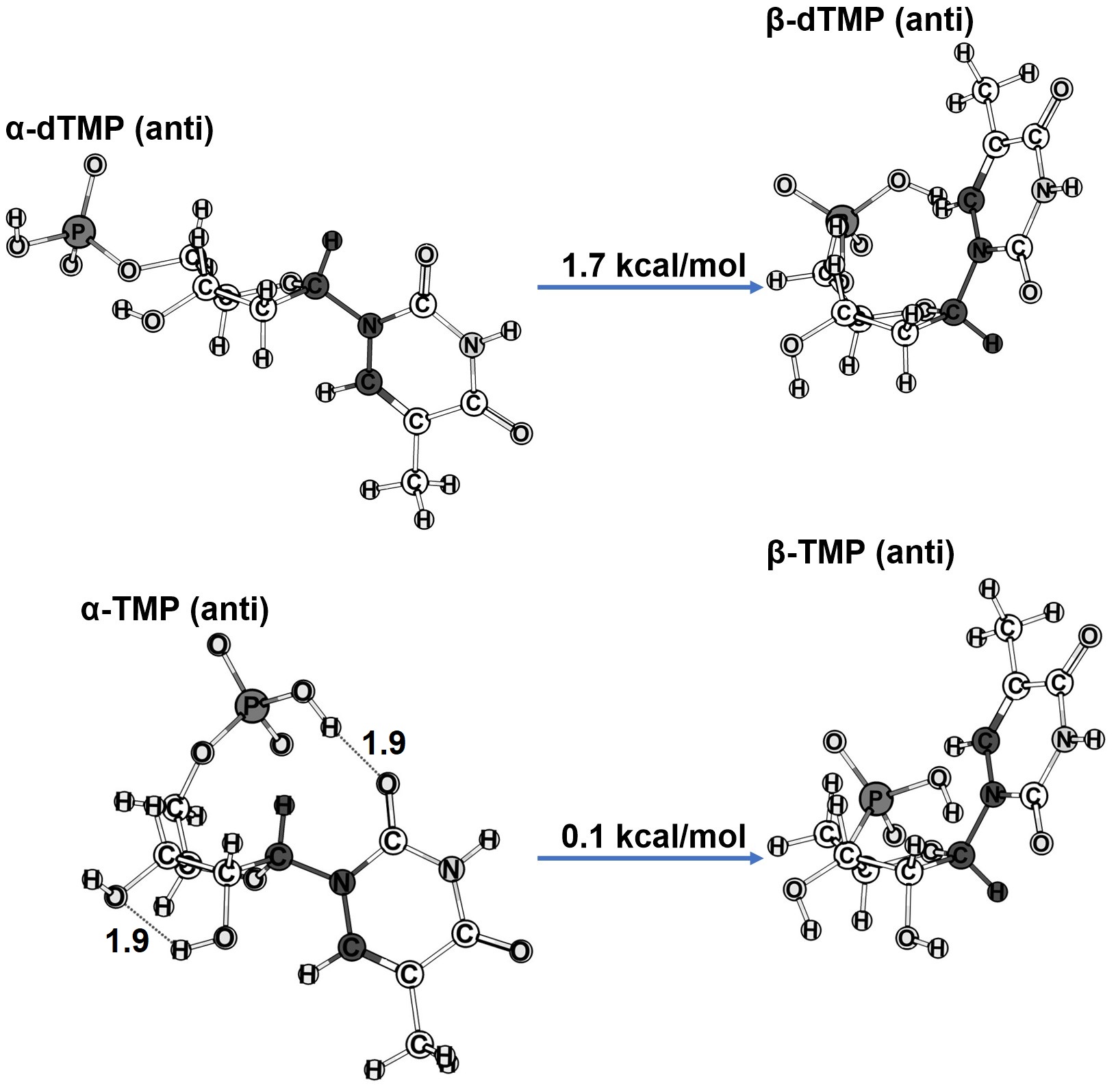


**S. 33** Display of the optimized geometries with bond lengths (in ångströms (Å)) for the studied β- and α-nucleotides of thymine (T) for the alternative pathway (pathway (c+d), **Fig. 2**) obtained using the IEFPCM model for the aqueous solvation. (***Top****)* 2'-deoxythymidine-5'-monophosphate (dTMP). **(*Bottom)*** Thymidine-5'-monophosphate (TMP). The energy quoted in kcal/mol is the total energy of the β-form minus the total energy of α-form (Eqn (1)) obtained at the DFT-B3LYP/6-31G(*d*,*p*). See text and **Table 4.** The atoms involved in the torsion angle rotated in the PES are in bold.


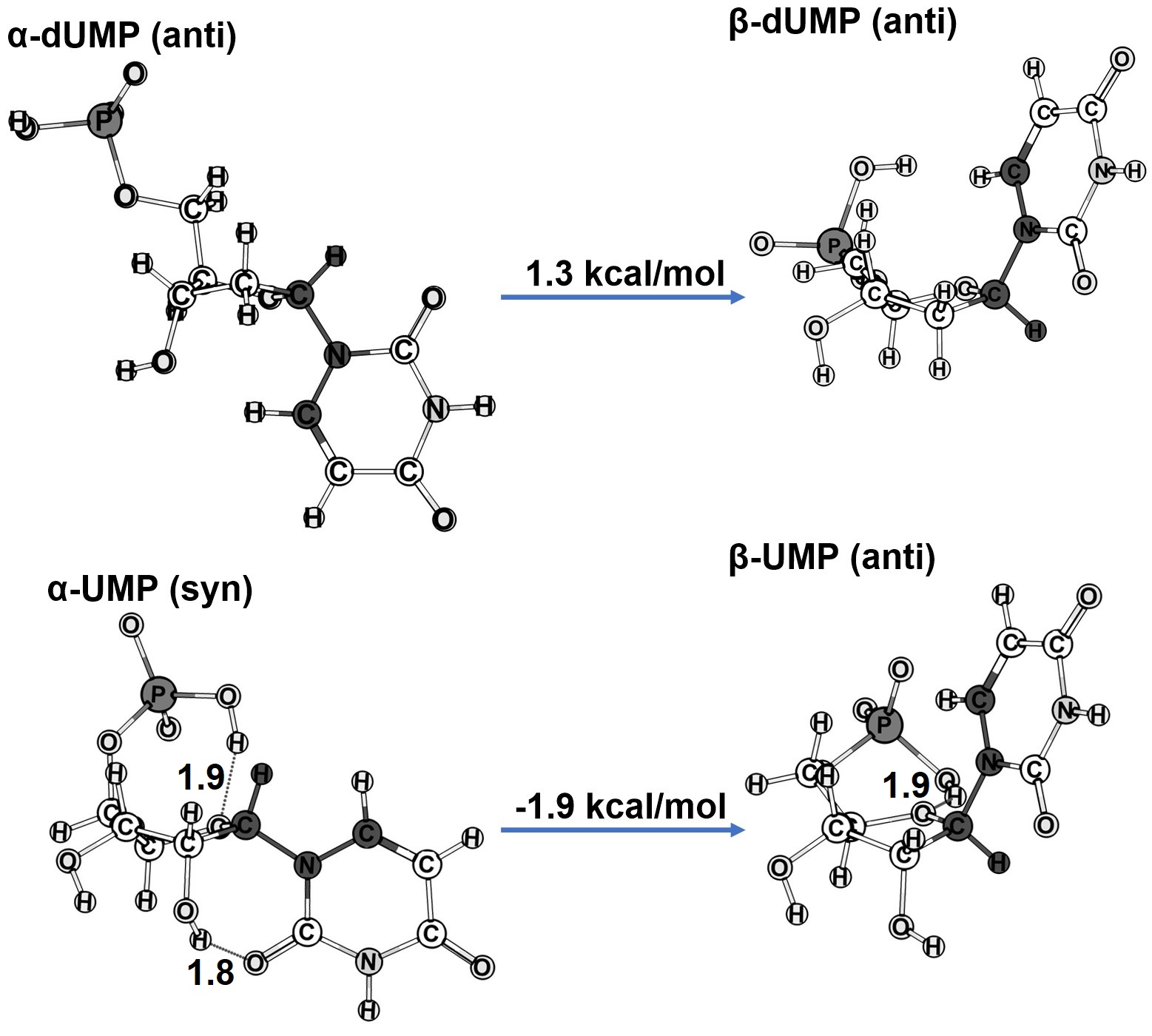


**S. 34** Display of the optimized geometries with bond lengths (in ångströms (Å)) for the studied β- and α-nucleotides of uracil (U) for the alternative pathway (pathway (c+d), **Fig. 2**) obtained using the IEFPCM model for the aqueous solvation. (***Top****)* 2'-deoxyuridine-5'-monophosphate (dUMP). **(*Bottom)*** Uridine-5'-monophosphate (UMP). The energy quoted in kcal/mol is the total energy of the β-form minus the total energy of α-form (Eqn (1)) obtained at the DFT-B3LYP/6-31G(*d*,*p*). See text and **Table 4.** The atoms involved in the torsion angle rotated in the PES are in bold.
